# Supplementary material for: SLC25A45 is required for mitochondrial uptake of methylated amino acids and de novo carnitine biosynthesis
Source: Mol Cell. 2025 Nov 6;85(21):4093–4104.e8. doi: 10.1016/j.molcel.2025.08.018 (PMC12594208; doi:10.1016/j.molcel.2025.08.018)
Supplement: Document S1. Figures S1–S8 and Tables S4 and S5 [file mmc1.pdf]

Molecular Cell, Volume *85*

Supplemental information

SLC25A45 is required for mitochondrial uptake of methylated amino acids and *de novo* carnitine biosynthesis

Marilia M. Dias, Martin S. King, Engy Shokry, Sergio Lilla, Nikki Paul, Peter Thomason, Sara Zanivan, David Sumpton, Edmund R.S. Kunji, and Thomas MacVicar

A

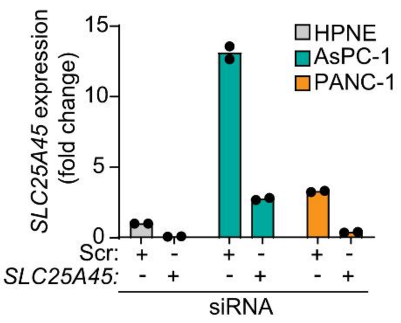

B

ICE Analysis of AsPC-1 *SLC25A45* KO clones

Clone	Guide Target	PAM sequence	Indel %	Model Fit (R^2)	Knockout-Score
#1	GATGAAGTAGATCCCCACCG	TGG	95	0.95	95
#2	GATGAAGTAGATCCCCACCG	TGG	92	0.92	92
#3	GATGAAGTAGATCCCCACCG	TGG	92	0.92	92
#4	GATGAAGTAGATCCCCACCG	TGG	91	0.91	70

Relative contribution of each sequence (normalised)

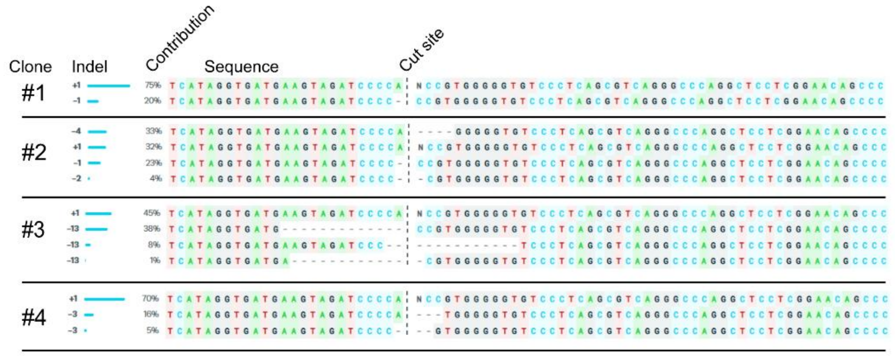

C

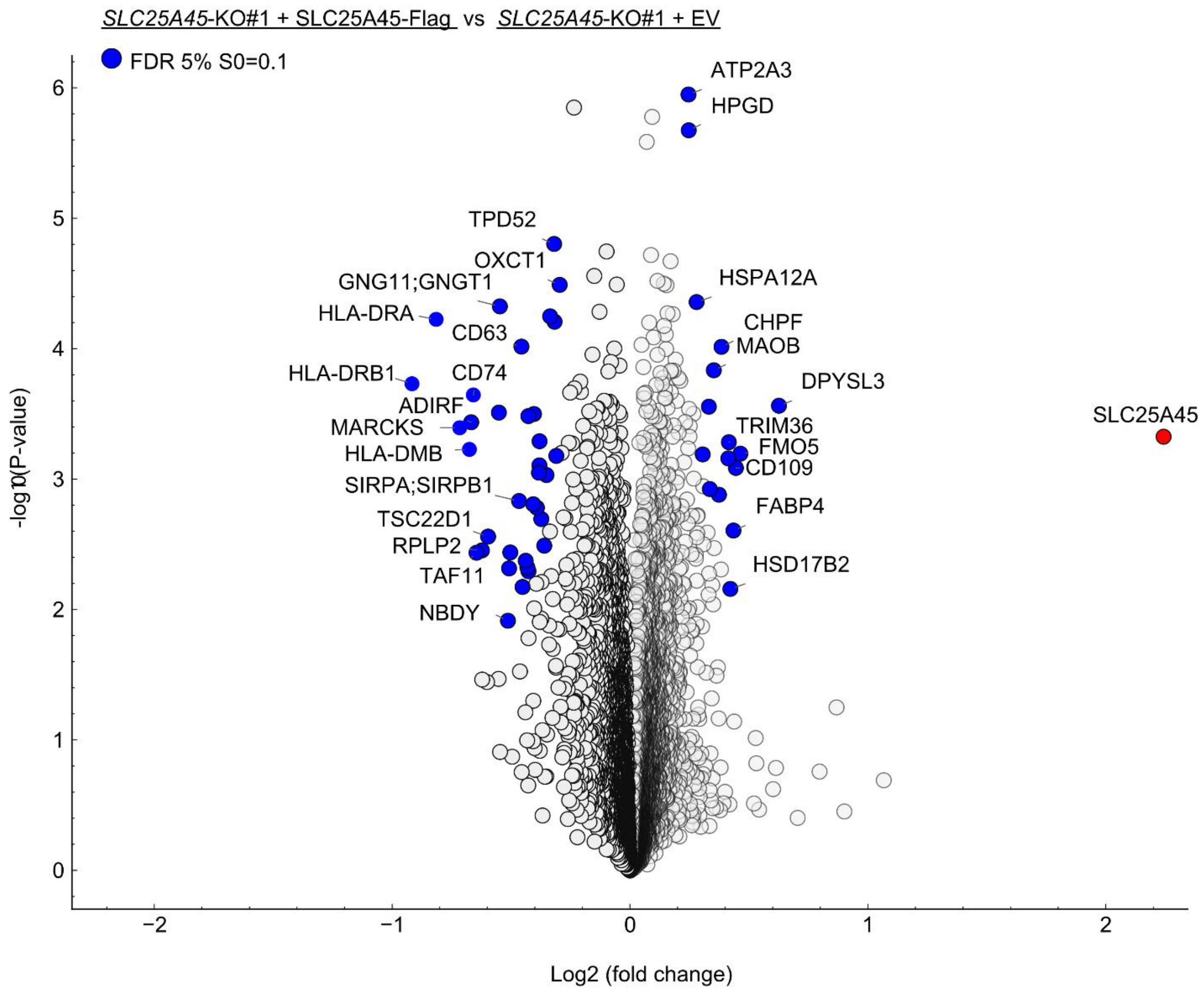

Figure S1. Characterisation of SLC25A45 depleted AsPC-1 cells. Related to Figure 1. (A) *SLC25A45* expression determined by RT-qPCR in the indicated cell lines treated with Scr or *SLC25A45* siRNA for 72 h (n=2). HPNE are hTERT-immortalized epithelial cells from a normal pancreas. AsPC-1 and PANC-1 are pancreatic cancer cell lines. **(B)** Inference of CRISPR Edits (ICE) analysis of *SLC25A45* KO AsPC-1 monoclones generated by CRISPR-Cas9 (https://ice.synthego.com). **(C)** Volcano plot of log2 fold change in protein abundance in *SLC25A45* KO + SLC25A45-FLAG AsPC-1 cells compared with *SLC25A45* KO + EV (empty vector) AsPC-1 cells as measured by LC-MS (n=3; FDR, false discovery rate). Data represent means ± SD; n=independent cultures.

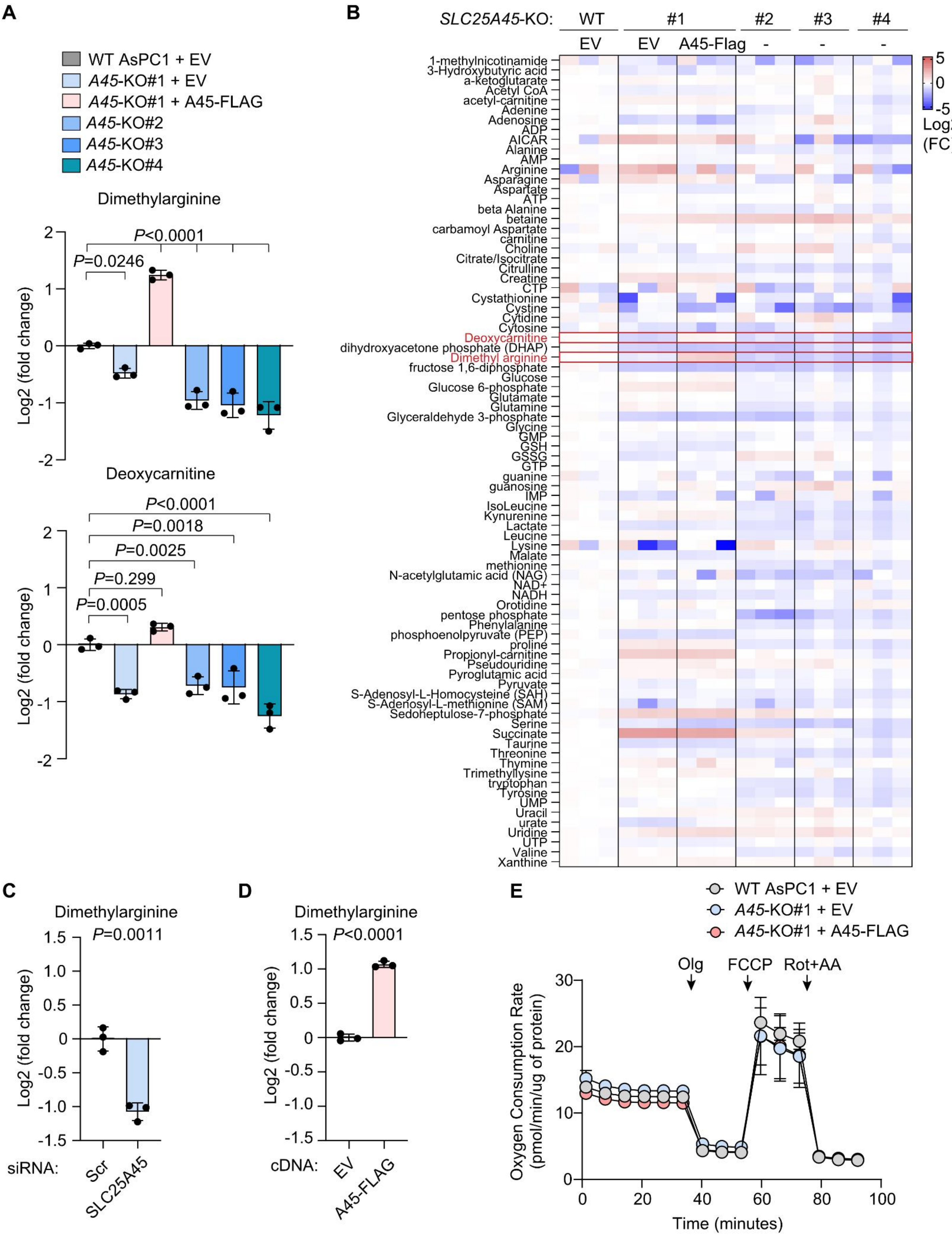
A
WT AsPC1 + EV
A45-KO#1 + EV
A45-KO#1 + A45-FLAG
A45-KO#2
A45-KO#3
A45-KO#4
Dimethylarginine
P<0.0001
P=0.0246
Log2 (fold change)
Deoxycarnitine
P<0.0001
P=0.0018
P=0.0025
P=0.299
P=0.0005
B
SLC25A45-KO:
WT
#1
#2
#3
#4
EV
EV
A45-Flag
-
-
-
Log2 (FC)
1-methylnicotinamide
3-Hydroxybutyric acid
a-ketoglutarate
Acetyl CoA
acetyl-carnitine
Adenine
Adenosine
ADP
AICAR
Alanine
AMP
Arginine
Asparagine
Aspartate
ATP
beta Alanine
betaine
carbamoyl Aspartate
carnitine
Choline
Citrate/Isocitrate
Citrulline
Creatine
CTP
Cystathionine
Cystine
Cytidine
Cytosine
Deoxycarnitine
dihydroxyacetone phosphate (DHAP)
Dimethyl arginine
fructose 1,6-diphosphate
Glucose
Glucose 6-phosphate
Glutamate
Glutamine
Glyceraldehyde 3-phosphate
Glycine
GMP
GSH
GSSG
GTP
guanine
guanosine
IMP
IsoLeucine
Kynurenine
Lactate
Leucine
Lysine
Malate
methionine
N-acetylglutamic acid (NAG)
NAD+
NADH
Orotidine
pentose phosphate
Phenylalanine
phosphoenolpyruvate (PEP)
proline
Propionyl-carnitine
Pseudouridine
Pyroglutamic acid
Pyruvate
S-Adenosyl-L-Homocysteine (SAH)
S-Adenosyl-L-methionine (SAM)
Sedoheptulose-7-phosphate
Serine
Succinate
Taurine
Threonine
Thymine
Trimethyllysine
tryptophan
Tyrosine
UMP
Uracil
urate
Uridine
UTP
Valine
Xanthine
C
Dimethylarginine
P=0.0011
Log2 (fold change)
siRNA:
Scr
SLC25A45
D
Dimethylarginine
P<0.0001
Log2 (fold change)
cDNA:
EV
A45-FLAG
E
WT AsPC1 + EV
A45-KO#1 + EV
A45-KO#1 + A45-FLAG
Olg
FCCP
Rot+AA
Oxygen Consumption Rate (pmol/min/ug of protein)
Time (minutes)

Figure S2. SLC25A45 loss and overexpression alters dimethylarginine levels in AsPC-1 cells. Related to Figure 1. (A) Log2 fold change in dimethylarginine and deoxycarnitine levels in the indicated AsPC-1 *SLC25A45* knockout (*A45*-KO) clones compared with WT AsPC-1 + EV (empty vector) (n=3). **(B)** Heatmap representation of log2 fold changes in polar metabolites from LC-MS analysis of the indicated AsPC-1 *SLC25A45* knockout (*A45*-KO) clones compared with WT AsPC-1 + EV. Each column represents one experiment. Deoxycarnitine and dimethylarginine are highlighted and these data are also presented in A. **(C)** Log2 fold change in dimethylarginine levels in AsPC-1 cells treated with Scr or *SLC25A45* siRNA for 72 h (n= 3). **(D)** Log2 fold change in dimethylarginine levels in WT AsPC-1 cells expressing EV (empty vector) or SLC25A45-FLAG (n=3). **(E)** Oxygen consumption rates in the indicated cell lines measured by Seahorse extracellular flux analysis and mitochondrial stress test. Wells were injected with oligomycin (Olg), FCCP, rotenone and antimycin A (Rot+AA) at the indicated timepoints (n=2). Data represent means ± SD; n=independent cultures (A-D) or independent experiments (E). *P*-values calculated using one-way ANOVA with Tukey’s multiple comparison test (A) or two-tailed unpaired t-test (C,D).

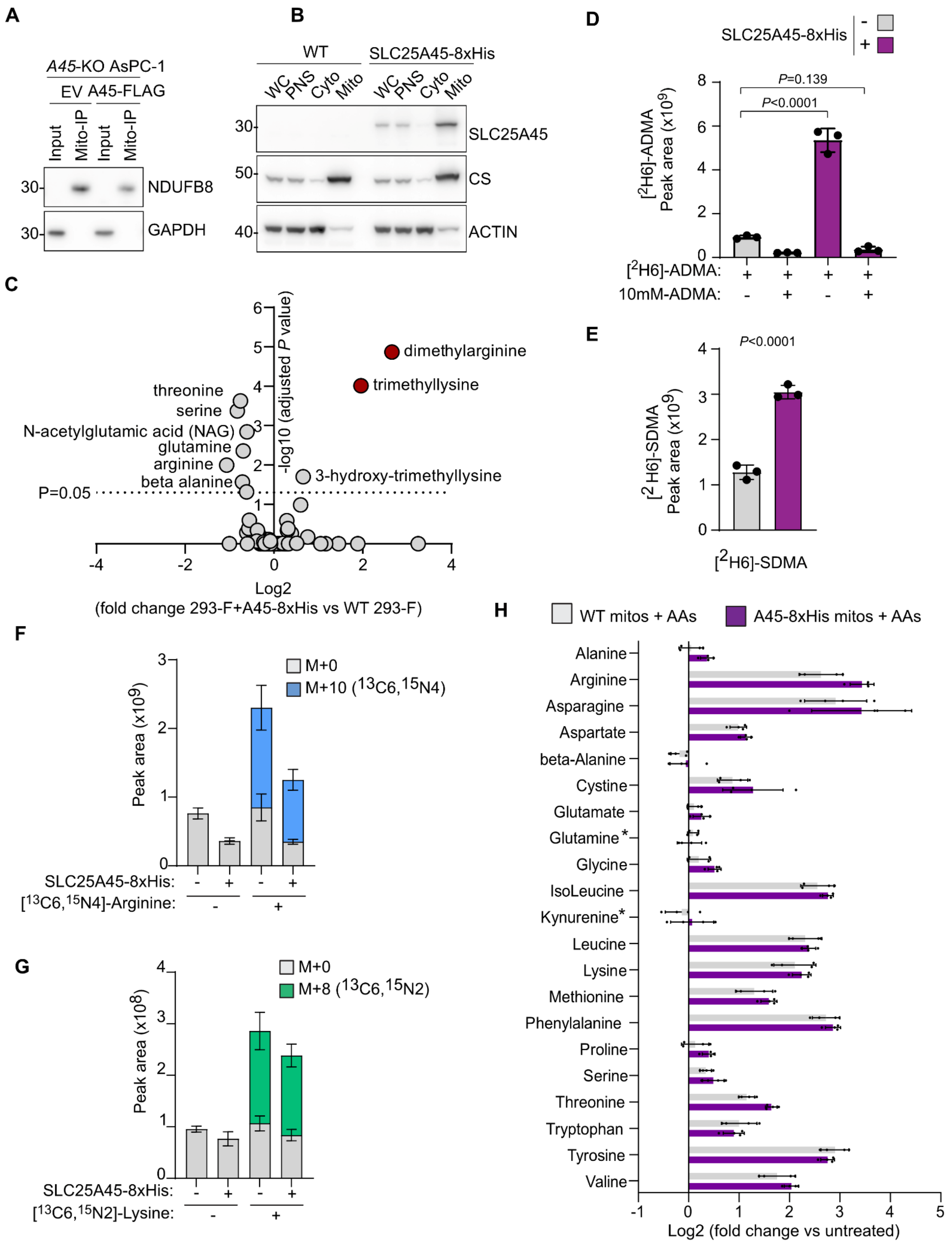

A
A45-KO AsPC-1
EV A45-FLAG
Input
Mito-IP
Input
Mito-IP
NDUFB8
GAPDH
B
WT
SLC25A45-8xHis
WC
PNS
Cyto
Mito
SLC25A45
CS
ACTIN
D
SLC25A45-8xHis
[2H6]-ADMA Peak area (x10^9)
P=0.139
P<0.0001
[2H6]-ADMA:
10mM-ADMA:
C
dimethylarginine
trimethyllysine
threonine
serine
N-acetylglutamic acid (NAG)
glutamine
arginine
beta alanine
3-hydroxy-trimethyllysine
P=0.05
-log10 (adjusted P value)
Log2
(fold change 293-F+A45-8xHis vs WT 293-F)
E
P<0.0001
[2H6]-SDMA Peak area (x10^9)
[2H6]-SDMA
F
M+0
M+10 (13C6,15N4)
Peak area (x10^9)
SLC25A45-8xHis:
[13C6,15N4]-Arginine:
G
M+0
M+8 (13C6,15N2)
Peak area (x10^8)
SLC25A45-8xHis:
[13C6,15N2]-Lysine:
H
WT mitos + AAs
A45-8xHis mitos + AAs
Alanine
Arginine
Asparagine
Aspartate
beta-Alanine
Cystine
Glutamate
Glutamine*
Glycine
IsoLeucine
Kynurenine*
Leucine
Lysine
Methionine
Phenylalanine
Proline
Serine
Threonine
Tryptophan
Tyrosine
Valine
Log2 (fold change vs untreated)

Figure S3. Analysis of amino acid uptake in isolated mitochondria. Related to Figure 1. (A) Representative immunoblot of mitochondria isolated by immunoprecipitation from *SLC25A45*-KO + EV (empty vector) and *SLC25A45*-KO + SLC25A45 FLAG AsPC-1 cells expressing 3XHA-EGFP-OMP25 used for metabolomic analysis in Figure 1B. Input = whole-cell lysate. NDUFB8 (NADH:ubiquinone oxidoreductase subunit B8) was used as a mitochondrial marker and GAPDH as a cytosolic marker. **(B)** Representative immunoblot of mitochondria isolated by differential centrifugation from wildtype (WT) and SLC25A45-8xHis expressing FreeStyle 293-F cells used for metabolite uptake assays in C-H and Figure 1C,D. WC, whole cell; PNS, post-nuclear supernatant; Cyto, cytosolic fraction; Mito, mitochondria. **(C)** Volcano plot of log2 fold change in mitochondrial metabolites against -log P values isolated from FreeStyle 293-F cells + SLC25A45-8xHis compared with WT FreeStyle 293-F cells (n=4). **(D)** Mitochondrial [^{2}H6]-asymmetric dimethylarginine (ADMA) levels after treatment of mitochondria isolated from the indicated FreeStyle293-F cells with 10 μM [^{2}H6]-ADMA +/- 10 mM unlabelled ADMA for 10 min (n= 3). **(E)** Mitochondrial [^{2}H6]-symmetric dimethylarginine (SDMA) levels after treatment of mitochondria isolated from the indicated FreeStyle293-F cells with 10 μM [^{2}H6]-SDMA for 10 min (n= 3). **(F)** Abundance and mass isotopologue distribution of arginine in mitochondria isolated from the indicated FreeStyle293-F cells and treated with 150 μM [^{13}C6,^{15}N4]-arginine for 10 min (n=4). **(G)** Abundance and mass isotopologue distribution of lysine in mitochondria isolated from the indicated FreeStyle293-F cells and treated with 150 μM [^{13}C6,^{15}N2]-lysine for 10 min (n=4) **(H)** Amino acid levels in mitochondria isolated from the indicated FreeStyle293-F cells treated with amino acid mix for 10 min (see Table S5 for amino acid mix composition; * = amino acids not included in the amino acid mix). Data presented as log2 fold change compared to untreated mitochondria isolated from each cell line. All statistical comparisons returned P>0.05 (n=4). Data represent means ± SD; n=independent mitochondrial preparations. *P* values were calculated using multiple two-tailed unpaired t-tests with Holm-Šídák multiple comparison correction (C and H), two-way ANOVA with Tukey's multiple comparison test (D) or two-tailed unpaired t-test (E).

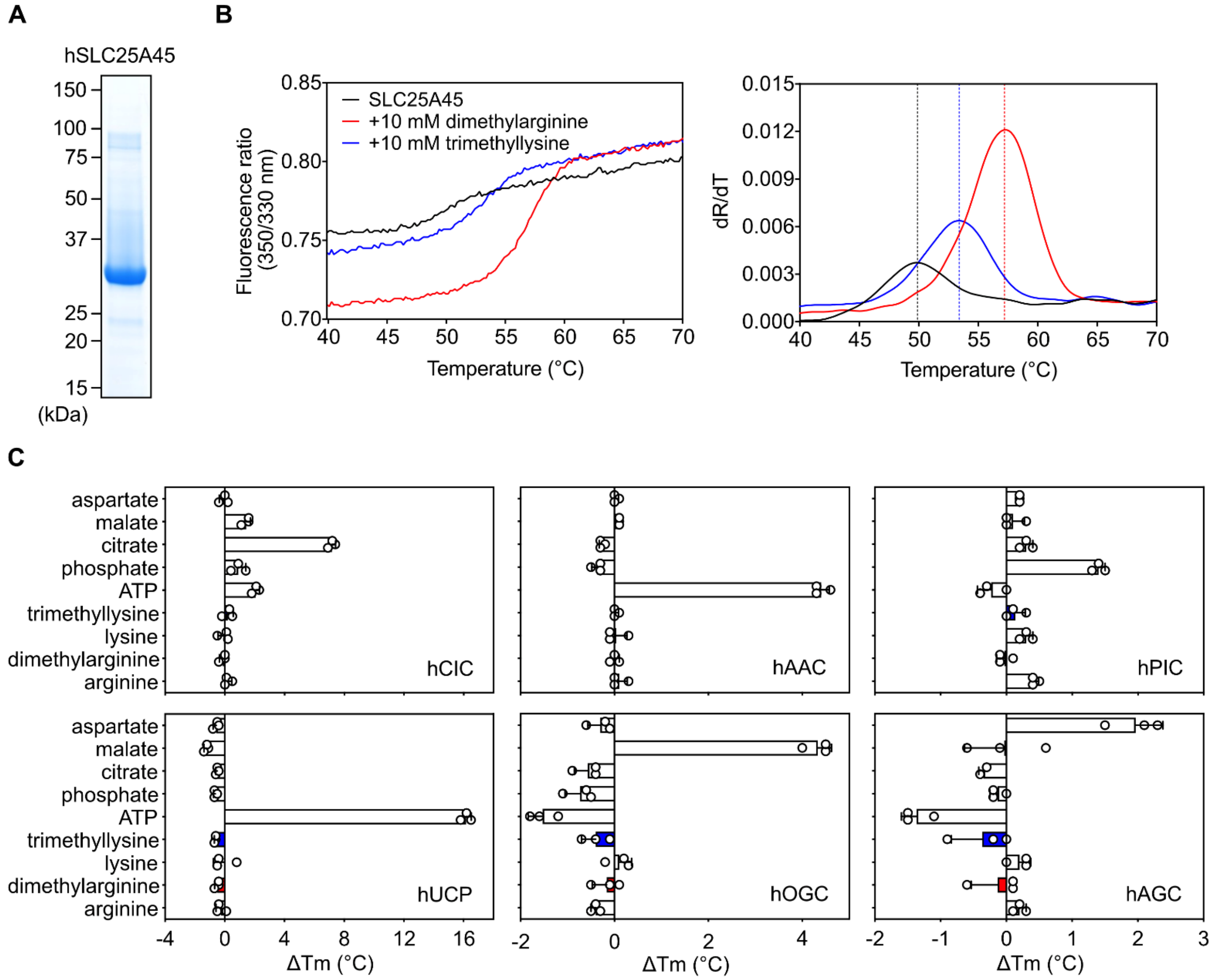

Figure S4. Thermostability shift assays with purified SLC25 proteins. Related to Figure 1. (A) Instant-blue stained SDS-PAGE gel of purified human SLC25A45. **(B)** Representative unfolding curves of ~10 µg protein without compound (black trace), or with 10 mM dimethylarginine (red trace) or 10 mM trimethyllysine (blue trace). The apparent melting temperatures (Tm), indicated by the dashed line, correspond to the peak in the derivative of the unfolding curve (dR/dT). **(C)** The change in melting temperature (ΔTm) of human citrate carrier (hCIC, SLC25A1), human ADP/ATP carrier (hAAC, SLC25A4), human phosphate carrier (hPIC, SLC25A3), human uncoupling protein (hUCP, SLC25A7), human oxoglutarate carrier (hOGC, SLC25A11) and human aspartate/glutamate carrier (hAGC, SLC25A13) in the presence of 10 mM compound, as indicated. Data represent means ± SD, n= 3 technical replicates.

S/R/R - potential contact points

GXXXG motif
Proline kink
Matrix gate (Matrix network + Gln brace)
Cardiolipin binding
Cytoplasmic gate (πxxxπ motif + Tyr brace + cytoplasmic network)

```
sp|Q8N413|S2545_HUMAN      MPVEEFVAGWISGALGLVLGHPFDTVKVRLQTQTTYRGIVDCMVKIYRHESLLGFFKGMS     60
sp|Q8CFJ7|S2545_MOUSE      MPVEEFVAGWISGAVGLVLGHPFDTVKVRLQTQSTYQGIVDCVVKTYRHESVLGFFKGMS     60
tr|D3ZL04|D3ZL04_RAT       MPVEEFVAGWISGAVGLVLGHPFDTVKVRLQTQNTYQGIVDCVVKTYRHESVLGFFKGMS     60
                           **************:******************.**:*****:** *****:********

sp|Q8N413|S2545_HUMAN      FPIASIAVVNSVLFGVYSNTLLVLTATSHQERRAQPPSYMHIFLAGCTGGFLQAYCLAPF     120
sp|Q8CFJ7|S2545_MOUSE      FPIASVALVNSVLFGVYSNTLLALTATSHQERRAQPPSYTNIFIAGCTGGLLQAYCLAPF     120
tr|D3ZL04|D3ZL04_RAT       FPIASVALVNSVLFGVYSNTLLALTATSHQERRAQPPSYTNIFIAGCTGGLLQAYCLAPF     120
                           *****:*:**************.**************** :**:******:*********

sp|Q8N413|S2545_HUMAN      DLIKVRLQNQTEPRAQPGSPPPRYQGPVHCAASIFREEGPRGLFRGAWALTLRDTPTVGI     180
sp|Q8CFJ7|S2545_MOUSE      DLIKVRLQNQTEPRMQISSSMPRYRGPVHCAASILREEGPQGLFRGSWALVLRDTPTLGM     180
tr|D3ZL04|D3ZL04_RAT       DLIKVRLQNQTEPRMQIGSSTPRYRGPVHCAASILKEEGPQGLFRGSWALVLRDTPTLGM     180
                           ************** * .*  ***:*********::****:*****:***.******:*:

sp|Q8N413|S2545_HUMAN      YFITYEGLCRQYTPEGQNPSSATVLVAGGFAGIASWVAATPLDMIKSRMQMDGLRRRVYQ     240
sp|Q8CFJ7|S2545_MOUSE      YFVTYEGLCRQYTPEGQNPSSATVLVAGGFAGIASWITATPFDVIKSRMQMDGLKGRKYG     240
tr|D3ZL04|D3ZL04_RAT       YFVTYEGLCRQYTPEGQNPSSATVLVAGGFAGIASWITATPFDVIKSRMQMDGLKGRKYG     240
                           **:*********************************::***:*:**********: * *

sp|Q8N413|S2545_HUMAN      GMLDCMVSSIRQEGLGVFFRGVTINSARAFPVNAVTFLSYEYLLRWWG   288
sp|Q8CFJ7|S2545_MOUSE      GMLDCMASSFRQEGIGVFFKGMTLNSARAFPVNAATFLSYEYLLRLWR   288
tr|D3ZL04|D3ZL04_RAT       GMLDCMASSFRQEGIGVFFKGMTLNSARAFPVNAATFLSYEYLLRLWR   288
                           ******.**:****:****:*:*:**********.********** *
```

Arginine 285

Figure S5. SLC25A45 amino acid sequence. Related to Figure 2. Aligned amino acid sequences of human, mouse (*Mus musculus*) and rat (*Rattus norvegicus*) SLC25A45 generated with Clustal Omega Multiple Sequence Alignment (https://www.ebi.ac.uk/jdispatcher/msa/clustalo). Conserved SLC25 functional motifs are highlighted.

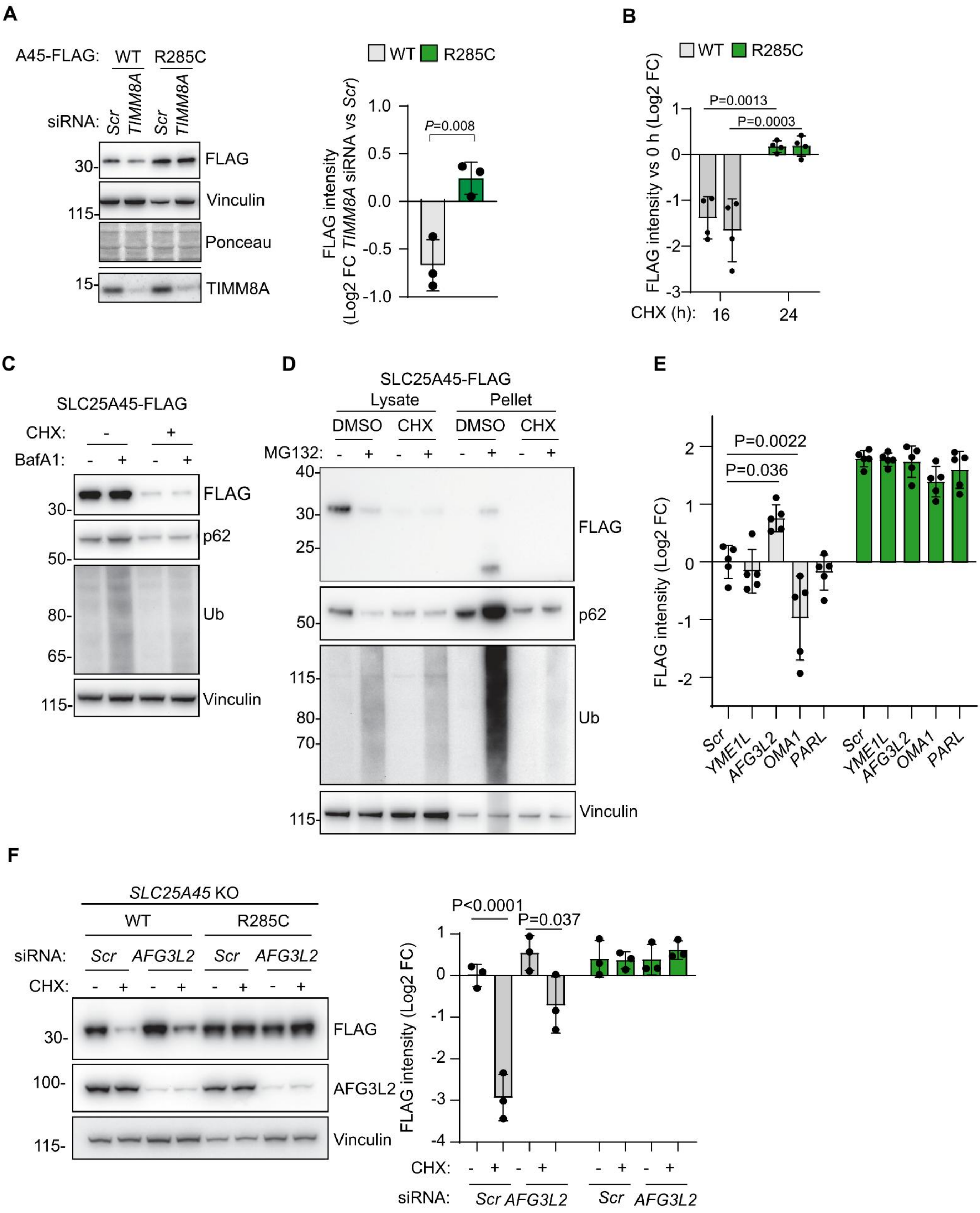
A
A45-FLAG:
WT
R285C
siRNA:
Scr
TIMM8A
Scr
TIMM8A
30-
FLAG
Vinculin
115-
Ponceau
15-
TIMM8A
WT
R285C
FLAG intensity
(Log2 FC TIMM8A siRNA vs Scr)
P=0.008
1.0
0.5
0.0
-0.5
-1.0
B
WT
R285C
FLAG intensity vs 0 h (Log2 FC)
P=0.0013
P=0.0003
1
0
-1
-2
-3
CHX (h):
16
24
C
SLC25A45-FLAG
CHX:
-
+
BafA1:
-
+
-
+
FLAG
30-
p62
50-
Ub
80-
65-
Vinculin
115-
D
SLC25A45-FLAG
Lysate
Pellet
DMSO
CHX
DMSO
CHX
MG132:
-
+
-
+
-
+
-
+
40-
30-
25-
FLAG
p62
50-
115-
80-
70-
Ub
Vinculin
115-
E
FLAG intensity (Log2 FC)
P=0.0022
P=0.036
2
1
0
-1
-2
Scr
YME1L
AFG3L2
OMA1
PARL
Scr
YME1L
AFG3L2
OMA1
PARL
F
SLC25A45 KO
WT
R285C
siRNA:
Scr
AFG3L2
Scr
AFG3L2
CHX:
-
+
-
+
-
+
-
+
30-
FLAG
100-
AFG3L2
115-
Vinculin
FLAG intensity (Log2 FC)
P<0.0001
P=0.037
1
0
-1
-2
-3
-4
CHX:
-
+
-
+
-
+
-
+
siRNA:
Scr
AFG3L2
Scr
AFG3L2

Figure S6. Stabilisation of SLC25A45 by arginine 268 to cysteine mutation. Related to Figure 2. (A) Immunoblot analysis of *SLC25A45* KO AsPC-1 cells expressing SLC25A45^{WT}-FLAG or SLC25A45^{R285C}-FLAG treated with scrambled (Scr) or *TIMM8A* siRNA for 72 h. Log2 fold change in intensity of FLAG bands are quantified (n=3). **(B)** Log2 fold change in intensity of FLAG bands after CHX treatment. Quantification of data presented in Fig. 2D (n=4). **(C)** Immunoblot analysis of *SLC25A45*-KO + SLC25A45-FLAG AsPC-1 cells treated with 150 µg/mL cycloheximide (CHX) and 200 µM bafilomycin A1 (BafA1) for 16 h (n=1) **(D)** Immunoblot analysis of lysate and pellet fractions from *SLC25A45*-KO + SLC25A45-FLAG AsPC-1 cells treated with 150 µg/mL CHX and 10 µM MG132 for 16 h (n=1) **(E)** Log2 fold change in intensity of FLAG bands compared to scrambled (Scr) siRNA-treated SLC25A45^{WT}-FLAG expressing cells. Quantification of data presented in Fig. 2E. (n=5). **(F)** Immunoblot analysis of *SLC25A45* KO AsPC-1 cells expressing SLC25A45^{WT}-FLAG or SLC25A45^{R285C}-FLAG treated with Scr or *AFG3L2* siRNA for 72 h followed by 24 h CHX. Log2 fold change in intensity of FLAG bands compared to DMSO treated Scr siRNA-treated SLC25A45^{WT}-FLAG expressing cells are quantified (n=3). Data represent means ± SD, n= independent cultures. *P* values calculated using two-tailed unpaired t-test (A) or two-way ANOVA with Tukey's multiple comparison test (B, E, F).

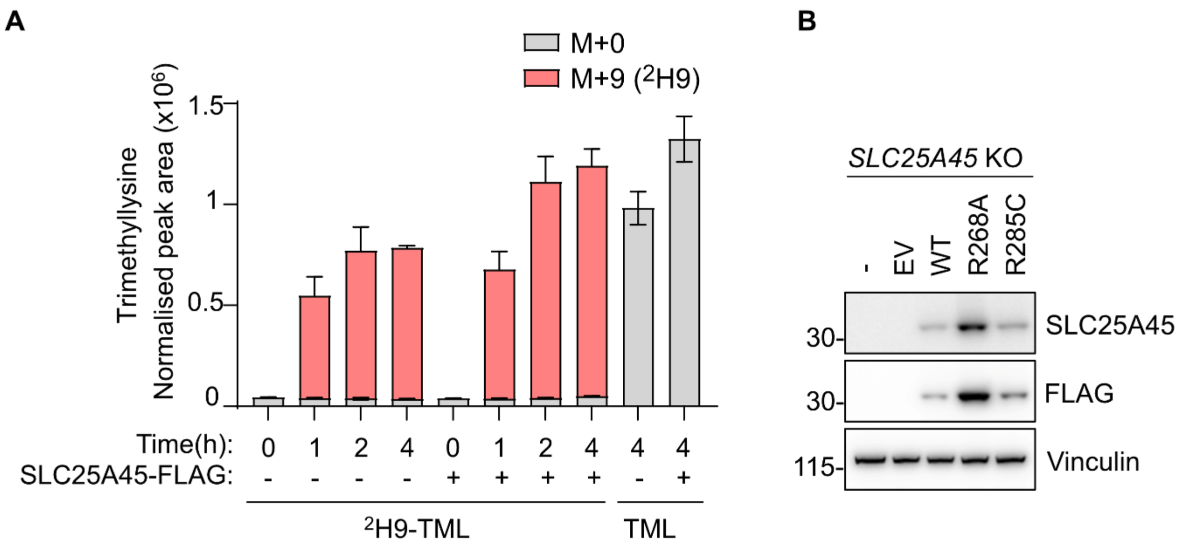

Figure S7. Trimethyllysine labelling of AsPC-1 cells and analysis of SLC25A45 mutants. Related to Figure 3. (A) Normalised abundance and mass isotopologue distribution of trimethyllysine (TML) in *SLC25A45* KO AsPC-1 cells expressing EV or SLC25A45-FLAG treated with 100 µM unlabelled TML or 100 µM [^{2}H9]-TML for the indicated times (n=3 independent cultures). **(B)** Immunoblot analysis of *SLC25A45*-KO AsPC-1 cells transduced with the indicated variants of SLC25A45-FLAG. EV, empty vector. Data represent means ± SD.

A

ICE Analysis of OVCAR-3 and Kuramochi *SLC25A45* KO clones

Clone	Guide Target	PAM sequence	Indel %	Model Fit (R^2)	Knockout-Score
OV-#1	GATGAAGTAGATCCCCACCG	TGG	96	0.96	96
OV-#2	GATGAAGTAGATCCCCACCG	TGG	97	0.97	97
KUR-#1	GATGAAGTAGATCCCCACCG	TGG	90	0.91	90
KUR-#2	GATGAAGTAGATCCCCACCG	TGG	91	0.91	91

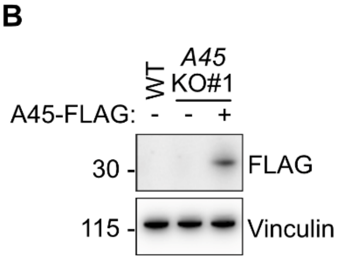

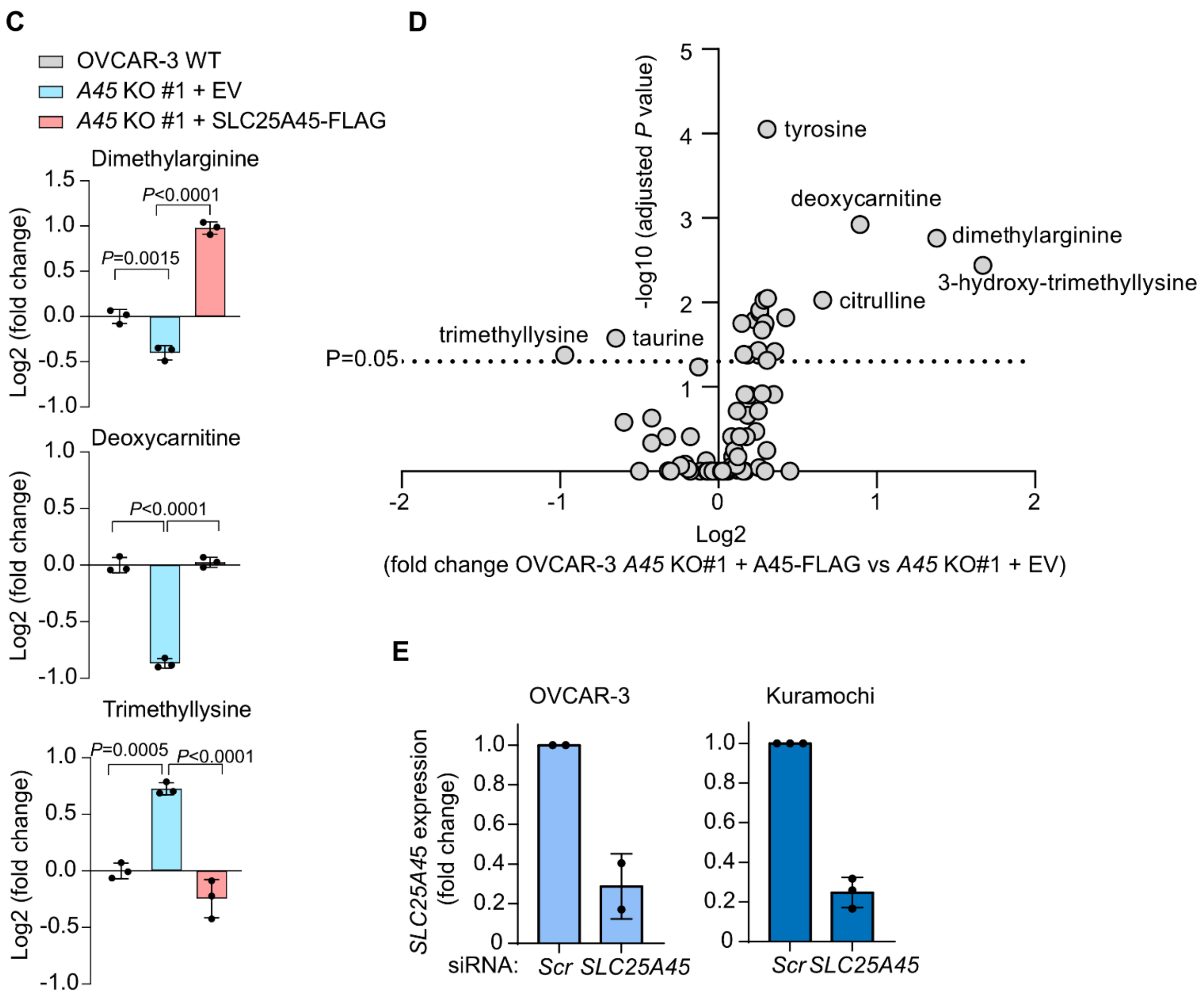

Figure S8. Metabolic analysis of SLC25A45-depleted ovarian cancer cell lines. Related to Figure 4. **(A)** Inference of CRISPR Edits (ICE) analysis of *SLC25A45* KO OVCAR-3 (OV) and Kuramochi (KUR) cells monoclones generated by CRISPR-Cas9 (https://ice.synthego.com). **(B)** Representative immunoblot of indicated OVCAR-3 cells used in C and D. **(C)** Log2 fold change of indicated metabolites in *SLC25A45* knockout + empty vector (*A45*-KO #1 + EV) OVCAR-3 cells and those complemented with SLC25A45-FLAG (*A45*-KO #1 + SLC25A45-FLAG) compared with WT OVCAR-3 (n=3). **(D)** Volcano plot of log2 fold change in whole-cell polar metabolites against -log P values from *SLC25A45*-KO #1 + SLC25A45-FLAG OVCAR-3 cells compared with *SLC25A45*-KO #1 + EV cells. (n=3). **(E)** *SLC25A45* expression determined by RT-qPCR in the indicated cell lines treated with Scr or *SLC25A45* siRNA for 72 h (n=2 for OVCAR-3 and n=3 for Kuramochi). Data represent means ± SD, n=independent cultures. *P* values were calculated using one-way ANOVA with Tukey's multiple comparison test (C) or multiple two-tailed unpaired t-tests with Holm-Šídák multiple comparison correction (D).

Cloning primers			
Gene	Forward primer		Reverse primer
SLC25A45-Flag	TCTAGAATTCATGCCGGTGGAGGA ATTT		CTAGGATCCTCACTTATCGTCGTCA TCCTT
BBOX1-Flag	GAGGATCTATTTCCGGTGGCCACC ATGGCTTGTACCAT		GAGGGAGAGGGGCGGTCAGTTTC CATTCTCCACCCTCTGC
Site-directed mutagenesis			
Gene	Forward primer		Reverse primer
SLC25A45 R268A	CACCATCAACAGTGCCGCCGCCTT TCCCGTCAATGCTG		CAGCATTGACGGGAAAGGCGGCG GCACTGTTGATGGTG
SLC25A45 R285C	TATCTCCTCTGCTGGTGGGGAG		CTCCCCACCAGCAGAGGAGATA
qPCR primers			
Gene	Forward primer		Reverse primer
ACTB	GACGACATGGAGAAAATCTG		ATGATCTGGGTCATCTTCTC
SLC25A45	GTTTGACACTGTAAAGGTGAG		CTATAGACCCCAAACAGGAC
siRNA pools			
Target	Source		Identifier
Scramble	Dharmacon		D-001206-13-05
AFG3L2	Dharmacon		M-005781-00-0005
OMA1	Dharmacon		M-006103-01-0005
PARL	Dharmacon		M-005781-00-0005
SLC25A45	Dharmacon		M-007351-01-0005
TIMM8A	Dharmacon		M-010342-01-0005
YME1L	Dharmacon		M-006103-01-0005
CRISPR gRNA			
Plasmid	Source	Identifier	Sequence
pLentiCRISPR v2 SLC25A45 gRNA#1	GenSript	U1421HF150	GATGAAGTAGATCCCCACCG

Table S4. Oligonucleotide sequences. Related to STAR Methods.

	**Amino acid**	**Final concentration (μM)**
**1X MEM Non-Essential Amino Acids**	Glycine	100
	L-Alanine	100
	L-Asparagine	100
	L-Aspartic acid	100
	L-Glutamic acid	100
	L-Proline	100
	L-Serine	100
**1X MEM Amino Acids**	L-Arginine hydrochloride	600
	L-Cystine	100
	L-Histidine hydrochloride	200
	L-Isoleucine	400
	L-Leucine	400
	L-lysine hydrochloride	397
	L-Methionine	101
	L-Phenylalanine	200
	L-Threonine	400
	L-Tryptophan	50
	L-Tyrosine	199
	L-Valine	400

Table S5. Amino acid mix. Related to Figure S3H and STAR Methods.
