# Supplementary material for: SLC25A45 is required for mitochondrial uptake of methylated amino acids and de novo carnitine biosynthesis
Source: Mol Cell. 2025 Nov 6;85(21):4093–4104.e8. doi: 10.1016/j.molcel.2025.08.018 (PMC12594208; doi:10.1016/j.molcel.2025.08.018)
Supplement: Document S2. Article plus supplemental information [file mmc5.pdf]

Molecular Cell

Short article

SLC25A45 is required for mitochondrial uptake of methylated amino acids and *de novo* carnitine biosynthesis

Graphical abstract

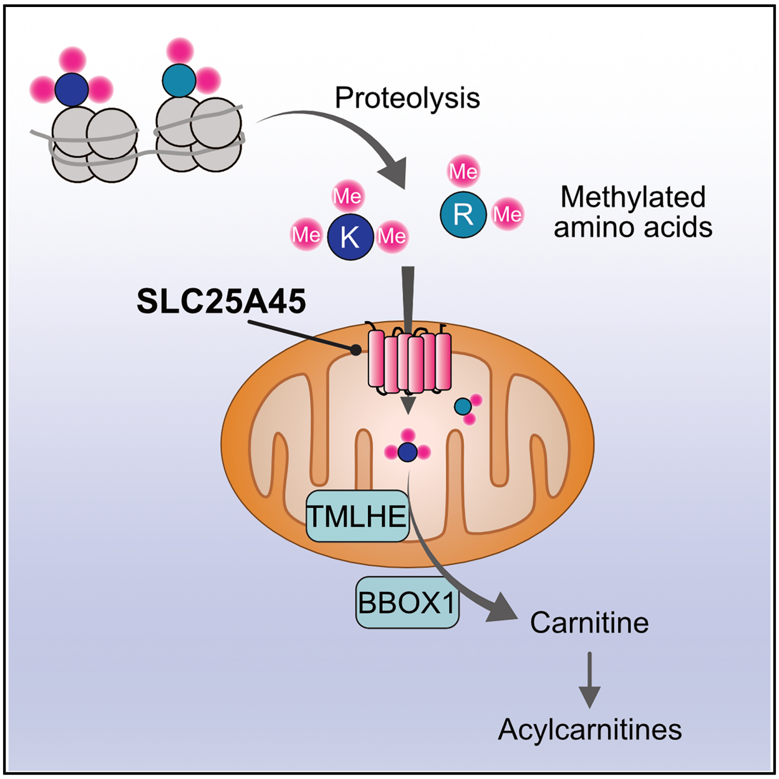

Highlights

- Mitochondrial import of methylated amino acids depends on SLC25A45
- *SLC25A45* mutation (*R285C*) associates with altered levels of methylated amino acids in humans
- SLC25A45^{R285C} is stabilized within mitochondria
- SLC25A45 drives carnitine biosynthesis from trimethyllysine

Authors

Marilia M. Dias, Martin S. King, Engy Shokry, ..., David Sumpton, Edmund R.S. Kunji, Thomas MacVicar

Correspondence

thomas.macvicar@glasgow.ac.uk

In brief

Dias et al. discover that the orphan solute carrier protein, SLC25A45, enables mitochondrial import of methylated amino acids. SLC25A45-dependent mitochondrial uptake of trimethyllysine drives carnitine biosynthesis. Mutation near its C terminus impacts SLC25A45 proteostasis and associates with altered methylated amino acid levels in human plasma.

Dias et al., 2025, Molecular Cell *85*, 4093–4104
November 6, 2025 © 2025 The Authors. Published by Elsevier Inc.
https://doi.org/10.1016/j.molcel.2025.08.018

Molecular Cell

CellPress
OPEN ACCESS

Short article

SLC25A45 is required for mitochondrial uptake of methylated amino acids and *de novo* carnitine biosynthesis

Marilia M. Dias,[1] Martin S. King,[2] Engy Shokry,[1] Sergio Lilla,[1] Nikki Paul,[1] Peter Thomason,[1] Sara Zanivan,[1,3,4] David Sumpton,[1,3] Edmund R.S. Kunji,[2] and Thomas MacVicar[1,3,5,*]

[1]Cancer Research UK Scotland Institute, Glasgow G61 1BD, UK
[2]MRC Mitochondrial Biology Unit, University of Cambridge, The Keith Peters Building, Hills Road, Cambridge CB2 0XY, UK
[3]School of Cancer Sciences, University of Glasgow, Glasgow G61 1BD, UK
[4]Present address: Department of Experimental Therapeutics, University of Texas MD Anderson Cancer Center, Houston, TX 77030, USA
[5]Lead contact
*Correspondence: thomas.macvicar@glasgow.ac.uk
https://doi.org/10.1016/j.molcel.2025.08.018

SUMMARY

Methylated amino acids accumulate upon the degradation of methylated proteins and are implicated in diverse metabolic and signaling pathways. Disturbed methylated amino acid homeostasis is associated with cardiovascular disease and renal failure. Mitochondria are core processing hubs in conventional amino acid metabolism, but how they interact with methylated amino acids is unclear. Here, we reveal that the orphan mitochondrial solute carrier 25A45 (SLC25A45) is required for the mitochondrial uptake of methylated amino acids. SLC25A45 binds with dimethylarginine and trimethyllysine but has no affinity for unmethylated arginine and lysine. A non-synonymous mutation of human *SLC25A45* (*R285C*) stabilizes the carrier by limiting its proteolytic degradation and associates with altered methylated amino acids in human plasma. Metabolic tracing of trimethyllysine in cancer cells demonstrates that SLC25A45 drives the biosynthesis of the key amino acid derivative, carnitine. SLC25A45 is therefore an essential mediator of compartmentalized methylated amino acid metabolism.

INTRODUCTION

Eukaryotic cell metabolism depends on the regulated exchange of metabolites between the mitochondria and cytosol.[1] While the outer mitochondrial membrane is porous to small molecules, metabolites must cross the impermeable inner mitochondrial membrane (IMM) via dedicated transporter proteins.[2,3] Mitochondrial metabolite transporters have emerged as key regulatory nodes in numerous compartmentalized metabolic pathways associated with metabolic disease and cancer.[4,5]

Most mitochondrial metabolite transporters belong to solute carrier (SLC) family 25, which is the largest SLC family in humans.[3] Conventional SLC25 carriers contain six transmembrane α-helices arranged in 3-fold pseudosymmetry,[6–9] which undergo conserved conformational changes to alternate between cytoplasmic-open and matrix-open states.[8–12] Substrate binding depends on amino acid residues in the central cavity, and transporter function also requires other conserved functional elements shared by SLC25 carriers, such as salt bridge networks, braces, and cardiolipin-binding sites.[8,9,12–14] However, approximately one-quarter of mitochondrial SLC25 family members remain orphan transporters with unknown substrates. De-orphanization of mitochondrial transporters can provide new opportunities to target specific metabolic pathways in pathophysiology.[15]

Intracellular pools of methylated amino acids (MeAAs) consist predominantly of methylated lysine and arginine derived from the breakdown of methylated proteins, including histones.[16,17] MeAAs play diverse roles in downstream metabolic and signaling pathways, which may require entry into mitochondria via unknown transporter(s). For instance, liver and kidney mitochondria metabolize dimethylarginine, which is an endogenous regulator of nitric oxide signaling implicated in cardiovascular and renal disease.[18,19] Meanwhile, trimethyllysine is imported by mitochondria for the first step of carnitine biosynthesis.[20] Carnitine is conjugated to long-chain fatty acid species to build acylcarnitines prior to import into mitochondria and peroxisomes for β-oxidation.[21–23] Acylcarnitine levels are also implicated in lipotoxicity[24] and epigenetic signaling.[25,26]

Here, we demonstrate that the orphan metabolite transporter, SLC25A45, is required for the mitochondrial import of MeAAs and carnitine biosynthesis. Our work reveals that SLC25A45 is a key node in the regulation of compartmentalized MeAA metabolism.

This is an open access article under the CC BY license (http://creativecommons.org/licenses/by/4.0/).

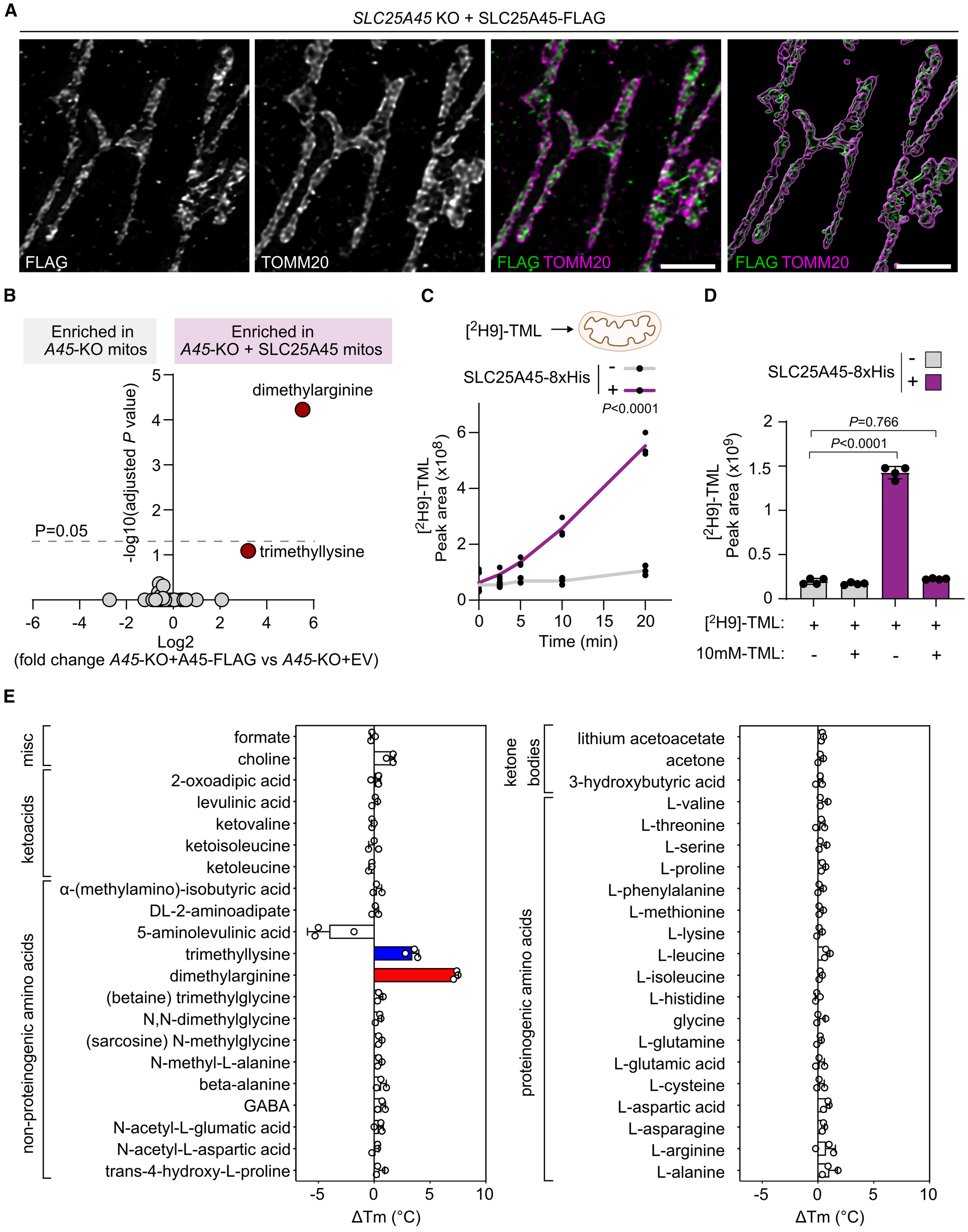

(legend on next page)

CellPress
OPEN ACCESS

RESULTS

SLC25A45 is required for the mitochondrial uptake of methylated amino acids

Prior phylogenetic analyses indicated that SLC25A45 is a mitochondrial amino acid transporter.[27,28] SLC25A45 shares the closest sequence homology with the liver-specific orphan transporter, SLC25A47[29,30]; the putative choline transporter, SLC25A48[31–33]; and the basic amino acid transporter, SLC25A29.[34] Mitochondrial localization of SLC25A45-FLAG was confirmed (Figure 1A). We chose to characterize the metabolic role of SLC25A45 in AsPC-1 cells because of its high level of expression when compared with other pancreatic cancer cell lines (Cancer Cell Line Encyclopedia [CCLE]; Figure S1A). Four *SLC25A45* knockout (KO) clones were generated (Figure S1B), and SLC25A45-FLAG was expressed in one of these clones. Proteomic analysis did not reveal significant differences in metabolite transporters or metabolic enzymes between KO and complemented cells other than SLC25A45 (Figure S1C; Table S1). To assess cellular metabolic regulation by SLC25A45, we analyzed the polar metabolome by liquid chromatography-mass spectrometry (LC-MS) and found a significant depletion of dimethylarginine and deoxycarnitine in cells lacking SLC25A45, whereas both metabolites were restored or elevated further upon re-expression of SLC25A45 (Figures S2A and S2B; Table S2). Consistently, dimethylarginine levels were reduced upon suppression of SLC25A45 by small interfering RNA (siRNA) (Figure S2C) and increased upon exogenous expression of SLC25A45 in wild-type (WT) AsPC-1 cells (Figure S2D). Loss of SLC25A45 did not impact other amino acids or citric acid cycle metabolites in a consistent manner (Figure S2B), and oxygen consumption rates were unaffected (Figure S2E).

To determine the impact of SLC25A45 on metabolites within mitochondria, we expressed 3xHA-EGFP-OMP25 Mito-Tag in *SLC25A45* KO cells and *SLC25A45* KO cells expressing SLC25A45-FLAG and immunopurified mitochondria prior to LC-MS[35] (Figures 1B and S3A; Table S2). Mitochondria expressing SLC25A45-FLAG contained a striking ∼56-fold increase in dimethylarginine (Figure 1B). Trimethyllysine was the only other enriched metabolite in mitochondria containing SLC25A45 (∼7-fold), although this was determined to be below the threshold of statistical significance (Figure 1B). Dimethylarginine and trimethyllysine were enriched significantly in mitochondria isolated from SLC25A45-8xHis-expressing FreeStyle 293-F cells, which do not express detectable levels of endogenous SLC25A45 protein (Figures S3B and S3C; Table S2).[36]

Next, we tested whether SLC25A45 influences the mitochondrial uptake of labeled MeAAs. Expression of SLC25A45-8xHis was essential for the uptake of deuterium-labeled trimethyllysine [^{2}H9]-TML by isolated mitochondria (Figure 1C). SLC25A45-dependent mitochondrial import of [^{2}H9]-TML was abolished by co-incubation with excess unlabeled trimethyllysine, which outcompetes [^{2}H9]-TML for access to the binding site (Figure 1D). SLC25A45 was also required for the mitochondrial uptake of the deuterium-labeled dimethylarginine isomers, asymmetric dimethylarginine ([^{2}H6]-ADMA) and symmetric dimethylarginine ([^{2}H6]-SDMA) (Figures S3D and S3E). Conversely, SLC25A45 expression did not enhance the mitochondrial uptake of unmethylated arginine or lysine (Figures S3F and S3G). Furthermore, the uptake of individual amino acids was comparable between WT and SLC25A45-8xHis-expressing mitochondria incubated with a mixture of amino acids (Figure S3H; Table S5). Together, these data demonstrate that SLC25A45 is necessary for the mitochondrial import of the methylated basic amino acids trimethyllysine and dimethylarginine.

Next, we purified human SLC25A45 from yeast mitochondria[37] (Figure S4A) and assessed protein folding by measuring thermostability using nano differential scanning fluorimetry (Figure S4B). This assay produces an apparent melting temperature, which corresponds to the temperature at which the rate of protein unfolding is highest.[38,39] The apparent melting temperature of SLC25A45 is 50.6°C ± 1.7°C (Figure S4B), which closely compares to the melting temperatures of other mitochondrial carrier proteins.[10,40–42] Membrane transport proteins, including mitochondrial carriers, are stabilized by substrates[11,14,38,40,42,43] and inhibitors,[42,44–46] resulting in increased apparent melting temperatures in thermostability assays. We found that trimethyllysine (ΔTm = 3.4°C) and dimethylarginine (ΔTm = 7.5°C) stabilize SLC25A45, whereas other metabolites, including lysine and arginine, had no effect (Figure 1E). Choline, which contains a trimethylamine group, stabilized SLC25A45 to a minor degree (ΔTm = 1.5°C) (Figure 1E). In parallel, we performed thermostability assays with other purified human SLC25 family proteins and observed substrate-dependent thermal shifts that are consistent with previous reports (Figure S4C).[10,11,40,44,45,47,48] Trimethyllysine and dimethylarginine did not alter the stability of the other SLC25 proteins (Figure S4C), which demonstrates that MeAAs are not generic interactors of mitochondrial carriers. Collectively, these data show that SLC25A45 binds methylated lysine and arginine specifically, in agreement with them being substrates of the transporter.

Dysregulated SLC25A45 proteostasis associates with altered MeAA levels in humans

Consistent with our metabolic and biochemical characterization of SLC25A45, analysis of a recent genome-wide association study (GWAS) revealed a strong association between the

Figure 1. Mitochondrial import of methylated amino acids depends on SLC25A45

(A) Structure illumination microscopy of U-2 OS cells transiently expressing SLC25A45-FLAG using anti-FLAG and anti-TOMM20 (outer mitochondrial membrane marker) antibodies. Scale bars, 2 μm.

(B) Log2 fold change in mitochondrial metabolites from *SLC25A45*-KO+SLC25A45 FLAG AsPC-1 cells (*n* = 5) compared with *SLC25A45*-KO + EV (empty vector; *n* = 4).

(C) Uptake of 10 μM [^{2}H9]-trimethyllysine (TML) by mitochondria isolated from FreeStyle 293-F cells +/− SLC25A45-8xHis measured by LC-MS (*n* = 3).

(D) [^{2}H9]-TML levels in mitochondria treated with 10 μM [^{2}H9]-TML +/− 10 mM unlabeled TML for 10 min (*n* = 4).

(E) The change in melting temperature (ΔTm) of purified human SLC25A45 in the presence of 10 mM compound (*n* = 3).

Data represent means ± SD; *n* = independent mitochondrial preparations (B, C, and D) or biological replicates (E). *p* values calculated using multiple two-tailed unpaired t tests with Holm-Šídák multiple comparison correction (B) and two-way ANOVA (C) with Tukey's multiple comparison test (D). See also Figures S1–S4.

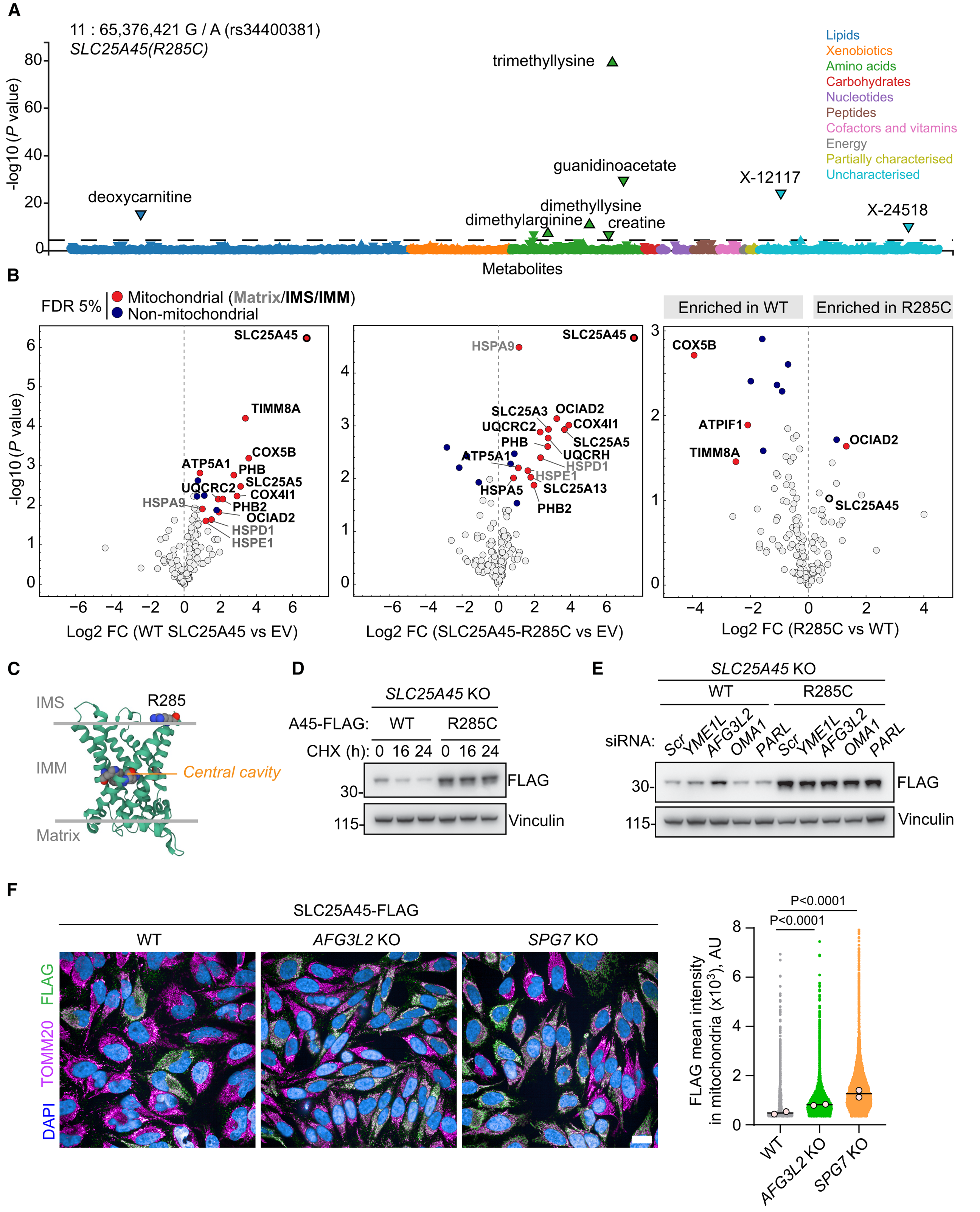

(legend on next page)

CellPress
OPEN ACCESS

SLC25A45 missense variant *R285C* (rs34400381) and levels of trimethyllysine (*p* value: 1.9×10^{-80}), deoxycarnitine (*p* value: 1.3×10^{-15}), and dimethylarginine (*p* value: 1.4×10^{-8}) in the plasma metabolome of 6,136 Finnish male participants (METSIM)[49] (Figure 2A). The association of *SLC25A45 R285C* with trimethyllysine levels in human plasma and urine was also identified by the German Chronic Kidney Disease (GCKD) study[50] and was linked with increased risk of sudden cardiac death and variable heart rate.[51]

To study the impact of arginine 285 mutation to cysteine, we first compared the protein interactomes of WT and mutant SLC25A45 (SLC25A45^{R285C}) in digitonin-permeabilized mitochondria. WT SLC25A45-FLAG and SLC25A45^{R285C}-FLAG interacted with IMM proteins, including the prohibitin scaffolds (PHB1 and PHB2) and other SLC25 proteins (SLC25A13, SLC25A3, and SLC25A5) (Figure 2B; Table S3). These data argue that WT and SLC25A45^{R285C} are both localized within the IMM. Interestingly, the small chaperone protein TIMM8A interacted exclusively with WT SLC25A45 and not SLC25A45^{R285C} (Figure 2B). TIMM8A localizes to the intermembrane space (IMS) and is known to interact with mitochondrial SLCs, including SLC25A13.[53,54] Arginine 285 is proximal to the C terminus of SLC25A45, likely at the interface between the IMM and IMS (Figures 2C and S5), and could therefore regulate interaction with TIMM8A. The yeast homolog of TIMM8A functions in a hetero-oligomeric complex to facilitate the mitochondrial import of hydrophobic proteins.[55] Knockdown of TIMM8A by siRNA reduced WT SLC25A45 steady-state levels but not those of SLC25A45^{R285C} (Figure S6A), which suggests that TIMM8A regulates the biogenesis or stability of SLC25A45.

To explore the impact of *R285C* on SLC25A45 protein stability further, we measured SLC25A45 levels in AsPC-1 cells treated with the inhibitor of global protein synthesis, cycloheximide. Strikingly, SLC25A45 ^{R285C}-FLAG was stabilized compared with WT SLC25A45-FLAG in the presence of cycloheximide (Figures 2D and S6B). Pharmacological inhibition of the lysosome (Figure S6C) or the proteosome (Figure S6D) failed to block the turnover of SLC25A45-FLAG, which argues that mature SLC25A45 is not degraded by cytosolic degradation pathways. Proteosome inhibition did cause an accumulation of insoluble SLC25A45-FLAG (Figure S6D), suggesting that proteosome activity is required for efficient biogenesis of SLC25A45.[56]

We next tested whether SLC25A45 abundance is regulated at the IMM by the *i*-AAA protease YME1L, *m*-AAA protease AFG3L2, zinc metallopeptidase OMA1, or rhomboid protease PARL.[57] SLC25A45-FLAG levels only increased in cells treated with siRNA targeting AFG3L2, whereas SLC25A45^{R285C} levels were unaffected (Figures 2E and S6E). Depletion of AFG3L2 also blunted the turnover of SLC25A45-FLAG (Figure S6F). AFG3L2 oligomerizes to form the homo-hexameric *m*-AAA protease or interacts with another subunit, paraplegin (SPG7), to form the hetero-hexameric *m*-AAA protease.[58,59] SLC25A45-FLAG accumulated in the mitochondria of *AFG3L2* KO and *SPG7* KO HeLa cells (Figure 2F), suggesting further that WT SLC25A45 is a substrate of the *m*-AAA protease. Together, our characterization of the interactome and turnover of SLC25A45 argues that *R285C* dysregulates SLC25A45 protein homeostasis, which leads to altered MeAA levels in human plasma.

Mitochondrial metabolism of trimethyllysine depends on SLC25A45

Having established that SLC25A45 enables mitochondria to import dimethylarginine and trimethyllysine, we decided to focus on the role of SLC25A45 in mitochondrial trimethyllysine metabolism and carnitine biosynthesis (Figure 3A). Trimethyllysine is produced upon the proteolysis of methylated proteins and is also obtained from the diet.[17] Dietary trimethyllysine is absorbed by the small intestine and is reabsorbed efficiently by the kidney and liver.[60] We incubated *SLC25A45* KO AsPC-1 cells and those expressing SLC25A45-FLAG with 100 μM [^{2}H9]-TML, which accumulated to similar levels in both cell lines (Figure S7A). This demonstrates that AsPC-1 cells can import trimethyllysine. Trimethyllysine is hydroxylated within mitochondria by the alpha-ketoglutarate-dependent dioxygenase trimethyllysine hydroxylase (TMLHE) in the first step of the carnitine biosynthesis pathway (Figure 3A). While hydroxy-trimethyllysine (HTML) and trimethylaminobutyraldehyde (TMABA) were not detected in AsPC-1 cells, we could measure the intracellular levels of carnitine and its precursor, deoxycarnitine. Strikingly, deuterium-labeled deoxycarnitine accumulated over time in *SLC25A45* KO cells expressing SLC25A45 (Figure 3B), while carnitine remained unlabeled

Figure 2. *R285C* stabilizes SLC25A45 and alters the human plasma metabolome

(A) PheWeb plot of significant associations between rs34400381 (*SLC25A45*(R285C)) and the indicated metabolites from the METSIM study GWAS (https://pheweb.org/metsim-metab/variant/11:65376421-G-A). The dashed line indicates the *p* value threshold determined from the METSIM study.[49]

(B) SLC25A45 and SLC25A45^{R285C} interaction partners determined by FLAG-immunoprecipitation (IP) and MS of mitochondria from *SLC25A45* KO AsPC-1 cells expressing EV (empty vector), SLC25A45-FLAG (WT), or SLC25A45^{R285C} ($n = 3$ independent mitochondrial preparations).

(C) Predicted protein structure of hSLC25A45 viewed laterally with the IMM and arginine 285 illustrated in space-fill (generated with Alphafold2.0; Q8N413 (S2545_HUMAN)[52]).

(D) Representative immunoblot of *SLC25A45* KO AsPC-1 cells expressing SLC25A45^{WT}-FLAG or SLC25A45^{R285C}-FLAG treated with 150 μg/mL cycloheximide (CHX). See Figure S6B.

(E) Representative immunoblot of *SLC25A45* KO AsPC-1 cells expressing SLC25A45^{WT}-FLAG or SLC25A45^{R285C}-FLAG treated with siRNA for 72 h. See Figure S6E.

(F) Analysis of SLC25A45-FLAG levels by high-content confocal microscopy in the indicated HeLa cells. Mean FLAG intensity within a mitochondrial mask set by TOMM20 immunostaining was quantified per cell (WT HeLa $n = 16{,}004$; *AFG3L2* KO $n = 77{,}718$; *SPG7* KO $n = 100{,}473$; n = cells, large data points = means from 2 independent experiments).

p values calculated using a permutation-based student's t test with the false discovery rate (FDR) set at 5% (B) and a one-way ANOVA with Dunnett's multiple comparison test (F). See also Figures S5 and S6.

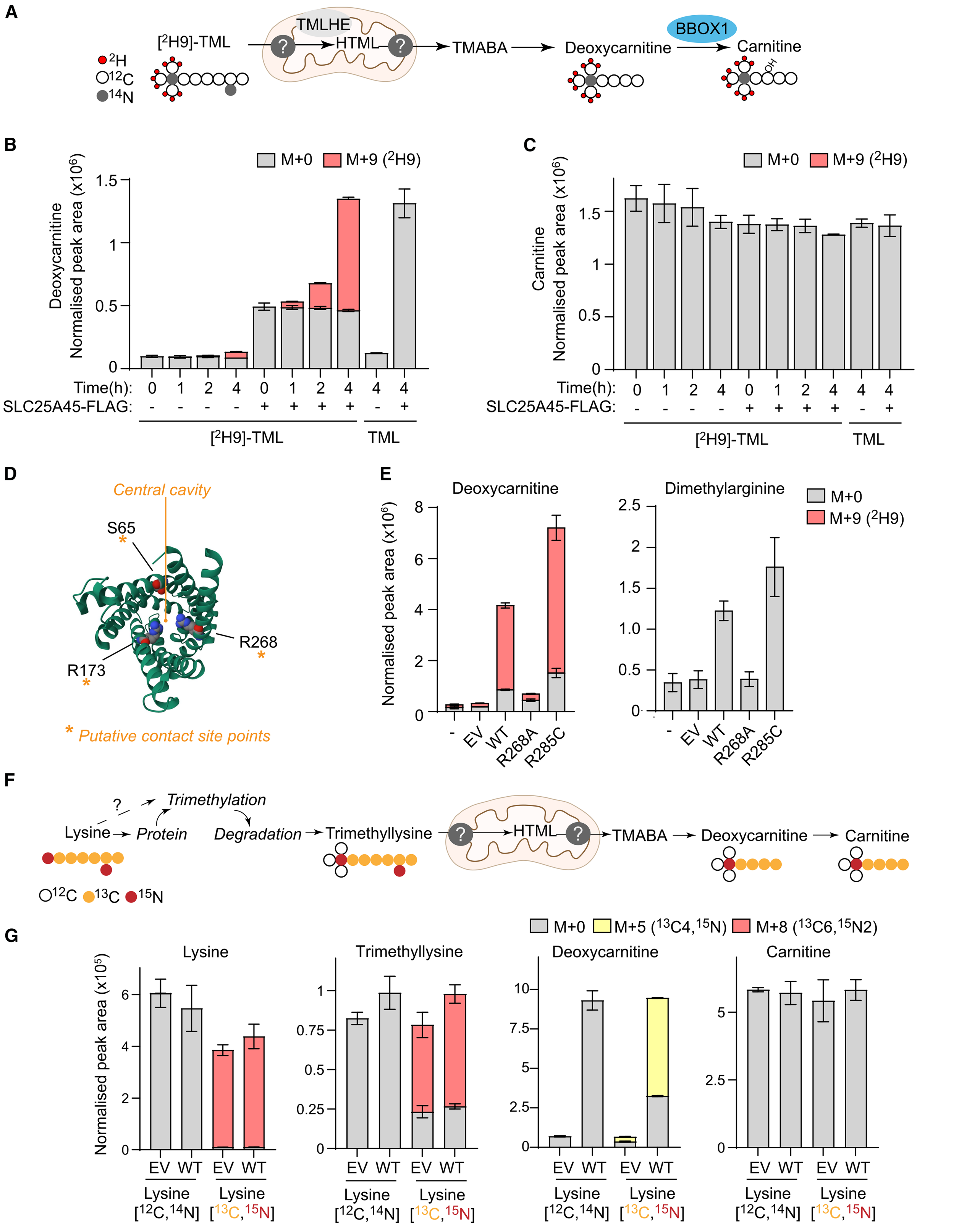

(legend on next page)

(Figure 3C). AsPC-1 cells therefore require SLC25A45 to metabolize trimethyllysine but are unable to synthesize carnitine.

To test whether trimethyllysine metabolism depends on SLC25A45-regulated metabolite transport, we mutated SLC25A45 to block its transport activity. Comparison of the SLC25A45 amino acid sequence across species and its alignment with other yeast and human carriers[28,61] indicated that serine 65, arginine 173, and arginine 268 are potential substrate contact points in the central cavity of human SLC25A45 (Figures 3D and S5). Expression of SLC25A45^{R268A} in *SLC25A45* KO cells abolished SLC25A45-dependent synthesis of deoxycarnitine and prevented the accumulation of dimethylarginine (Figures 3E and S7B), which is consistent with the dependency on arginine 268 for SLC25A45 transport activity. By contrast, SLC25A45^{R285C} expression enhanced deoxycarnitine production and dimethylarginine levels more than WT SLC25A45 (Figure 3E), which is consistent with the increased stability of SLC25A45^{R285C} at the IMM (Figures 2D and S5C).

Methyltransferases catalyze the S-adenosylmethionine-dependent trimethylation of lysine residues, which are released upon proteolysis.[17] To our knowledge, trimethylation of intracellular free lysine has not been described. We incubated cells with [^{13}C6,^{15}N2]-lysine for 72 h (Figure 3F), which resulted in a predominantly [^{13}C] and [^{15}N] labeled pool of trimethyllysine in *SLC25A45* KO AsPC-1 cells and those expressing SLC25A45 (Figure 3G). Consistent with previous results (Figures 3B and 3E), trimethyllysine was converted to deoxycarnitine in the presence of SLC25A45, but carnitine levels were unaltered (Figure 3G).

SLC25A45 drives carnitine biosynthesis in *BBOX1*-expressing cells

We reasoned that AsPC-1 cells are unable to produce carnitine *de novo* (Figures 3C and 3G) because they lack the alpha-ketoglutarate-dependent gamma-butyrobetaine dioxygenase 1 (BBOX1) (Figure 3A). BBOX1 catalyzes the final step of carnitine biosynthesis, which is normally restricted to the kidney, brain, and liver,[62] and we did not detect BBOX1 in AsPC-1 cells by LC-MS proteomics or immunoblotting (Figure 4A; Table S1). Accordingly, expression of both BBOX1 and SLC25A45 depleted the BBOX1 substrate, deoxycarnitine, and stimulated carnitine synthesis in AsPC-1 cells (Figures 4A and 4B).

BBOX1 expression is upregulated in several cancers, including high-grade serous ovarian cancer.[63] We compared *BBOX1* expression levels across human cancer cell lines and selected two ovarian cancer cell lines with high levels of *BBOX1* mRNA: OVCAR-3 and Kuramochi (Figure 4C). Deletion of *SLC25A45* strongly suppressed carnitine production in both cell lines (Figures 4D, 4E, and S8A). Interestingly, deoxycarnitine synthesis was not completely abolished in *SLC25A45* KO cells, which may indicate the existence of another mitochondrial transporter capable of importing trimethyllysine or the presence of a cytosolic dioxygenase capable of hydroxylating trimethyllysine. We expressed SLC25A45-FLAG in *SLC25A45* KO OVCAR-3 cells (Figure S8B) to analyze the impact of SLC25A45 on the steady-state polar metabolome of cells grown in normal growth media without trimethyllysine. Consistent with AsPC-1 cells (Figures S2A and S2B), deoxycarnitine and dimethylarginine were depleted in cells lacking the transporter, while *SLC25A45* KO OVCAR-3 cells also accumulated intracellular trimethyllysine (Figures S8C and S8D; Table S2). Finally, transient depletion of SLC25A45 by siRNA in OVCAR-3 and Kuramochi cells (Figure S8E) limited the production of carnitine from labeled trimethyllysine (Figure 4F). Together, these results show that SLC25A45 is required for the *de novo* biosynthesis of carnitine in *BBOX1*-expressing cells.

DISCUSSION

Our results show that the orphan mitochondrial SLC25A45 drives the import of methylated amino acids (MeAAs) into mitochondria. Dimethylarginines (SDMA and ADMA) are metabolized in liver and kidney mitochondria by alanine-glyoxylate aminotransferase 2 (AGXT2) with several links to the pathogenesis of cardiovascular and renal disease.[64] The cell lines used in this study do not express AGXT2, which likely explains why overexpression of SLC25A45 led to a significant increase of dimethylarginine, which was detected even in whole-cell extracts. Dimethylarginines are also degraded by cytosolic dimethylarginine dimethylaminohydrolase (DDAH) enzymes,[65] and it remains to be seen what role mitochondria play in the metabolism of dimethylarginine in tissues that do not express AGXT2. For instance, mitochondrial uptake of ADMA may modulate the competitive inhibition of cytosolic nitric oxide synthase (NOS).[66] Of note, another mitochondrial carrier, SLC25A2, was reported to transport ADMA, but not SDMA, *in vitro*,[67] which indicates that some SLC25A45 substrate specificity may overlap with other SLC25 transporters.

Mutation of human *SLC25A45* arginine 285 to cysteine associates with altered levels of plasma MeAAs and cardiac function in humans, which was also reported for non-synonymous polymorphisms of *AGXT2*.[49–51] We found that the single *R285C*

Figure 3. TML metabolism depends on SLC25A45

(A) Carnitine biosynthesis from [^{2}H9]-trimethyllysine (TML).

(B and C) Normalized abundance and mass isotopologue distribution of deoxycarnitine (B) and carnitine (C) in *SLC25A45* KO AsPC-1 cells expressing EV (empty vector) or SLC25A45-FLAG treated with 100 μM unlabeled TML or 100 μM [^{2}H9]-TML (n = 3).

(D) Predicted protein structure of human SLC25A45 viewed from the IMS side of the membrane. Putative substrate contact points (S65, R173, and R268) are illustrated in space-fill (generated with Alphafold2.0; Q8N413 (S2545_HUMAN)[52]).

(E) Normalized abundance and mass isotopologue distribution of deoxycarnitine and dimethylarginine in indicated *SLC25A45* KO AsPC-1 cells after treatment with 100 μM [^{2}H9]-TML for 4 h (n = 3).

(F) Carnitine biosynthesis from [^{13}C6,^{15}N2]-lysine.

(G) Normalized abundance and mass isotopologue distribution of the indicated metabolites in indicated *SLC25A45* KO AsPC-1 cells treated with 800 μM unlabeled or [^{13}C6,^{15}N2]-labeled lysine for 72 h (n = 3).

Data represent means ± SD; n = independent cultures. EV, empty vector. See also Figure S7.

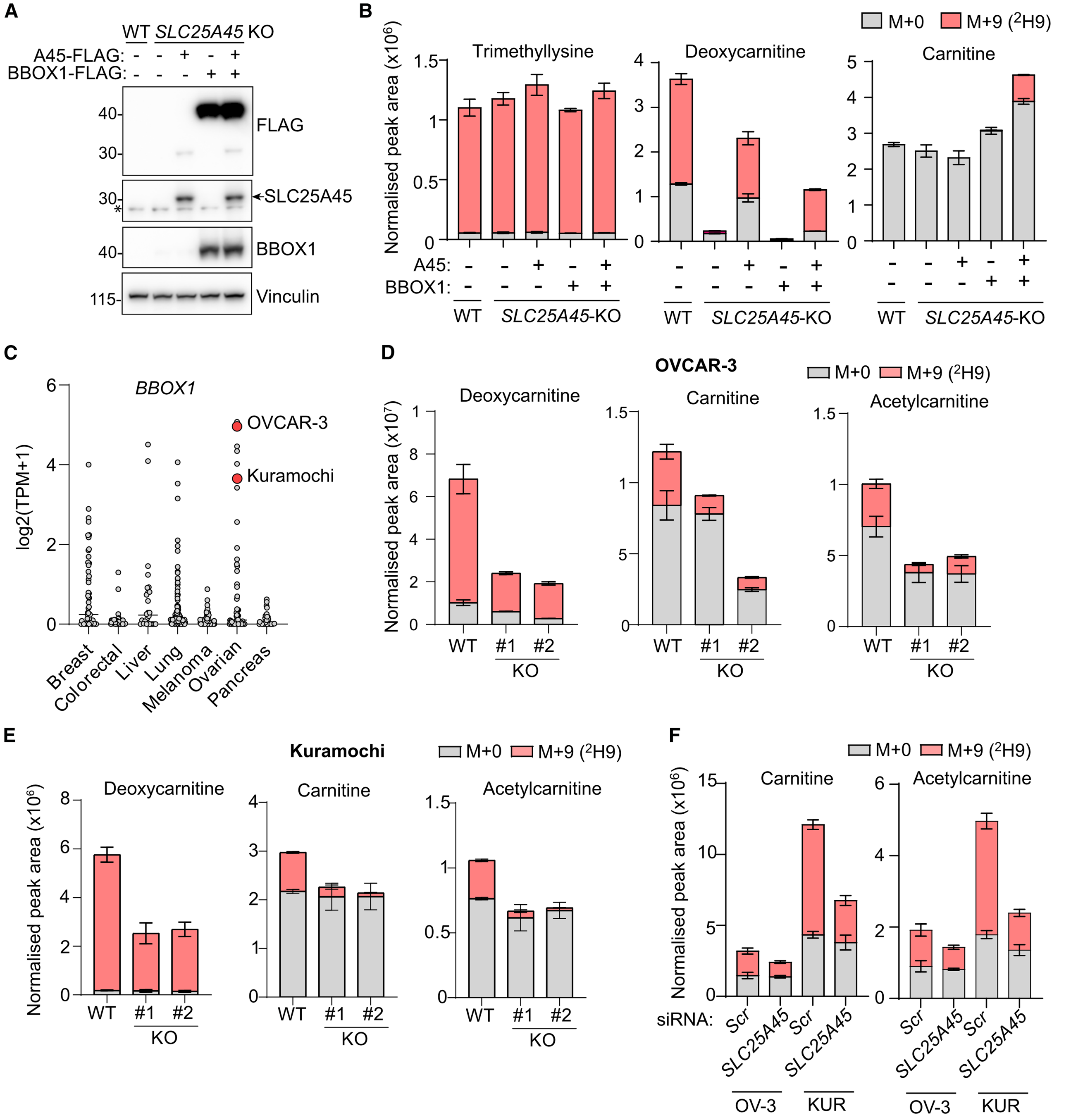

Figure 4. SLC25A45 drives carnitine biosynthesis in BBOX1-positive cells

(A) Immunoblot of WT and *SLC25A45* KO AsPC-1 cells stably expressing SLC25A45-FLAG and/or BBOX1-FLAG (*, non-specific).

(B) Normalized abundance and mass isotopologue distribution of the indicated metabolites in AsPC-1 cell lines treated with 100 μM [^{2}H9]-trimethyllysine (TML) for 4 h (n = 3).

(C) *BBOX1* expression in cancer cell lines from DepMap Public 24Q2 DepMap, Broad (2024). DepMap 24Q2 public. Figshare+. Dataset. https://doi.org/10.25452/figshare.plus.25880521.v1. TPM, transcripts per million.

(D and E) Normalized abundance and mass isotopologue distribution of the indicated metabolites in OVCAR-3 (D, n = 4) and Kuramochi (KUR) (E, n = 3) (E) cells treated with 100 μM [^{2}H9]-TML for 24 h.

(F) Normalized abundance and mass isotopologue distribution of carnitine and acetylcarnitine in OVCAR-3 (OV-3) and KUR cells treated with siRNA for 72 h and 100 μM [^{2}H9]-TML for the final 24 h (n = 3).

Data represent means ± SD; n = independent cultures. See also Figure S8.

mutation was sufficient to block SLC25A45 proteolysis by the *m*-AAA protease. The catalytic domain of the *m*-AAA protease faces the mitochondrial matrix, and the protease interacts with matrix-exposed peptide sequences of soluble and membrane-bound substrates.[68,69] It is unclear how the mutation of an arginine to a redox-sensitive cysteine proximal to the IMS interferes with SLC proteolysis by AFG3L2. SLC25A45-FLAG interacts with the prohibitins, which are known to regulate *m*-AAA activity in the IMM[70]; however, this interaction was unaffected by the *R285C* mutation. It will be important to establish in future studies whether SLC25A45 stability is most impacted by the loss of an arginine or gain of a cysteine at the C terminus. Other mitochondrial transporters have been described as substrates of the *m*-AAA protease, including the glutathione transporter SLC25A39.[71,72] Interestingly, SLC25A39 stability appears to be regulated entirely by the AFG3L2 homo-hexameric *m*-AAA protease, whereas SLC25A45 accumulates upon the depletion of AFG3L2 or SPG7.

A key function of SLC25A45 is to direct the metabolism of trimethyllysine for the biosynthesis of carnitine. Carnitine deficiency can lead to heart defects and liver disease[73] and is associated with cancer cachexia.[74] In omnivorous humans, the majority of carnitine is obtained from the diet, and over 95% is reabsorbed from glomerular filtrate in the kidneys.[75] Carnitine biosynthesis is restricted to tissues that express BBOX1; however, free trimethyllysine is likely metabolized in the mitochondria of many tissues since the mitochondrial TMLHE is ubiquitously expressed. Intriguingly, some cancer subtypes increase carnitine metabolism and therefore appear to be dependent on the expression of TMLHE and BBOX1.[76–78] Why malignant cells upregulate carnitine biosynthesis is unclear, especially considering carnitine can be imported from the microenvironment via the plasma membrane transporter OCTN2/SLC22A5.[79] Importantly, in addition to trimethyllysine, SLC25A45 is responsible for the mitochondrial metabolism of dimethylarginine, and future investigation of SLC25A45 function in different tissues will help illuminate the physiological contexts that depend most on the mitochondrial handling of MeAAs.

Limitations of the study

Our data argue that SLC25A45 is a selective mitochondrial transporter of basic MeAAs; however, quantification of SLC25A45 transport activity in proteoliposomes is required to demonstrate this directly and to define the mode of transport. Without a reliable antibody, many experiments in this study relied on the exogenous expression of tagged SLC25A45, and it remains unclear how the C-terminal region of SLC25A45 regulates endogenous protein homeostasis *in vivo*.

RESOURCE AVAILABILITY

Lead contact

Further information and requests for resources and reagents should be directed to and will be fulfilled by the lead contact, Thomas MacVicar (thomas.macvicar@glasgow.ac.uk).

Materials availability

All materials generated in this study are available upon reasonable request from the lead contact.

Data and code availability

- The MS proteomics data have been deposited to the ProteomeXchange Consortium via the PRIDE partner repository with the dataset identifier PXD058569 and https://doi.org/10.6019/PXD058569 for the whole-cell proteome and with the dataset identifier PXD058579 for the interactome. Raw metabolomics data were submitted to massive (massive.ucsd.edu) with the accession number MSV000098382. Raw image files were deposited to Mendeley Data and are available at Mendeley Data: DOI: https://doi.org/10.17632/kgcs7gvccy.1.
- This paper does not report original code.
- Any additional information required to reanalyze the data reported in this paper is available from the lead contact upon request.

ACKNOWLEDGMENTS

We thank Prof. Patricia Roxburgh (University of Glasgow) and Prof. Thomas Langer (Max Planck Institute for Biology of Ageing) for providing cell lines. We thank Dr. Catherine Winchester for critically reviewing the manuscript and Dr. Nick Jones (Swansea University) and Prof. Jan Riemer (University of Cologne) for helpful discussion. This work was supported by a CRUK Career Development Fellowship to T.M. (RCCFELCDF-May21\100001), a CRUK core funding grant (A29800) to S.Z., a CRUK core funding to the CRUK Scotland Institute (A31287), a CRUK grant for the CRUK Scotland Centre (CTRQQR-2021\100006), and a UKRI Medical Research Council grant (MC_UU_00028/2) to M.S.K. and E.R.S.K.

AUTHOR CONTRIBUTIONS

Conceptualization, M.M.D., E.R.S.K., and T.M.; data curation, M.M.D., M.S.K., E.S., S.L., and T.M.; formal analysis, M.M.D., M.S.K., E.S., S.L., and T.M.; validation, M.M.D., M.S.K., E.S., S.L., N.P., P.T., S.Z., D.S., E.R.S.K., and T.M.; investigation, M.M.D., M.S.K., and T.M.; visualization, M.M.D., M.S.K., E.S., S.L., N.P., P.T., and T.M.; methodology, M.M.D., M.S.K., E.S., S.L., N.P., P.T., S.Z., D.S., and E.R.S.K.; writing – original draft, M.M.D., M.S.K., and T.M.; writing – review and editing, all authors; supervision, S.Z., D.S., E.R.S.K., and T.M.; project administration, E.R.S.K. and T.M.; resources, E.R.S.K. and T.M.; funding acquisition, E.R.S.K. and T.M.

DECLARATION OF INTERESTS

The authors declare no competing interests.

STAR★METHODS

Detailed methods are provided in the online version of this paper and include the following:

- KEY RESOURCES TABLE
- EXPERIMENTAL MODEL AND STUDY PARTICIPANT DETAILS
  - Cell lines
  - Yeast and bacterial strains
- METHOD DETAILS
  - Plasmid construction
  - Cell line transfection
  - Generation of knockout cells by CRISPR-Cas9 editing
  - Lentivirus production and transduction
  - [^{2}H9]-trimethyllysine [TML] and [^{13}C6, ^{15}N2]-lysine labelling in cells
  - Mitochondrial isolation
  - Mitochondrial amino acid uptake assays
  - Metabolite extraction from cells and mitochondria
  - Expression of SLC25A45 in Saccharomyces cerevisiae
  - Preparation of lipids for protein purification
  - Purification of SLC25A45 by nickel affinity chromatography
  - Thermostability analysis
  - Cycloheximide assay
  - Cell lysis and SDS-PAGE

- ○ Extracellular flux analysis
- ○ Reverse transcription-quantitative PCR (RT-qPCR)
- ○ Immunofluorescence and super-resolution microscopy
- ○ Proteomics
- QUANTIFICATION AND STATISTICAL ANALYSIS

SUPPLEMENTAL INFORMATION

Supplemental information can be found online at https://doi.org/10.1016/j.molcel.2025.08.018.

Received: March 31, 2025
Revised: July 18, 2025
Accepted: August 17, 2025
Published: October 10, 2025

REFERENCES

1. Cunningham, C.N., and Rutter, J. (2020). 20,000 picometers under the OMM: diving into the vastness of mitochondrial metabolite transport. EMBO Rep. *21*, e50071. https://doi.org/10.15252/embr.202050071.
2. Palmieri, F., and Monné, M. (2016). Discoveries, metabolic roles and diseases of mitochondrial carriers: A review. Biochim. Biophys. Acta *1863*, 2362–2378. https://doi.org/10.1016/j.bbamcr.2016.03.007.
3. Kunji, E.R.S., King, M.S., Ruprecht, J.J., and Thangaratnarajah, C. (2020). The SLC25 Carrier Family: Important Transport Proteins in Mitochondrial Physiology and Pathology. Physiology (Bethesda) *35*, 302–327. https://doi.org/10.1152/physiol.00009.2020.
4. Bar-Peled, L., and Kory, N. (2022). Principles and functions of metabolic compartmentalization. Nat. Metab. *4*, 1232–1244. https://doi.org/10.1038/s42255-022-00645-2.
5. Lytovchenko, O., and Kunji, E.R.S. (2017). Expression and putative role of mitochondrial transport proteins in cancer. Biochim. Biophys. Acta Bioenerg. *1858*, 641–654. https://doi.org/10.1016/j.bbabio.2017.03.006.
6. Kunji, E.R.S., and Harding, M. (2003). Projection structure of the atractyloside-inhibited mitochondrial ADP/ATP carrier of *Saccharomyces cerevisiae*. J. Biol. Chem. *278*, 36985–36988. https://doi.org/10.1074/jbc.C300304200.
7. Ruprecht, J.J., and Kunji, E.R.S. (2020). The SLC25 Mitochondrial Carrier Family: Structure and Mechanism. Trends Biochem. Sci. *45*, 244–258. https://doi.org/10.1016/j.tibs.2019.11.001.
8. Pebay-Peyroula, E., Dahout-Gonzalez, C., Kahn, R., Trézéguet, V., Lauquin, G.J.M., and Brandolin, G. (2003). Structure of mitochondrial ADP/ATP carrier in complex with carboxyatractyloside. Nature *426*, 39–44. https://doi.org/10.1038/nature02056.
9. Ruprecht, J.J., King, M.S., Zögg, T., Aleksandrova, A.A., Pardon, E., Crichton, P.G., Steyaert, J., and Kunji, E.R.S. (2019). The Molecular Mechanism of Transport by the Mitochondrial ADP/ATP Carrier. Cell *176*, 435–447.e15. https://doi.org/10.1016/j.cell.2018.11.025.
10. Cimadamore-Werthein, C., Jaiquel Baron, S., King, M.S., Springett, R., and Kunji, E.R. (2023). Human mitochondrial ADP/ATP carrier SLC25A4 operates with a ping-pong kinetic mechanism. EMBO Rep. *24*, e57127. https://doi.org/10.15252/embr.202357127.
11. Cimadamore-Werthein, C., King, M.S., Lacabanne, D., Pyrihová, E., Jaiquel Baron, S., and Kunji, E.R. (2024). Human mitochondrial carriers of the SLC25 family function as monomers exchanging substrates with a ping-pong kinetic mechanism. EMBO J. *43*, 3450–3465. https://doi.org/10.1038/s44318-024-00150-0.
12. Ruprecht, J.J., and Kunji, E.R.S. (2021). Structural Mechanism of Transport of Mitochondrial Carriers. Annu. Rev. Biochem. *90*, 535–558. https://doi.org/10.1146/annurev-biochem-072820-020508.
13. Ruprecht, J.J., Hellawell, A.M., Harding, M., Crichton, P.G., McCoy, A.J., and Kunji, E.R.S. (2014). Structures of yeast mitochondrial ADP/ATP carriers support a domain-based alternating-access transport mechanism. Proc. Natl. Acad. Sci. USA *111*, E426–E434. https://doi.org/10.1073/pnas.1320692111.
14. Mavridou, V., King, M.S., Tavoulari, S., Ruprecht, J.J., Palmer, S.M., and Kunji, E.R.S. (2022). Substrate binding in the mitochondrial ADP/ATP carrier is a step-wise process guiding the structural changes in the transport cycle. Nat. Commun. *13*, 3585. https://doi.org/10.1038/s41467-022-31366-5.
15. César-Razquin, A., Snijder, B., Frappier-Brinton, T., Isserlin, R., Gyimesi, G., Bai, X., Reithmeier, R.A., Hepworth, D., Hediger, M.A., Edwards, A.M., and Superti-Furga, G. (2015). A Call for Systematic Research on Solute Carriers. Cell *162*, 478–487. https://doi.org/10.1016/j.cell.2015.07.022.
16. Paik, W.K., and Kim, S. (1993). N(G)-Methylarginines: Biosynthesis, biochemical function and metabolism. Amino Acids *4*, 267–286. https://doi.org/10.1007/BF00805828.
17. Servillo, L., Giovane, A., Cautela, D., Castaldo, D., and Balestrieri, M.L. (2014). Where does N(ε)-trimethyllysine for the carnitine biosynthesis in mammals come from? PLoS One *9*, e84589. https://doi.org/10.1371/journal.pone.0084589.
18. Vallance, P., Leone, A., Calver, A., Collier, J., and Moncada, S. (1992). Accumulation of an endogenous inhibitor of nitric oxide synthesis in chronic renal failure. Lancet *339*, 572–575. https://doi.org/10.1016/0140-6736(92)90865-z.
19. Sibal, L., Agarwal, S.C., Home, P.D., and Boger, R.H. (2010). The Role of Asymmetric Dimethylarginine (ADMA) in Endothelial Dysfunction and Cardiovascular Disease. Curr. Cardiol. Rev. *6*, 82–90. https://doi.org/10.2174/157340310791162659.
20. Maas, M.N., Hintzen, J.C.J., Porzberg, M.R.B., and Mecinović, J. (2020). Trimethyllysine: From Carnitine Biosynthesis to Epigenetics. Int. J. Mol. Sci. *21*, 9451. https://doi.org/10.3390/ijms21249451.
21. Pande, S.V. (1975). A mitochondrial carnitine acylcarnitine translocase system. Proc. Natl. Acad. Sci. USA *72*, 883–887. https://doi.org/10.1073/pnas.72.3.883.
22. Ramsay, R.R., and Tubbs, P.K. (1975). The mechanism of fatty acid uptake by heart mitochondria: An acylcarnitine-carnitine exchange. FEBS Lett. *54*, 21–25. https://doi.org/10.1016/0014-5793(75)81059-3.
23. Indiveri, C., Tonazzi, A., and Palmieri, F. (1990). Identification and purification of the carnitine carrier from rat liver mitochondria. Biochim. Biophys. Acta *1020*, 81–86. https://doi.org/10.1016/0005-2728(90)90096-M.
24. Nguyen, T.B., Louie, S.M., Daniele, J.R., Tran, Q., Dillin, A., Zoncu, R., Nomura, D.K., and Olzmann, J.A. (2017). DGAT1-Dependent Lipid Droplet Biogenesis Protects Mitochondrial Function during Starvation-Induced Autophagy. Dev. Cell *42*, 9–21.e5. https://doi.org/10.1016/j.devcel.2017.06.003.
25. Madiraju, P., Pande, S.V., Prentki, M., and Madiraju, S.R.M. (2009). Mitochondrial acetylcarnitine provides acetyl groups for nuclear histone acetylation. Epigenetics *4*, 399–403. https://doi.org/10.4161/epi.4.6.9767.
26. Izzo, L.T., Trefely, S., Demetriadou, C., Drummond, J.M., Mizukami, T., Kuprasertkul, N., Farria, A.T., Nguyen, P.T.T., Murali, N., Reich, L., et al. (2023). Acetylcarnitine shuttling links mitochondrial metabolism to histone acetylation and lipogenesis. Sci. Adv. *9*, eadf0115. https://doi.org/10.1126/sciadv.adf0115.
27. Byrne, K.L., Szeligowski, R.V., and Shen, H. (2023). Phylogenetic Analysis Guides Transporter Protein Deorphanization: A Case Study of the SLC25 Family of Mitochondrial Metabolite Transporters. Biomolecules *13*, 1314. https://doi.org/10.3390/biom13091314.
28. Robinson, A.J., Overy, C., and Kunji, E.R.S. (2008). The mechanism of transport by mitochondrial carriers based on analysis of symmetry. Proc. Natl. Acad. Sci. USA *105*, 17766–17771. https://doi.org/10.1073/Pnas.0809580105.
29. Yook, J.-S., Taxin, Z.H., Yuan, B., Oikawa, S., Auger, C., Mutlu, B., Puigserver, P., Hui, S., and Kajimura, S. (2023). The SLC25A47 locus

CellPress
OPEN ACCESS

controls gluconeogenesis and energy expenditure. Proc. Natl. Acad. Sci. USA *120*, e2216810120. https://doi.org/10.1073/pnas.2216810120.

30. Bresciani, N., Demagny, H., Lemos, V., Pontanari, F., Li, X., Sun, Y., Li, H., Perino, A., Auwerx, J., and Schoonjans, K. (2022). The Slc25a47 locus is a novel determinant of hepatic mitochondrial function implicated in liver fibrosis. J. Hepatol. *77*, 1071–1082. https://doi.org/10.1016/j.jhep.2022.05.040.
31. Khan, A., Unlu, G., Lin, P., Liu, Y., Kilic, E., Kenny, T.C., Birsoy, K., and Gamazon, E.R. (2024). Metabolic gene function discovery platform GeneMAP identifies SLC25A48 as necessary for mitochondrial choline import. Nat. Genet. *56*, 1614–1623. https://doi.org/10.1038/s41588-024-01827-2.
32. Verkerke, A.R.P., Shi, X., Li, M., Higuchi, Y., Yamamuro, T., Katoh, D., Nishida, H., Auger, C., Abe, I., Gerszten, R.E., and Kajimura, S. (2024). SLC25A48 controls mitochondrial choline import and metabolism. Cell Metab. *36*, 2156–2166.e9. https://doi.org/10.1016/j.cmet.2024.07.010.
33. Patil, S., Borisov, O., Scherer, N., Wirth, C., Schlosser, P., Wuttke, M., Ehret, S., Hannibal, L., Eckardt, K.U., Hunte, C., et al. (2025). The membrane transporter SLC25A48 enables transport of choline into human mitochondria. Kidney Int. *107*, 296–301. https://doi.org/10.1016/j.kint.2024.06.022.
34. Porcelli, V., Fiermonte, G., Longo, A., and Palmieri, F. (2014). The Human Gene SLC25A29, of Solute Carrier Family 25, Encodes a Mitochondrial Transporter of Basic Amino Acids. J. Biol. Chem. *289*, 13374–13384. https://doi.org/10.1074/jbc.M114.547448.
35. Chen, W.W., Freinkman, E., and Sabatini, D.M. (2017). Rapid immunopurification of mitochondria for metabolite profiling and absolute quantification of matrix metabolites. Nat. Protoc. *12*, 2215–2231. https://doi.org/10.1038/nprot.2017.104.
36. Morgenstern, M., Peikert, C.D., Lübbert, P., Suppanz, I., Klemm, C., Alka, O., Steiert, C., Naumenko, N., Schendzielorz, A., Melchionda, L., et al. (2021). Quantitative high-confidence human mitochondrial proteome and its dynamics in cellular context. Cell Metab. *33*, 2464–2483.e18. https://doi.org/10.1016/j.cmet.2021.11.001.
37. King, M.S., and Kunji, E.R.S. (2020). Expression and Purification of Membrane Proteins in Saccharomyces cerevisiae. Methods Mol. Biol. *2127*, 47–61. https://doi.org/10.1007/978-1-0716-0373-4_4.
38. Majd, H., King, M.S., Palmer, S.M., Smith, A.C., Elbourne, L.D.H., Paulsen, I.T., Sharples, D., Henderson, P.J.F., and Kunji, E.R.S. (2018). Screening of candidate substrates and coupling ions of transporters by thermostability shift assays. eLife *7*, e38821. https://doi.org/10.7554/eLife.38821.
39. Alexander, C.G., Wanner, R., Johnson, C.M., Breitsprecher, D., Winter, G., Duhr, S., Baaske, P., and Ferguson, N. (2014). Novel microscale approaches for easy, rapid determination of protein stability in academic and commercial settings. Biochim. Biophys. Acta *1844*, 2241–2250. https://doi.org/10.1016/j.bbapap.2014.09.016.
40. Pyrihová, E., King, M.S., King, A.C., Toleco, M.R., van der Giezen, M., and Kunji, E.R.S. (2024). A mitochondrial carrier transports glycolytic intermediates to link cytosolic and mitochondrial glycolysis in the human gut parasite Blastocystis. eLife *13*, RP94187. https://doi.org/10.7554/eLife.94187.
41. Harborne, S.P.D., and Kunji, E.R.S. (2018). Calcium-regulated mitochondrial ATP-Mg/Pi carriers evolved from a fusion of an EF-hand regulatory domain with a mitochondrial ADP/ATP carrier-like domain. IUBMB Life *70*, 1222–1232. https://doi.org/10.1002/iub.1931.
42. Crichton, P.G., Lee, Y., Ruprecht, J.J., Cerson, E., Thangaratnarajah, C., King, M.S., and Kunji, E.R.S. (2015). Trends in thermostability provide information on the nature of substrate, inhibitor, and lipid interactions with mitochondrial carriers. J. Biol. Chem. *290*, 8206–8217. https://doi.org/10.1074/jbc.M114.616607.
43. King, M.S., Kerr, M., Crichton, P.G., Springett, R., and Kunji, E.R.S. (2016). Formation of a cytoplasmic salt bridge network in the matrix state is a fundamental step in the transport mechanism of the mitochondrial ADP/ATP carrier. Biochim. Biophys. Acta *1857*, 14–22. https://doi.org/10.1016/j.bbabio.2015.09.013.
44. Jaiquel Baron, S., King, M.S., Kunji, E.R.S., and Schirris, T.J.J. (2021). Characterization of drug-induced human mitochondrial ADP/ATP carrier inhibition. Theranostics *11*, 5077–5091. https://doi.org/10.7150/thno.54936.
45. Jones, S.A., Gogoi, P., Ruprecht, J.J., King, M.S., Lee, Y., Zögg, T., Pardon, E., Chand, D., Steimle, S., Copeman, D.M., et al. (2023). Structural basis of purine nucleotide inhibition of human uncoupling protein 1. Sci. Adv. *9*, eadh4251. https://doi.org/10.1126/sciadv.adh4251.
46. Tavoulari, S., Schirris, T.J.J., Mavridou, V., Thangaratnarajah, C., King, M.S., Jones, D.T.D., Ding, S., Fearnley, I.M., and Kunji, E.R.S. (2022). Key features of inhibitor binding to the human mitochondrial pyruvate carrier hetero-dimer. Mol. Metab. *60*, 101469. https://doi.org/10.1016/j.molmet.2022.101469.
47. Tavoulari, S., Lacabanne, D., Pereira, G.C., Thangaratnarajah, C., King, M.S., He, J., Chowdhury, S.R., Tilokani, L., Palmer, S.M., Prudent, J., et al. (2024). Distinct roles for the domains of the mitochondrial aspartate/glutamate carrier citrin in organellar localization and substrate transport. Mol. Metab. *90*, 102047. https://doi.org/10.1016/j.molmet.2024.102047.
48. Kang, Y., and Chen, L. (2023). Structural basis for the binding of DNP and purine nucleotides onto UCP1. Nature *620*, 226–231. https://doi.org/10.1038/s41586-023-06332-w.
49. Yin, X., Chan, L.S., Bose, D., Jackson, A.U., VandeHaar, P., Locke, A.E., Fuchsberger, C., Stringham, H.M., Welch, R., Yu, K., et al. (2022). Genome-wide association studies of metabolites in Finnish men identify disease-relevant loci. Nat. Commun. *13*, 1644. https://doi.org/10.1038/s41467-022-29143-5.
50. Schlosser, P., Scherer, N., Grundner-Culemann, F., Monteiro-Martins, S., Haug, S., Steinbrenner, I., Uluvar, B., Wuttke, M., Cheng, Y., Ekici, A.B., et al. (2023). Genetic studies of paired metabolomes reveal enzymatic and transport processes at the interface of plasma and urine. Nat. Genet. *55*, 995–1008. https://doi.org/10.1038/s41588-023-01409-8.
51. Seppälä, I., Kleber, M.E., Lyytikäinen, L.P., Hernesniemi, J.A., Mäkelä, K.M., Oksala, N., Laaksonen, R., Pilz, S., Tomaschitz, A., Silbernagel, G., et al. (2014). Genome-wide association study on dimethylarginines reveals novel AGXT2 variants associated with heart rate variability but not with overall mortality. Eur. Heart J. *35*, 524–531. https://doi.org/10.1093/eurheartj/eht447.
52. Jumper, J., Evans, R., Pritzel, A., Green, T., Figurnov, M., Ronneberger, O., Tunyasuvunakool, K., Bates, R., Žídek, A., Potapenko, A., et al. (2021). Highly accurate protein structure prediction with AlphaFold. Nature *596*, 583–589. https://doi.org/10.1038/s41586-021-03819-2.
53. Roesch, K., Hynds, P.J., Varga, R., Tranebjaerg, L., and Koehler, C.M. (2004). The calcium-binding aspartate/glutamate carriers, citrin and aralar1, are new substrates for the DDP1/TIMM8a–TIMM13 complex. Hum. Mol. Genet. *13*, 2101–2111. https://doi.org/10.1093/hmg/ddh217.
54. Anderson, A.J., Crameri, J.J., Ang, C.S., Malcolm, T.R., Kang, Y., Baker, M.J., Palmer, C.S., Sharpe, A.J., Formosa, L.E., Ganio, K., et al. (2023). Human Tim8a, Tim8b and Tim13 are auxiliary assembly factors of mature Complex IV. EMBO Rep. *24*, e56430. https://doi.org/10.15252/embr.202256430.
55. Curran, S.P., Leuenberger, D., Schmidt, E., and Koehler, C.M. (2002). The role of the Tim8p-Tim13p complex in a conserved import pathway for mitochondrial polytopic inner membrane proteins. J. Cell Biol. *158*, 1017–1027. https://doi.org/10.1083/jcb.200205124.
56. Rödl, S., and Herrmann, J.M. (2023). The role of the proteasome in mitochondrial protein quality control. IUBMB Life *75*, 868–879. https://doi.org/10.1002/iub.2734.
57. Ahola, S., Langer, T., and MacVicar, T. (2019). Mitochondrial Proteolysis and Metabolic Control. Cold Spring Harb. Perspect. Biol. *11*, a033936. https://doi.org/10.1101/cshperspect.a033936.
58. Arlt, H., Tauer, R., Feldmann, H., Neupert, W., and Langer, T. (1996). The YTA10-12 complex, an AAA protease with chaperone-like activity in the

inner membrane of mitochondria. Cell *85*, 875–885. https://doi.org/10.1016/s0092-8674(00)81271-4.

59. Koppen, M., Metodiev, M.D., Casari, G., Rugarli, E.I., and Langer, T. (2007). Variable and tissue-specific subunit composition of mitochondrial m-AAA protease complexes linked to hereditary spastic paraplegia. Mol. Cell. Biol. *27*, 758–767. https://doi.org/10.1128/MCB.01470-06.

60. Zaspel, B.J., Sheridan, K.J., and Henderson, L.M. (1980). Transport and metabolism of carnitine precursors in various organs of the rat. Biochim. Biophys. Acta *631*, 192–202. https://doi.org/10.1016/0304-4165(80)90067-7.

61. Robinson, A.J., and Kunji, E.R.S. (2006). Mitochondrial carriers in the cytoplasmic state have a common substrate binding site. Proc. Natl. Acad. Sci. USA *103*, 2617–2622. https://doi.org/10.1073/pnas.0509994103.

62. El-Hattab, A.W., and Scaglia, F. (2015). Disorders of carnitine biosynthesis and transport. Mol. Genet. Metab. *116*, 107–112. https://doi.org/10.1016/j.ymgme.2015.09.004.

63. Huang, J., Tang, Y., Li, Y., Wei, W., Kang, F., Tan, S., Lin, L., Lu, X., Wei, H., and Wang, N. (2024). BBOX1 mediates metabolic reprogramming driven by hypoxia and participates in the malignant progress of high-grade serous ovarian cancer. Biochim. Biophys. Acta Mol. Cell Res. *1871*, 119830. https://doi.org/10.1016/j.bbamcr.2024.119830.

64. Rodionov, R.N., Jarzebska, N., Weiss, N., and Lentz, S.R. (2014). AGXT2: a promiscuous aminotransferase. Trends Pharmacol. Sci. *35*, 575–582. https://doi.org/10.1016/j.tips.2014.09.005.

65. Ogawa, T., Kimoto, M., and Sasaoka, K. (1989). Purification and properties of a new enzyme, NG,NG-dimethylarginine dimethylaminohydrolase, from rat kidney. J. Biol. Chem. *264*, 10205–10209. https://doi.org/10.1016/S0021-9258(18)81786-0.

66. Leiper, J., and Nandi, M. (2011). The therapeutic potential of targeting endogenous inhibitors of nitric oxide synthesis. Nat. Rev. Drug Discov. *10*, 277–291. https://doi.org/10.1038/nrd3358.

67. Porcelli, V., Longo, A., Palmieri, L., Closs, E.I., and Palmieri, F. (2016). Asymmetric dimethylarginine is transported by the mitochondrial carrier SLC25A2. Amino Acids *48*, 427–436. https://doi.org/10.1007/s00726-015-2096-9.

68. Ding, B., Martin, D.W., Rampello, A.J., and Glynn, S.E. (2018). Dissecting Substrate Specificities of the Mitochondrial AFG3L2 Protease. Biochemistry *57*, 4225–4235. https://doi.org/10.1021/acs.biochem.8b00565.

69. Leonhard, K., Herrmann, J.M., Stuart, R.A., Mannhaupt, G., Neupert, W., and Langer, T. (1996). AAA proteases with catalytic sites on opposite membrane surfaces comprise a proteolytic system for the ATP-dependent degradation of inner membrane proteins in mitochondria. EMBO J. *15*, 4218–4229. https://doi.org/10.1002/j.1460-2075.1996.tb00796.x.

70. Steglich, G., Neupert, W., and Langer, T. (1999). Prohibitins regulate membrane protein degradation by the m-AAA protease in mitochondria. Mol. Cell. Biol. *19*, 3435–3442. https://doi.org/10.1128/MCB.19.5.3435.

71. Shi, X., DeCiucis, M., Grabinska, K.A., Kanyo, J., Liu, A., Lam, T.T., and Shen, H. (2024). Dual regulation of SLC25A39 by AFG3L2 and iron controls mitochondrial glutathione homeostasis. Mol. Cell *84*, 802–810.e6. https://doi.org/10.1016/j.molcel.2023.12.008.

72. Liu, Y., Liu, S., Tomar, A., Yen, F.S., Unlu, G., Ropek, N., Weber, R.A., Wang, Y., Khan, A., Gad, M., et al. (2023). Autoregulatory control of mitochondrial glutathione homeostasis. Science *382*, 820–828. https://doi.org/10.1126/science.adf4154.

73. Longo, N., Frigeni, M., and Pasquali, M. (2016). Carnitine transport and fatty acid oxidation. Biochim. Biophys. Acta *1863*, 2422–2435. https://doi.org/10.1016/j.bbamcr.2016.01.023.

74. Silvério, R., Laviano, A., Rossi Fanelli, F., and Seelaender, M. (2011). l-carnitine and cancer cachexia: Clinical and experimental aspects. J. Cachexia Sarcopenia Muscle *2*, 37–44. https://doi.org/10.1007/s13539-011-0017-7.

75. Vaz, F.M., and Wanders, R.J.A. (2002). Carnitine biosynthesis in mammals. Biochem. J. *361*, 417–429. https://doi.org/10.1042/0264-6021:3610417.

76. Ohara, Y., Tang, W., Liu, H., Yang, S., Dorsey, T.H., Cawley, H., Moreno, P., Chari, R., Guest, M.R., Azizian, A., et al. (2023). SERPINB3-MYC axis induces the basal-like/squamous subtype and enhances disease progression in pancreatic cancer. Cell Rep. *42*, 113434. https://doi.org/10.1016/j.celrep.2023.113434.

77. Uboveja, A., Huang, Z., Buj, R., Amalric, A., Wang, H., Tangudu, N.K., Cole, A.R., Megill, E., Kantner, D., Chatoff, A., et al. (2024). αKG-mediated carnitine synthesis promotes homologous recombination via histone acetylation. Preprint at bioRxiv. https://doi.org/10.1101/2024.02.06.578742.

78. Liao, C., Zhang, Y., Fan, C., Herring, L.E., Liu, J., Locasale, J.W., Takada, M., Zhou, J., Zurlo, G., Hu, L., et al. (2020). Identification of BBOX1 as a Therapeutic Target in Triple-Negative Breast Cancer. Cancer Discov. *10*, 1706–1721. https://doi.org/10.1158/2159-8290.CD-20-0288.

79. Wang, X., Yang, C., Huang, C., and Wang, W. (2024). Dysfunction of the carnitine cycle in tumor progression. Heliyon *10*, e35961. https://doi.org/10.1016/j.heliyon.2024.e35961.

80. Gietz, R.D., and Schiestl, R.H. (2007). High-efficiency yeast transformation using the LiAc/SS carrier DNA/PEG method. Nat. Protoc. *2*, 31–34. https://doi.org/10.1038/nprot.2007.13.

81. Hubner, N.C., Bird, A.W., Cox, J., Splettstoesser, B., Bandilla, P., Poser, I., Hyman, A., and Mann, M. (2010). Quantitative proteomics combined with BAC TransgeneOmics reveals in vivo protein interactions. J. Cell Biol. *189*, 739–754. https://doi.org/10.1083/jcb.200911091.

82. Rappsilber, J., Mann, M., and Ishihama, Y. (2007). Protocol for micro-purification, enrichment, pre-fractionation and storage of peptides for proteomics using StageTips. Nat. Protoc. *2*, 1896–1906. https://doi.org/10.1038/nprot.2007.261.

83. Cao, X., Lilla, S., Cao, Z., Pringle, M.A., Oka, O.B.V., Robinson, P.J., Szmaja, T., van Lith, M., Zanivan, S., and Bulleid, N.J. (2020). The mammalian cytosolic thioredoxin reductase pathway acts via a membrane protein to reduce ER-localised proteins. J. Cell Sci. *133*, jcs241976. https://doi.org/10.1242/jcs.241976.

84. Cox, J., and Mann, M. (2008). MaxQuant enables high peptide identification rates, individualized p.p.b.-range mass accuracies and proteome-wide protein quantification. Nat. Biotechnol. *26*, 1367–1372. https://doi.org/10.1038/nbt.1511.

85. Cox, J., Neuhauser, N., Michalski, A., Scheltema, R.A., Olsen, J.V., and Mann, M. (2011). Andromeda: a peptide search engine integrated into the MaxQuant environment. J. Proteome Res. *10*, 1794–1805. https://doi.org/10.1021/pr101065j.

86. Cox, J., Hein, M.Y., Luber, C.A., Paron, I., Nagaraj, N., and Mann, M. (2014). Accurate proteome-wide label-free quantification by delayed normalization and maximal peptide ratio extraction, termed MaxLFQ. Mol. Cell. Proteomics *13*, 2513–2526. https://doi.org/10.1074/mcp.M113.031591.

STAR★METHODS

KEY RESOURCES TABLE

REAGENT or RESOURCE	SOURCE	IDENTIFIER
Antibodies		
Mouse monoclonal Actin	Proteintech	Cat# HRP-60008; RRID: AB_2819183
Rabbit polyclonal AFG3L2	Atlas Antibodies	Cat# HPA004480; RRID: AB_1078108
Rabbit monoclonal BBOX1	Abcam	Cat# ab171959
Rabbit polyclonal Citrate synthase	Proteintech	Cat# 16131-1-AP; RRID: AB_1640013
Mouse monoclonal DYKDDDDK (Flag)	Fujifilm Wako Chemicals	Cat# 018-22381; RRID: AB_1065971
Rabbit monoclonal GAPDH	Cell Signalling Technology	Cat# 2118; RRID: AB_561053
Mouse OXPHOS cocktail	Abcam	Cat# ab110411; RRID: AB_275681
Mouse monoclonal p62	MBL International Corporation	Cat# M162-3; RRID: AB_195306
Rabbit polyclonal SLC25A45	Thermo Fisher Scientific	Cat# PA5-62251; RRID: AB_2647421
Rabbit polyclonal TIMM8A	Proteintech	Cat# 11179-1-AP; RRID: AB_2204687
Rabbit monoclonal TOMM20	Abcam	Cat# ab186735; RRID: AB_288997
Mouse monoclonal Ubiquitin	Santa Cruz	Cat# sc-8017; RRID: AB_628423
Mouse monoclonal Vinculin	Sigma-Aldrich	Cat# SAB4200080; RRID: AB_10604160
Alexa Fluor 488 Goat anti-Mouse IgG (H+L)	Thermo Fisher Scientific	Cat# A11001; RRID: AB_2534069
Anti-Rabbit-IgG - Atto 647N	Sigma-Aldrich	Cat# 40839; RRID: AB_1137669
Bacterial and virus strains		
NEB Stable Competent *E. coli*	New England Biolabs	Cat# C3040H
One Shot Stbl3 Chemically Competent *E. coli*	Thermo Fisher Scientific	Cat# C737303
Chemicals, peptides, and recombinant proteins		
Acetonitrile	VWR Chemicals	Cat #83640.320
Ammonium Bicarbonate	Sigma-Aldrich	Cat #09830
Anti-HA Magnetic beads	Thermo Fisher Scientific	Cat# 88837
Anti DYKDDDK tag antibody beads	Fujifilm Wako Chemicals	Cat# 012-22781
L-arginine	Sigma-Aldrich	Cat# A8094
L-Arginine-HCl (13C6; 15N4)	Thermo Fisher Scientific	Cat# 89990
18:1 Cardiolipin	Avanti Research	Cat# 710337
cOmplete EDTA-free protease inhibitor cocktail	Roche	Cat# 04693132001
Cycloheximide	Sigma-Aldrich	Cat# 01810
Digitonin	Sigma-Aldrich	Cat# D141
Dimethyl-L-arginine	Sigma-Aldrich	Cat# SMB00938
DMEM, high glucose, pyruvate, no glutamine	Thermo Fisher Scientific	Cat# 21969-035
EDTA	Sigma-Aldrich	Cat# 03690
EGTA	Sigma-Aldrich	Cat# 324626
Factor Xa Protease	New England Biolabs	Cat# P8010
Fetal Bovine Serum, dialyzed	Thermo Fisher Scientific	Cat# 26400044
Fetal Bovine Serum, qualified	Thermo Fisher Scientific	Ca# 10270106
Freestyle 293 Expression Medium	Thermo Fisher Scientific	Cat# 12338018
Goat Serum	Thermo Fisher Scientific	Cat# 16210064
Hepes	Sigma-Aldrich	Cat# H4034
Iodoacetamide	Sigma-Aldrich	Cat# I1149
Lauryl Maltose Neopentyl Glycol	Anatrace	Cat# NG310

(*Continued on next page*)

Continued		
REAGENT or RESOURCE	SOURCE	IDENTIFIER
LDS Sample Buffer, non reducing	Thermo Fisher Scientific	Cat# 84788
Lipofectamine 2000	Thermo Fisher Scientific	Cat# 11668019
Lipofectamine 3000	Thermo Fisher Scientific	Cat# L3000001
Lipofectamine RNAiMAX	Thermo Fisher Scientific	Cat# 13778075
Lys-C Endoproteinase	Alpha Laboratories	Cat# 125-05061
L-Lysine monohydrate	Sigma-Aldrich	Cat# L9037
L-Lysine:2HCl (13C6, 99%; 15N2, 99%)	Cambridge Isotope Laboratories	Cat# CNLM-2922-H-1
Mannitol	Sigma-Aldrich	Cat# M4125
MEM Amino Acids solution (50x)	Thermo Fisher Scientific	Cat# 11-130-051
MEM Non-Essential Amino Acids solution (100x)	Thermo Fisher Scientific	Cat# 11-140-050
NG,NG'-Dimethyl-L-arginine-D6	LGC	Cat# TRC-D463582
Nepsilon-Methyl-L-lysine hydrochloride	Sigma-Aldrich	Cat# 04685
Nε,Nε,Nε-Trimethyllysine hydrochloride	Sigma-Aldrich	Cat# T1660
Nε,Nε,Nε-Trimethyllysine-d9	LGC Standards	Cat# TRC-T796447
Paraformaldehyde 16%	MPBio	Cat# 199983
Polybrene Infection/Transfection Reagent	Sigma-Aldrich	Cat# TR-1003-G
PEI Max – Transfection Grande Polyethylenimine Hydrochloride	Polysciences	Cat# 24765-100
Polyethylenimine Hydrochloride	Sigma-Aldrich	Cat# 498727
Potassium Phosphate Monobasic	Sigma-Aldrich	Cat# P5655
ProLong Glass Antifade Mountant	Thermo Fisher Scientific	Cat# P36980
Puromycin Dihydrochloride	Thermo Fisher Scientific	Cat# A1113803
Phusion High Fidelity Polymerase	New England Biolabs	Cat# M0530S
QuickExtract DNA Extraction Solution	Bioresearch Technologies	Cat# QE0905T
RPMI 1640 Medium, no glutamine	Thermo Fisher Scientific	Cat# 31870025
Sep-Pak C18 1 cc Vac Cartridge	Waters	Cat# WAT054955
Sodium Deoxycholate	Sigma-Aldrich	Cat# D6750
Sucrose	Fisher Scientific	Cat# S/8600/60
Sequencing Grade Modified Trypsin	Promega	Cat# V5113
Triton X-100	Sigma-Aldrich	Cat# T8787
Critical commercial assays		
GoScript Reverse Transcriptase Mix, Random Primers	Promega	A2801
Histone Extraction Kit	Active Motif	Cat# 40028
NucleoBond Xtra Midi kit	Macherey-Nagel	Cat# 740410-50
Power SYBR Green PCR Master Mix	Thermo Fisher Scientific	Cat# 4367659
RNeasy Mini Kit	Qiagen	Cat# 74104
Seahorse XF Cell Mito Stress Test kit	Agilent	Cat# 103015-100
SuperSignal West Femto Maximun	Thermo Fisher Scientific	Cat# 34095
SuperSignal West Pico PLUS	Thermo Fisher Scientific	Cat# 34580
TMT10plex Isobaric Label Reagent	Thermo Fisher Scientific	Cat# 90309
Deposited data		
Metabolomics data	This study	Massive.ucsd.edu; MSV000098382
Proteomics data – Whole-cell proteome analysis	This study	PRIDE repository: https://www.ebi.ac.uk/pride/archive/projects/PXD058569
Proteomics data – Interactome AP-MS analysis	This study	PRIDE repository: https://www.ebi.ac.uk/pride/archive/projects/PXD058579
Raw imaging data of microscopy, and blots	This study	Mendeley Data: DOI: https://doi.org/10.17632/kgcs7gvccy.1

(*Continued on next page*)

CellPress
OPEN ACCESS

Continued		
REAGENT or RESOURCE	SOURCE	IDENTIFIER
Experimental models: Cell lines		
Human: HEK293-AMPHO	ATCC	Cat# CRL-3213; RRID: CVCL_H716
Human: AsPC-1	ATCC	Cat# CRL-1682; RRID: CVCL_0152
Human: FreeStyle 293-F	Danny Huang lab at CRUK Scotland Institute	RRID: CVCL_D603
Human: HeLa	Thomas Langer lab at Max Planck Institute for Biology of Ageing	RRID: CVCL_0030
Human: HeLa *AFG3L2* KO	Thomas Langer lab at Max Planck Institute for Biology of Ageing	RRID: CVCL_0030
Human: HeLa *SPG7* KO	Thomas Langer lab at Max Planck Institute for Biology of Ageing	RRID: CVCL_0030
Human: Kuramochi	Patricia Roxburgh lab at University of Glasgow	RRID: CVCL_1345
Human: Lenti-X 293T	Takara Bio	Cat # 632180; RRID: CVCL_4401
Human: hTERT-HPNE	ATCC	Cat# CRL-4023; RRID: CVCL_C466
Human: PANC-1	ATCC	Cat# CRL-1469; RRID: CVCL_0480
Human: OVCAR-3	Patricia Roxburgh lab at University of Glasgow	RRID: CVCL_0465
Human: U-2 OS	ATCC	Cat# HTB-96; RRID: CVCL_0042
Experimental models: Organisms/strains		
BJ2168 *Saccharomyces cerevisiae*	ATCC	Cat# 208277
Oligonucleotides		
All oligonucleotides listed in Table S4	This paper	N/A
Recombinant DNA		
pLVX-G418 Vector	This paper	N/A
pMD2.G	Didier Trono	Addgene Plasmid #12259
pLVX-Puro Vector	Takara Bio	632159
psPAX2	Didier Trono	Addgene Plasmid #12260
pcDNA3.1-BBOX1-FLAG	GenScript	U8895YUJG0_1
pcDNA3.1-SLC25A45-FLAG	GenScript	OHu78537D
pcDNA3.1-SLC25A45-Xa-8xHis	GenScript	SC1642
pcDNA3.1-SLC25A45-R285C-FLAG	This paper	N/A
pcDNA3.1-SLC25A45-R268A-FLAG	This paper	N/A
pLVX-EF1a-SLC25A45-FLAG-puromycin	This paper	N/A
pLVX-EF1a-BBOX1-FLAG-G418	This paper	N/A
pMXs-IRES-Bla-3HA-EGFP-OMP25	David Sabatini	Addgene Plasmid #83356
pYES2/CT Yeast Expression Vector	Thermo Fisher Scientific	Cat# V825120
Software and algorithms		
GraphPad Prism 10 (version 10.2.2)	GraphPad Prism 10	N/A
Seahorse Wave Desktop Software	Agilent	N/A
signalsImageArtist (version 1.3.16)	Revvity	N/A
Skyline (version 25.1)	Skyline Software	N/A
Zen Black 3.0	Zeiss	N/A

EXPERIMENTAL MODEL AND STUDY PARTICIPANT DETAILS

Cell lines

AsPC-1, OVCAR-3, and Kuramochi cells were cultured in RPMI-1640 medium with 2 mM GlutaMax, and 10% fetal bovine serum (FBS). U-2 OS, Lenti-X 293T, PANC-1, hTERT-HPNE, and HEK293-AMPHO cells were cultured in DMEM medium with 2 mM GlutaMax, and 10% FBS. HeLa cells were grown in DMEM medium supplemented with 2 mM Glutamax, and 20% FBS. AsPC-1,

OVCAR-3, and Kuramochi cell lines were authenticated by short tandem repeat analysis, and all cell lines were routinely checked for mycoplasma. Cell lines were maintained in a humid atmosphere at 37°C, with 5% CO_2. FreeStyle 293-F cells were cultured in 293 Expression medium, and the cells were kept in 37°C orbital shaker with a humidified atmosphere of 8% CO_2, at 120 rpm. PANC-1 and hTERT-HPNE cell lines were derived from male patients, while all other cell lines were derived from female patients.

Yeast and bacterial strains

Protease-deficient *Saccharomyces cerevisiae* strain BJ2168 was grown in YPG + 0.1% glucose medium in an Applikon Pilot Plant 140-L bioreactor. NEB Stable and One-Shot Stbl3 chemically competent *Escherichia coli* were grown in Luria-Bertani broth and incubated in a 37°C orbital shaker at 120 rpm.

METHOD DETAILS

Plasmid construction

Human SLC25A45 (NM_182556.3) and BBOX1 (NM_001376258.1) were amplified by PCR from plasmids using Phusion High-Fidelity Polymerase, and subcloned into pLVX-EF1a-IRES-puro vector and pLVX-EF1α-IRES-G418, respectively. SLC25A45-Xa-8xHis was synthesized by GenScript and cloned into pLVX-EF1α-IRE-puromycin by PCR. Point mutant SLC25A45 constructs were generated by site-directed-mutagenesis in pcDNA3.1 vectors using PCR, and then subcloned into pLVX-EF1a-IRES-puro vector. The fidelity of all vectors was confirmed by Sanger sequencing. The oligonucleotides used for amplification, or mutation of SLC25A45 and BBOX1 are provided in Table S4.

Cell line transfection

Transient transfection of siRNA or plasmids was performed using Lipofectamine RNAiMax and Lipofectamine 2000 Transfection Reagents, respectively, according to the manufacturer's instructions.

Generation of knockout cells by CRISPR-Cas9 editing

AsPC-1 cells were transfected with pLentiCRISPR v2 encoding *S. pyogenes* Cas9 (SpCas9) and guide RNA (gRNA) targeting *SLC25A45* using Lipofectamine 2000. To isolate monoclonal populations, cells were selected with 1 μg/mL puromycin for 2 days prior to distribution in 96-well plates using a low-density seeding method. CRISPR-Cas9 mediated editing was detected in genomic DNA extracted from monoclonal cultures using the QuickExtract DNA Extraction Solution. Amplicons were generated by PCR, sequenced, and analyzed using the Inference of CRISPR Edits (ICE) analysis tool (Synthego).

Lentivirus production and transduction

To produce stable cell lines, lentivirus was packaged using 2nd generation packaging system plasmids psPAX2 (Addgene Plasmid #12260) and pMD2.G (Addgene Plasmid #12259). Lenti-X 293T cells were co-transfected with the appropriate transfer plasmid, psPAX2 (Addgene Plasmid #12260) and pMD2.G (Addgene Plasmid #12259) using a 3:1 PEI Max 40,000 to DNA ratio. For the generation of Mito-Tag cells, HEK293-Ampho cells were transfected with pMXs-3xHA-EGFP-OMP25 using Lipofectamine 2000. After 48h, the supernatant containing the generated lentivirus was collected and filtered through a 0.45 um polyethersulfone membrane filter syringe. The target cell lines were transduced with the lentivirus accompanied by 10 μg/mL polybrene (Sigma-Aldrich) for 24 h and selected with the appropriate selection antibiotic. AsPC-1 cells were grown in permissive RPMI medium after transduction (RPMI supplemented with 20% FBS, 2 mM GlutaMax, 1X non-essential amino acids, 180 nM uridine) and changed to DMEM supplemented with GlutaMax, and 10% FBS before the experiments.

[^{2}H9]-trimethyllysine [TML] and [^{13}C6, ^{15}N2]-lysine labelling in cells

Cells were seeded in 6-well plates and grown in DMEM supplemented with 10% FBS overnight. For [^{2}H9]-TML labelling, culture media was changed to DMEM medium supplemented with 10% dialyzed FBS for 24 h. Cells were treated with 100 μM [^{2}H9]-TML for the indicated time points before metabolite extraction (detailed below). For [^{13}C6, ^{15}N2]-lysine labelling, cells were incubated with 800 μM lysine or 800 μM [^{13}C6, ^{15}N2]-lysine in DMEM supplemented with 10% dialysed FBS for 72 h.

Mitochondrial isolation

All the steps for mitochondria isolation were performed on ice using pre-chilled buffers. Cells were washed and harvested in PBS by gently scraping. Cell pellets were collected by centrifugation at 1,000 x g for 5 min. Mitochondria were isolated by two methods depending on cell type and application. (A) Differential centrifugation: Cells were resuspended in mitochondria homogenization buffer (5 mM Hepes-KOH pH 7.4, 220 mM mannitol, 70 mM sucrose) prior to homogenization by 15 passes through a 26 gauge needle (AsPC-1) or by 30 strokes using a glass Teflon homogenizer at 1,500 x rpm (FreeStyle 293-F). Unbroken cells and nuclei were removed by centrifugation at 800 x g for 10 min. The post-nuclear supernatant was centrifuged at 8,000 x g for 10 min. The pellet containing the mitochondria and other heavy membrane fractions was resuspended and pelleted again at 8,000 x g for 10 min prior to protein or metabolite extraction; (B) Mitochondrial immunoprecipitation (Mito-IP)[35]: Confluent AsPC-1 cells expressing the 3xHA-EGFP-OMP25 gene (Mito-Tag cells) in a 15 cm dish were washed twice with ice-cold PBS, collected in KPBS (136 mM KCl, 10 mM

CellPress
OPEN ACCESS

KH_2PO_4, pH 7.25, and in Optima LC/MS water) and centrifuged at 1,000 x g for 2 min at 4°C. The pellet was resuspended in 1 mL KPBS, followed by homogenization with 15 passes through a 26 gauge needle. The homogenized sample was centrifuged at 1,000 x g for 2 min at 4°C and the supernatant was incubated with 200 μL anti-HA magnetic beads on a rotor shaker for 3.5 min at 4°C. Mitochondria bound to beads were washed three times with ice-cold KPBS prior to protein or metabolite extraction.

Mitochondrial amino acid uptake assays

Mitochondria were purified using differential centrifugation. Isolated mitochondria were incubated in transport buffer (10 mM Hepes, 0.5 mM EGTA, 136 mM KCl, 10 mM KH_2PO_4, pH 7.25) containing 10 μM [^{2}H9]-TML, 10 μM [^{2}H6]-SDMA/ADMA, 150 μM [^{13}C6, ^{15}N2] lysine, 150 μM [^{13}C6, ^{15}N4]-arginine or amino acid mix (see Table S5) for the indicated times at room temperature. Uptake was stopped by adding ice-cold KPBS, followed by 3 washes with cold KPBS and metabolite extraction (detailed below).

Metabolite extraction from cells and mitochondria

For whole-cell polar metabolite extraction, cells grown in 6 cm plates were washed three times with ice-cold PBS prior to metabolite extraction with extraction solution (50% methanol, 30% acetonitrile, 20% water) chilled at -80°C. The same extraction solution was used to extract metabolites from mitochondrial fractions. Cells and mitochondria were kept under rotation in extraction buffer for 5 min at 4°C and centrifuged for 10 min at 4°C at 12 000 x g. The supernatant was transferred to glass vials and stored at -80°C until analysis by LC-MS. Post-extraction cell plates were taken for protein quantification using the Lowry method.

Metabolite analysis by liquid chromatography-mass spectrometry (LC-MS)

Metabolites were analyzed using Vanquish high-performance liquid chromatography (HPLC) system (Thermo Fisher Scientific) coupled with an Q Exactive orbitrap mass spectrometer (Thermo Fisher Scientific). Briefly, 5μL aliquots of samples were injected onto ZIC-pHILIC guard and analytical column (SeQuant; 150 mm by 2.1 mm, 5 μm; Merck) maintained at 45°C. The mobile phase consisted of 20% ammonium carbonate (20 mM) adjusted to pH 9.2 with ammonium hydroxide solution (25%) (A), and acetonitrile (B). Gradient elution started with 80% B for 2 min, then decreased to 20% at 17 min, then it was returned to 80% at 18 min and held constant till the end of the run at 24.5 at a constant flow rate of 200 μL/min. Data acquisition was performed over a mass range of 75 to 1000 m/z using polarity switching mode with a resolution of 70,000 at 200 mass/charge ratio (m/z). The automatic gain control target and maximal injection time were set at 1e6 and 250 ms, respectively. Peaks were annotated by matching accurate masses, adducts, and retention times to reference standards.

Targeted metabolomics analysis was performed using Skyline software. A transition list containing the compound names, accurate masses, explicit retention times, and polarity was imported into Skyline. Instrument settings were set to orbitrap with a resolution of 70,000. Extracted ion chromatograms were generated for each compound, and its isotopologues using the m/z of the singly charged ions (XIC, +/- 5 ppm), and retention time tolerance (+/- 2 min) and peaks were integrated after careful inspection. Relative quantification between sample groups was performed using raw peak areas. All peak areas were normalized to protein concentration.

Expression of SLC25A45 in Saccharomyces cerevisiae

Human *SLC25A45* (UniProt accession code: Q8N413) was codon-optimized and synthesized (GenScript). To aid expression and purification, the gene was synthesized with an N-terminal extension (GKPRTSPK corresponding to residues 14-21 of the human oxoglutarate carrier, UniProt accession code: Q02978), and an eight-histidine tag and Factor Xa protease cleavage site. These modifications do not affect any important structural or functional elements of the carrier.[7,12] This construct was cloned into the pYES2/CT vector and transformed into the protease-deficient yeast strain BJ2168.[37,80] Successful transformants were selected on Sc-Ura + 2% (w/v) glucose plates. For large-scale expression, a pre-culture of cells grown in Sc-Ura + 2% (w/v) glucose was inoculated into 50 L of YPG + 0.1% glucose medium in an Applikon Pilot Plant 140-L bioreactor. Cells were grown at 30°C for 40 h, induced with 2% galactose, grown for 8 h, and harvested by centrifugation (4,000 × g, 20 min, 4°C). Crude mitochondria were prepared using a bead mill (Dyno-Mill Multilab, Willy A. Bachofen AG) by established methods.[37]

Preparation of lipids for protein purification

Tetraoleoyl cardiolipin (18:1) was dissolved in a 10% (w/v) solution of lauryl maltose neopentyl glycol by vortexing for 4 h, resulting in a concentration of 10 mg mL^{-1} lipid in a 10% (w/v) detergent solution, and stored in liquid nitrogen.

Purification of SLC25A45 by nickel affinity chromatography

Crude mitochondria were solubilized in a solution containing 1.5% (w/v) lauryl maltose neopentyl glycol, an EDTA-free protease inhibitor tablet (Roche), 30 mM imidazole and 150 mM NaCl. The mixture was mixed by rotation at 4°C for 1 h, and the soluble fraction separated from insoluble material by centrifugation (200,000 × g, 45 min, 4°C). Nickel sepharose, at a volume of 0.7 mL slurry per 1 g crude mitochondria, was added to the supernatant. This mixture was stirred at 4°C for 1.5 h, dispensed into an empty PD-10 column, and the settled resin washed with 30 column volumes of buffer A (20 mM HEPES pH 7.0, 150 mM NaCl, 40 mM imidazole, 0.1 mg mL^{-1} tetraoleoyl cardiolipin/0.1% (w/v) lauryl maltose neopentyl glycol), followed by 10 column volumes of buffer B (20 mM HEPES pH 7.0, 50 mM NaCl, 0.1 mg mL^{-1} tetraoleoyl cardiolipin/0.1% (w/v), lauryl maltose neopentyl glycol). The resin was resuspended with 0.7 mL of buffer B and incubated with 20 mM imidazole, 30 μg Factor Xa protease, and 5 mM $CaCl_2$, and on-column cleavage performed overnight at 4°C with rotation. The protein was collected by centrifugation (500 × g, 2 min, 4°C)

and desalted using a midi PD10 desalting column (GE Healthcare), pre-equilibrated with buffer B. The protein concentration was measured by spectrometry (NanoDrop Technologies) at 280 nm (extinction coefficient; 45,775 M^{-1} cm^{-1}, protein mass; 33,118 Da). The purity and stability of the final samples were assessed by gel electrophoresis and thermostability shift assays.

Thermostability analysis

Differential scanning fluorimetry (nanoDSF) was used to assess the thermostability of protein.[39] In this study, the protein was characterized by its tryptophan content (five tryptophans). For the analysis, 10 μg of protein was mixed into 10 μL of buffer B, with or without 10 mM substrate. NanoDSF-grade standard glass capillaries were used to load samples into the Prometheus NT.48. The temperature was progressively increased from 25°C to 95°C, at a rate of 4°C per minute. The software PR. ThermControl (NanoTemper Technologies) was used to determine the apparent melting temperatures (Tm). The change in melting temperature (ΔTm) upon addition of compound was calculated by subtracting the apparent Tm in the absence of compound from the apparent Tm in the presence of compound. A positive ΔTm indicates that a compound binds to and stabilizes protein.

Cycloheximide assay

AsPC-1 cells were seeded in 6-well plates and allowed to adhere overnight. Cells were transfected with the indicated siRNA using Lipofectamine RNAiMAX following the manufacturer's instructions for 24 h. Cell culture medium was changed before treatment of the cells with 150 μg/mL cycloheximide for the indicated time points.

Cell lysis and SDS-PAGE

Cells from 70-80 % confluent wells of 6-well plates were collected in ice-cold PBS and lysed in RIPA buffer (50 mM Tris pH7.4, 150 mM NaCl, 1% Triton X-100, 0.05% deoxycholate, 0.10% SDS, 1mM EDTA pH8.0) for 30 min at 4°C. RIPA lysates were centrifuged at 11,000 x g for 15 min at 4°C to remove insoluble material and protein concentration was measured by Protein assay dye reagent. Samples were heated at 37°C for 10 min or 95°C for 5 min in LDS sample buffer prior to SDS-PAGE and immunoblotting with primary and HRP-coupled secondary antibodies. Antibodies were visualised using a ChemiDoc Imaging System (Bio-Rad) and densitometry was determined using ImageJ Software 1.54k.

Extracellular flux analysis

Oxygen consumption rate (OCR) was measured using a Seahorse XF Pro Analyzer (Agilent). For the cell mito stress test, $3.5x10^4$ cells per well were seeded in XF96 cell culture microplates containing DMEM supplemented with 10% FBS. Meanwhile, the sensor cartridge was hydrated in water at 37°C in a non-CO_2 incubator overnight. The next day, the cell culture medium was replaced by Seahorse XF medium supplemented with 2 mM glutamine and 10 mM glucose, and the plates were incubated for 1 h at 37°C in a CO_2-free incubator. The OCR was measured at basal levels and after sequential injections of 1.5 μM oligomycin, 0.5 μM Carbonyl cyanide-p-trifluoromethoxy phenylhydrazone (FCCP), and 0.5 μM rotenone/antimycin A, according to the manufacturer's instructions. Data were normalized by protein concentration per well using the Bio-Rad Protein assay dye reagent and analyzed by the Seahorse Wave Software (Agilent).

Reverse transcription-quantitative PCR (RT-qPCR)

RNA was extracted from cells using RNeasy Mini Kit following the manufacturer's instructions. cDNA was prepared from 2.5 μg RNA using the GoScript Reverse Transcription Mix, Random Primers. The cDNA samples were subjected to RT-qPCR with Power SYBR Green PCR Master Mix. The data were normalized to expression of the housekeeping gene ACTB and the fold change in expression was calculated with $2^{-\Delta\Delta Ct}$ method. The oligonucleotides used in RT-qPCR are provided in Table S4.

Immunofluorescence and super-resolution microscopy

U-2 OS cells were plated on glass coverslips and allowed to adhere overnight. Cells were transfected with pLVX-EF1a-SLC25A45-puromycin using Lipofectamine 3000 following the manufacturer's instructions for 6 h. Fresh medium was added to the wells and the cells were grown for 48 h. Cells were fixed with 4% paraformaldehyde for 15 minutes at room temperature, washed three times with PBS, and permeabilized with 0.2% Triton X-100 in PBS for 5 minutes at room temperature. Coverslips were washed three times with PBS and incubated with the primary antibody diluted in working buffer (0.2% BSA, 0.02% Triton X-100, 10 mM glycine, 2% goat serum) for 1 h at room temperature. Coverslips were washed three times with PBS before incubation with the secondary antibody in working buffer for 1h at room temperature. The samples were washed three times with PBS and mounted on slides with Prolong Glass for imaging. Imaging was performed on a Zeiss Elyra 7 widefield microscope with Structured Illumination Microscopy (SIM). Images were acquired using 1024x1024 or 256x256 pixels (0.063 μm/pixel), with optimal Z-sectioning (0.11 μm) with a Plan-Apochromat 63x/1.4 Oil DIC M27 Objective. 642 nm (3%, 500mW rated) and 488 nm (5%, 500mW rated) laser lines were used with the following filtersets: BP 570-620 + LP 655 and BP 420-480 + BP 495-550. An exposure time of 50ms was used with 13 Phases, G5 grating period (27.5 μm) for the 488 channel and G3 grating period (36.5 μm) for the 642 channel. Emitted light was captured onto PCO Edge 4.2M sCMOS cameras using a duolink adaptor and cameras were aligned prior to acquisition using the in-built alignment

procedure in Zen software. Images were acquired and SIM[2] processed (3D, 16 iterations, Low Input SNR, regularization 0.065, input and output sampling set to x4, median filter, with resultant xy scaling 0.016 μm) using Zen Black 3.0 SR.

For quantification of SLC25A45-FLAG intensity in mitochondria, HeLa cells were seeded onto 96-well plates and imaged on an Opera Phenix High Content Screening System (Revvity) using a 20x/1.0 water objective (single plane). Imaging was performed using 405 nm (DAPI), 488 nm (SLC25A45-FLAG) and 640 nm (TOMM20) excitation. The analysis was performed using the Signals Image Artist (Revvity). DAPI channel was used to define nucleus mask on structures above 30 μm^2 in area. Mitochondria mask was defined using TOMM20 stained regions upon exclusion of the nuclei and border objects were removed from the analysis. Regions with brighter SLC25A45-FLAG pixels overlapping with mitochondria mask were selected via thresholding on 488 channel (>350) and the mean intensity of fluorescence measured per cell.

Proteomics

Sample preparation for whole-cell mass Spectrometry

Cells were washed in PBS and resuspended in lysis buffer (100 mM Tris-HCl, pH8.0 and 2% sodium dodecyl sulfate in LC-MS water). Samples were sonicated for 5 minutes in 5 s pulse mode at 30% amplitude. Protein lysate was centrifuged at 16 000 x g, for 5 minutes, at room temperature and the protein concentration was determined using the Pierce BCA Protein Assay kit.

Sample preparation for Immunoprecipitation-Mass Spectrometry (IP-MS)

Isolated mitochondria (1 mg) were solubilized in digitonin lysis buffer (5g digitonin/g protein, 25 mM Tris-HCl, pH 7.5, 150 mM NaCl in LC-MS water) and incubated with equilibrated Anti-DYKDDDDK-tag agarose beads (Fujifilm Wako Chemicals) for 2h at 4°C under rotation. Beads were centrifuged at 500 x g, washed in wash buffer (25 mM Tris-HCl (pH7.5), 150 mM NaCl) four times before resuspension in a 2M Urea and 100 mM ammonium bicarbonate buffer and stored at -20°C. On-bead digestion was performed with Lys-C (Alpha Laboratoires) and Sequencing grade modified trypsin (Promega) as previously described[81] and desalted using StageTip.[82]

Sample preparation for Proteome analysis

Proteins were reduced with 10mM DTT and subsequently alkylated in the dark with 55 mM Iodoacetamide (Sigma-Aldrich), both reactions were carried out at room temperature. Alkylated proteins were precipitated adding 4 volumes of acetone at -20°C overnight. Washed pellets were reconstituted in 50 μL of HEPES 200 mM and digested first with Endoproteinase Lys-C (ratio 1:33 enzyme:lysate) for one hour, followed by a sequencing grade modified trypsin (Promega), overnight (ratio 1:33 enzyme: lysate). The digested peptides from each experiment were differentially labelled using TMT10-plex reagent (Thermo Fisher Scientific). Each sample was labelled with 0.1 mg of TMT reagent dissolved in 50 μL of 100% anhydrous acetonitrile. The reaction was carried out at room temperature for 2 hours. Fully labelled samples were mixed in equal amount and desalted using a 50 mg Sep Pak C18 reverse phase solid-phase extraction cartridges (Waters).TMT-labelled peptides were fractionated using high pH reverse phase chromatography on a C18 column (150 × 2.1 mm i.d. - Kinetex EVO (5 μm, 100 Å)) on a HPLC system (Agilent, LC 1260 Infinity II, Agilent). A two-step gradient was applied, from 1–28% B in 42 min, then from 28–46% B in 13 min to obtain a total of 21 fractions for MS analysis.

UHPLC-MS/MS analysis

Peptides resulting from all samples were separated by nanoscale C18 reverse-phase liquid chromatography using an EASY-nLC II 1200 (Thermo Fisher Scientific) coupled to an Orbitrap Q-Exactive HF (Thermo Fisher Scientific) for proteome samples, or to an Orbitrap Fusion Lumos (Thermo Fisher Scientific) for the IP-MS samples. Elution was carried out at a flow rate of 300 nl/min using a binary gradient with buffer A (2% acetonitrile) and B (80% acetonitrile), both containing 0.1% formic acid. Samples were loaded with 6 μL of buffer A into a 50 cm fused silica emitter (New Objective) packed in-house with ReproSil-Pur C18-AQ, 1.9 μm resin (Dr Maisch GmbH). For both systems the packed emitter was kept at 50°C by means of a column oven (Sonation) integrated into the nanoelectrospray ion source (Thermo Fisher Scientific), and the Xcalibur software (Thermo Fisher Scientific) was used for data acquisition.

MS conditions for Proteome samples

Peptides were eluted using different gradients optimized for three sets of fractions: 1–7, 8–15, and 16–21.[83] Each fraction was acquired for a duration of 190 minutes. A full scan over mass range of 375–1400 m/z was acquired at 60,000 resolution at 200 m/z, with a target value of 3e6 ions for a maximum injection time of 20 ms. Higher energy collisional dissociation fragmentation was performed on the 20 most intense ions selected within an isolation window of 0.8 m/z. Peptide fragments were analyzed in the Orbitrap at 45,000 resolution.

MS conditions for IP-MS samples

Samples from each IP-MS replicate were acquired for a total of 135 minutes. A full scan was measured at a resolution of 60000 at 200 m/z, over mass range of 350-1400 m/z. Fragmentation was triggered for the most intense ions detected in the full scan and performed over a 3 sec cycle time. Ions were selected in the quadrupole, fragmented in the ion routing multipole, and finally analyzed in the Orbitrap at a resolution of 15,000 at 200 m/z. Ions were accumulated for a maximum time of 100 ms, or when the normalized AGC target reached 150%. Ions that have been selected for fragmentation were dynamically excluded for 25 sec.

Data Analysis

The MS Raw data were processed with MaxQuant software[84] version 1.6.14.0 (Proteome) or 2.3.5.0 (IP-MS) and searched with Andromeda[85] against *Homo sapiens* proteome database (42,438 entries). First and main searches were performed with

precursor mass tolerances of 20 ppm and 4.5 ppm, respectively, and MS/MS tolerance of 20 ppm. The minimum peptide length was set to six amino acids and specificity for trypsin cleavage was required, allowing up to two missed cleavage sites. The peptide, protein, and site false discovery rate (FDR) was set to 1 %. Modification by iodoacetamide on Cysteine residues (Carbamidomethylation) were specified as fixed, whereas methionine oxidation and N-terminal acetylation modifications were specified as variable. For the proteome analysis, MaxQuant was set to quantify on "Reporter ion MS2", and TMT10plex was chosen as Isobaric label. MaxQuant corrected the interference between TMT channels using the correction factors provided by the manufacturer. The "Filter by PIF" option was activated and a "Reporter ion tolerance" of 0.003 Da was used. For the IP-MS experiment, protein abundance was measured using the label-free quantification algorithm available in MaxQuant.[86] From both experiments, the proteinGroups.txt file from MaxQuant output was used for protein quantitation analysis using Perseus software version 1.6.15.0.[84] The "Reverse", "Potential Contaminants" and "Only identified by site" proteins, as specified in MaxQuant, were removed, as well as protein groups identified with no unique peptides. Only proteins robustly quantified in all replicates in at least one group, were allowed in the list of quantified proteins. Significantly enriched proteins were selected using a permutation-based Student's t-test with FDR set at 5%.

QUANTIFICATION AND STATISTICAL ANALYSIS

All statistical analysis was performed using GraphPad Prism software (v10.2.2) unless stated otherwise. Figure legends define the statistical tests used and what *n* represents for each experiment.

Molecular Cell, Volume *85*

Supplemental information

SLC25A45 is required for mitochondrial uptake of methylated amino acids and *de novo* carnitine biosynthesis

Marilia M. Dias, Martin S. King, Engy Shokry, Sergio Lilla, Nikki Paul, Peter Thomason, Sara Zanivan, David Sumpton, Edmund R.S. Kunji, and Thomas MacVicar

A

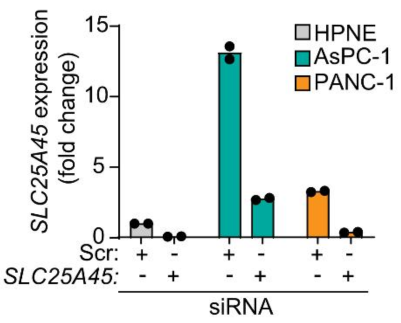

B

ICE Analysis of AsPC-1 *SLC25A45* KO clones

Clone	Guide Target	PAM sequence	Indel %	Model Fit (R^2)	Knockout-Score
#1	GATGAAGTAGATCCCCACCG	TGG	95	0.95	95
#2	GATGAAGTAGATCCCCACCG	TGG	92	0.92	92
#3	GATGAAGTAGATCCCCACCG	TGG	92	0.92	92
#4	GATGAAGTAGATCCCCACCG	TGG	91	0.91	70

Relative contribution of each sequence (normalised)

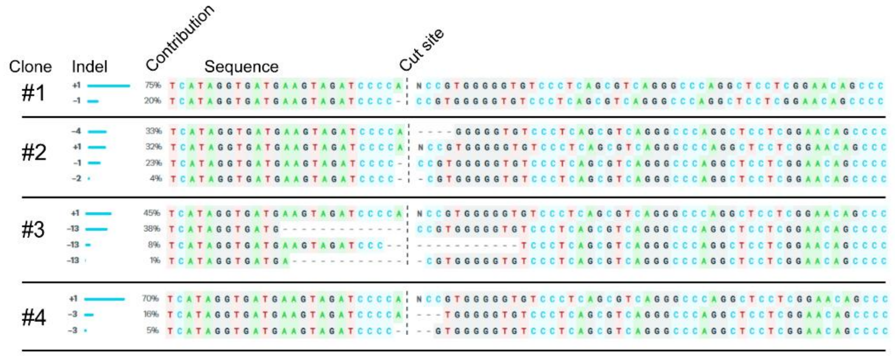

C

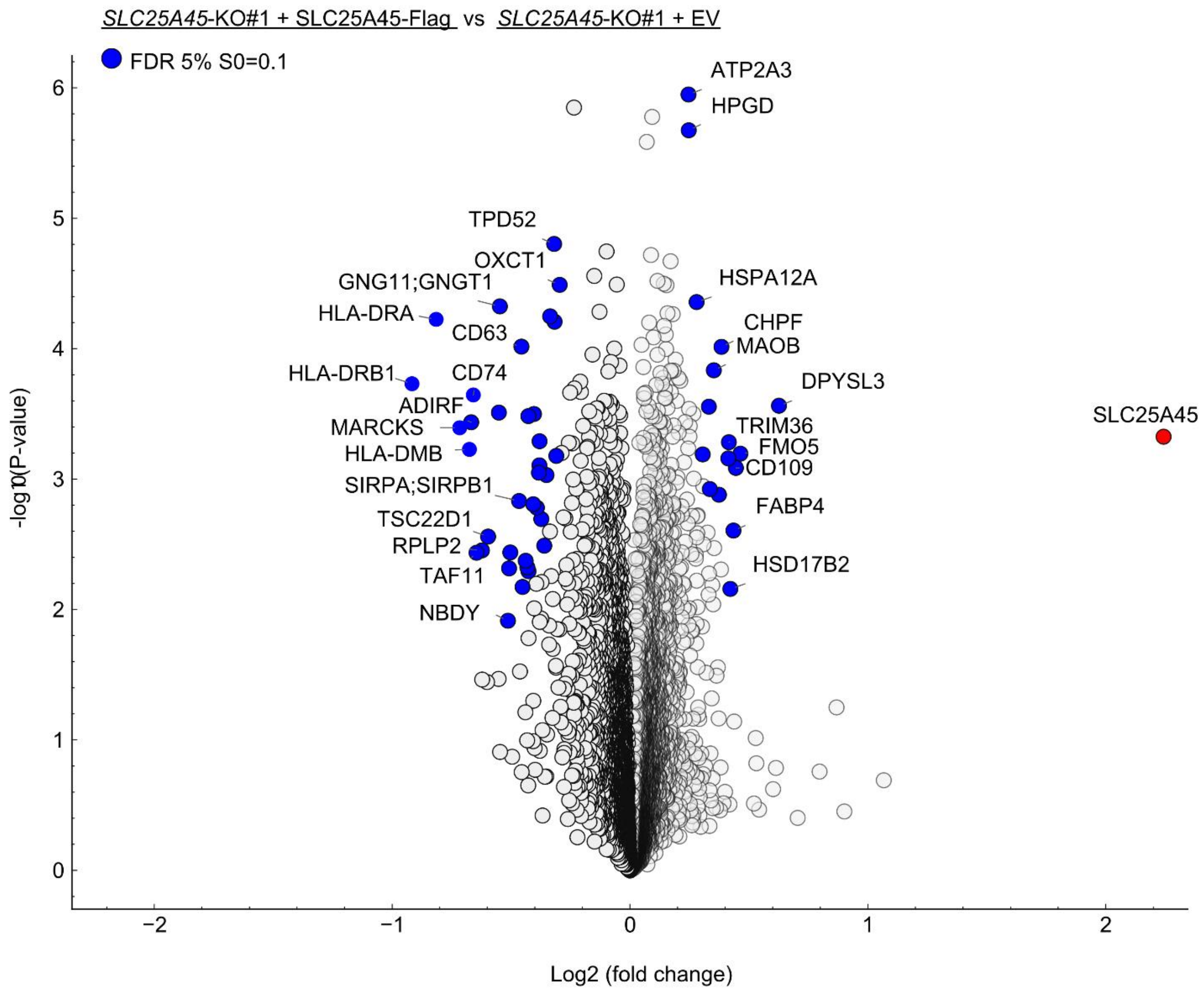

Figure S1. Characterisation of SLC25A45 depleted AsPC-1 cells. Related to Figure 1. (A) *SLC25A45* expression determined by RT-qPCR in the indicated cell lines treated with Scr or *SLC25A45* siRNA for 72 h (n=2). HPNE are hTERT-immortalized epithelial cells from a normal pancreas. AsPC-1 and PANC-1 are pancreatic cancer cell lines. **(B)** Inference of CRISPR Edits (ICE) analysis of *SLC25A45* KO AsPC-1 monoclones generated by CRISPR-Cas9 (https://ice.synthego.com). **(C)** Volcano plot of log2 fold change in protein abundance in *SLC25A45* KO + SLC25A45-FLAG AsPC-1 cells compared with *SLC25A45* KO + EV (empty vector) AsPC-1 cells as measured by LC-MS (n=3; FDR, false discovery rate). Data represent means ± SD; n=independent cultures.

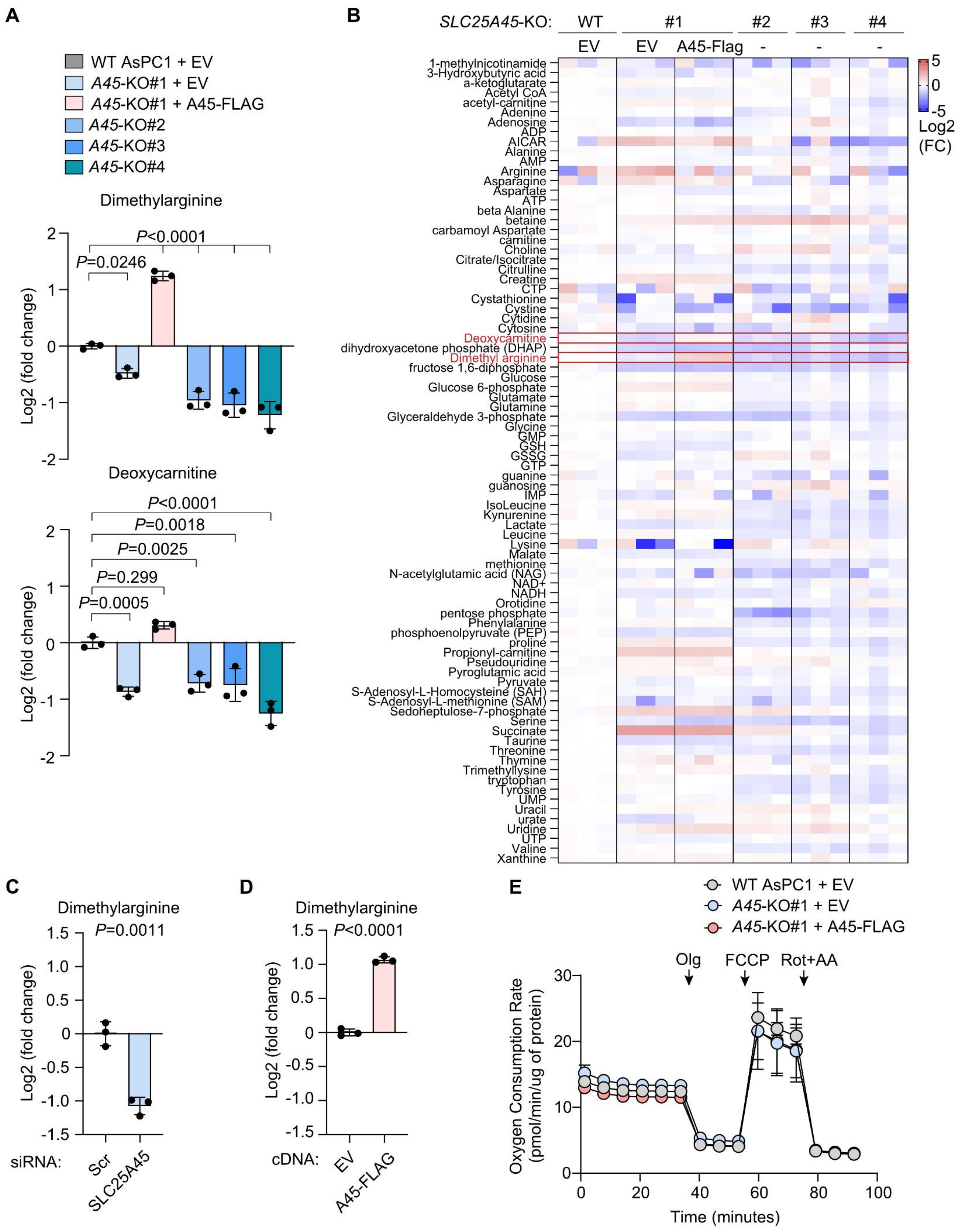
A
WT AsPC1 + EV
A45-KO#1 + EV
A45-KO#1 + A45-FLAG
A45-KO#2
A45-KO#3
A45-KO#4
Dimethylarginine
P<0.0001
P=0.0246
Log2 (fold change)
Deoxycarnitine
P<0.0001
P=0.0018
P=0.0025
P=0.299
P=0.0005
Log2 (fold change)
B
SLC25A45-KO: WT #1 #2 #3 #4
EV EV A45-Flag - - -
1-methylnicotinamide
3-Hydroxybutyric acid
a-ketoglutarate
Acetyl CoA
acetyl-carnitine
Adenine
Adenosine
ADP
AICAR
Alanine
AMP
Arginine
Asparagine
Aspartate
ATP
beta Alanine
betaine
carbamoyl Aspartate
carnitine
Choline
Citrate/Isocitrate
Citrulline
Creatine
CTP
Cystathionine
Cystine
Cytidine
Cytosine
Deoxycarnitine
dihydroxyacetone phosphate (DHAP)
Dimethyl arginine
fructose 1,6-diphosphate
Glucose
Glucose 6-phosphate
Glutamate
Glutamine
Glyceraldehyde 3-phosphate
Glycine
GMP
GSH
GSSG
GTP
guanine
guanosine
IMP
IsoLeucine
Kynurenine
Lactate
Leucine
Lysine
Malate
methionine
N-acetylglutamic acid (NAG)
NAD+
NADH
Orotidine
pentose phosphate
Phenylalanine
phosphoenolpyruvate (PEP)
proline
Propionyl-carnitine
Pseudouridine
Pyroglutamic acid
Pyruvate
S-Adenosyl-L-Homocysteine (SAH)
S-Adenosyl-L-methionine (SAM)
Sedoheptulose-7-phosphate
Serine
Succinate
Taurine
Threonine
Thymine
Trimethyllysine
tryptophan
Tyrosine
UMP
Uracil
urate
Uridine
UTP
Valine
Xanthine
5
0
-5
Log2 (FC)
C
Dimethylarginine
P=0.0011
Log2 (fold change)
siRNA: Scr SLC25A45
D
Dimethylarginine
P<0.0001
Log2 (fold change)
cDNA: EV A45-FLAG
E
WT AsPC1 + EV
A45-KO#1 + EV
A45-KO#1 + A45-FLAG
Olg FCCP Rot+AA
Oxygen Consumption Rate (pmol/min/ug of protein)
Time (minutes)

Figure S2. SLC25A45 loss and overexpression alters dimethylarginine levels in AsPC-1 cells. Related to Figure 1. (A) Log2 fold change in dimethylarginine and deoxycarnitine levels in the indicated AsPC-1 *SLC25A45* knockout (*A45*-KO) clones compared with WT AsPC-1 + EV (empty vector) (n=3). **(B)** Heatmap representation of log2 fold changes in polar metabolites from LC-MS analysis of the indicated AsPC-1 *SLC25A45* knockout (*A45*-KO) clones compared with WT AsPC-1 + EV. Each column represents one experiment. Deoxycarnitine and dimethylarginine are highlighted and these data are also presented in A. **(C)** Log2 fold change in dimethylarginine levels in AsPC-1 cells treated with Scr or *SLC25A45* siRNA for 72 h (n= 3). **(D)** Log2 fold change in dimethylarginine levels in WT AsPC-1 cells expressing EV (empty vector) or SLC25A45-FLAG (n=3). **(E)** Oxygen consumption rates in the indicated cell lines measured by Seahorse extracellular flux analysis and mitochondrial stress test. Wells were injected with oligomycin (Olg), FCCP, rotenone and antimycin A (Rot+AA) at the indicated timepoints (n=2). Data represent means ± SD; n=independent cultures (A-D) or independent experiments (E). *P*-values calculated using one-way ANOVA with Tukey's multiple comparison test (A) or two-tailed unpaired t-test (C,D).

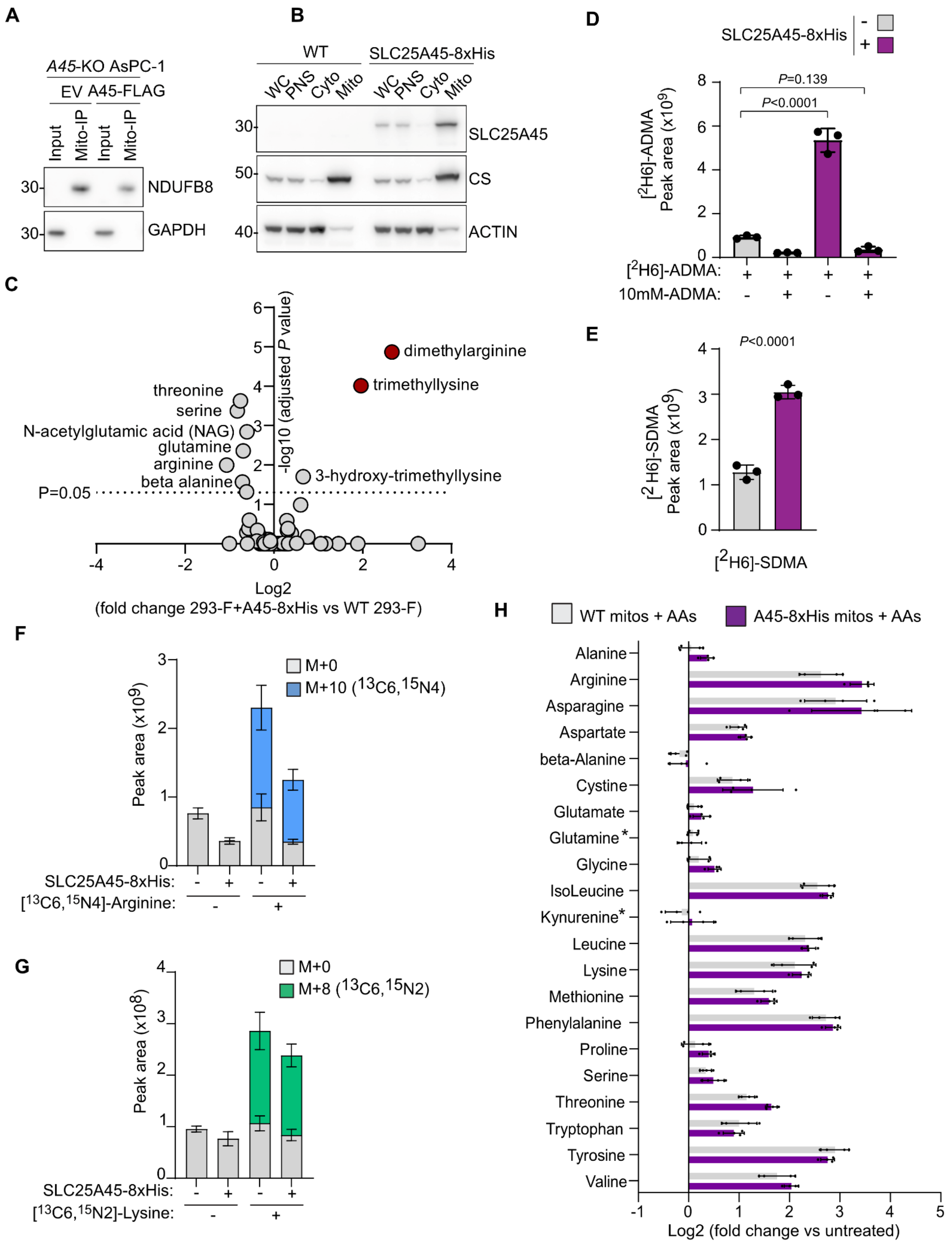

A
A45-KO AsPC-1
EV A45-FLAG
Input
Mito-IP
Input
Mito-IP
NDUFB8
GAPDH
B
WT
SLC25A45-8xHis
WC
PNS
Cyto
Mito
SLC25A45
CS
ACTIN
C
dimethylarginine
trimethyllysine
threonine
serine
N-acetylglutamic acid (NAG)
glutamine
arginine
beta alanine
3-hydroxy-trimethyllysine
P=0.05
-log10 (adjusted P value)
Log2
(fold change 293-F+A45-8xHis vs WT 293-F)
D
SLC25A45-8xHis
P=0.139
P<0.0001
[2H6]-ADMA Peak area (x10^9)
[2H6]-ADMA:
10mM-ADMA:
E
P<0.0001
[2 H6]-SDMA Peak area (x10^9)
[2H6]-SDMA
F
M+0
M+10 (13C6,15N4)
Peak area (x10^9)
SLC25A45-8xHis:
[13C6,15N4]-Arginine:
G
M+0
M+8 (13C6,15N2)
Peak area (x10^8)
SLC25A45-8xHis:
[13C6,15N2]-Lysine:
H
WT mitos + AAs
A45-8xHis mitos + AAs
Alanine
Arginine
Asparagine
Aspartate
beta-Alanine
Cystine
Glutamate
Glutamine*
Glycine
IsoLeucine
Kynurenine*
Leucine
Lysine
Methionine
Phenylalanine
Proline
Serine
Threonine
Tryptophan
Tyrosine
Valine
Log2 (fold change vs untreated)

Figure S3. Analysis of amino acid uptake in isolated mitochondria. Related to Figure 1. (A) Representative immunoblot of mitochondria isolated by immunoprecipitation from *SLC25A45*-KO + EV (empty vector) and *SLC25A45*-KO + SLC25A45 FLAG AsPC-1 cells expressing 3XHA-EGFP-OMP25 used for metabolomic analysis in Figure 1B. Input = whole-cell lysate. NDUFB8 (NADH:ubiquinone oxidoreductase subunit B8) was used as a mitochondrial marker and GAPDH as a cytosolic marker. **(B)** Representative immunoblot of mitochondria isolated by differential centrifugation from wildtype (WT) and SLC25A45-8xHis expressing FreeStyle 293-F cells used for metabolite uptake assays in C-H and Figure 1C,D. WC, whole cell; PNS, post-nuclear supernatant; Cyto, cytosolic fraction; Mito, mitochondria. **(C)** Volcano plot of log2 fold change in mitochondrial metabolites against -log P values isolated from FreeStyle 293-F cells + SLC25A45-8xHis compared with WT FreeStyle 293-F cells (n=4). **(D)** Mitochondrial [^{2}H6]-asymmetric dimethylarginine (ADMA) levels after treatment of mitochondria isolated from the indicated FreeStyle293-F cells with 10 µM [^{2}H6]-ADMA +/- 10 mM unlabelled ADMA for 10 min (n= 3). **(E)** Mitochondrial [^{2}H6]-symmetric dimethylarginine (SDMA) levels after treatment of mitochondria isolated from the indicated FreeStyle293-F cells with 10 µM [^{2}H6]-SDMA for 10 min (n= 3). **(F)** Abundance and mass isotopologue distribution of arginine in mitochondria isolated from the indicated FreeStyle293-F cells and treated with 150 µM [^{13}C6,^{15}N4]-arginine for 10 min (n=4). **(G)** Abundance and mass isotopologue distribution of lysine in mitochondria isolated from the indicated FreeStyle293-F cells and treated with 150 µM [^{13}C6,^{15}N2]-lysine for 10 min (n=4) **(H)** Amino acid levels in mitochondria isolated from the indicated FreeStyle293-F cells treated with amino acid mix for 10 min (see Table S5 for amino acid mix composition; * = amino acids not included in the amino acid mix). Data presented as log2 fold change compared to untreated mitochondria isolated from each cell line. All statistical comparisons returned P>0.05 (n=4). Data represent means ± SD; n=independent mitochondrial preparations. *P* values were calculated using multiple two-tailed unpaired t-tests with Holm-Šídák multiple comparison correction (C and H), two-way ANOVA with Tukey's multiple comparison test (D) or two-tailed unpaired t-test (E).

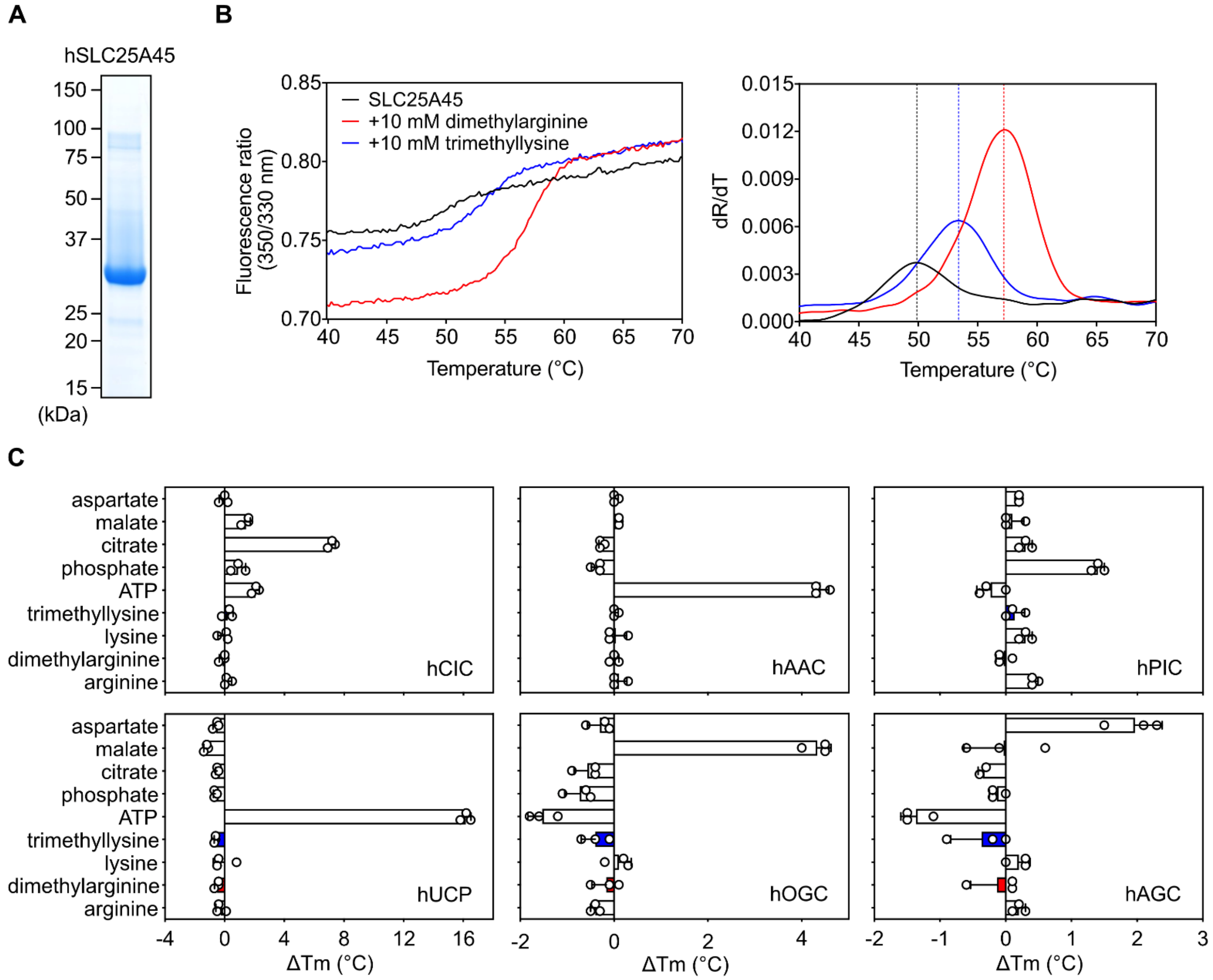

Figure S4. Thermostability shift assays with purified SLC25 proteins. Related to Figure 1. (A) Instant-blue stained SDS-PAGE gel of purified human SLC25A45. **(B)** Representative unfolding curves of ~10 µg protein without compound (black trace), or with 10 mM dimethylarginine (red trace) or 10 mM trimethyllysine (blue trace). The apparent melting temperatures (Tm), indicated by the dashed line, correspond to the peak in the derivative of the unfolding curve (dR/dT). **(C)** The change in melting temperature (ΔTm) of human citrate carrier (hCIC, SLC25A1), human ADP/ATP carrier (hAAC, SLC25A4), human phosphate carrier (hPIC, SLC25A3), human uncoupling protein (hUCP, SLC25A7), human oxoglutarate carrier (hOGC, SLC25A11) and human aspartate/glutamate carrier (hAGC, SLC25A13) in the presence of 10 mM compound, as indicated. Data represent means ± SD, n= 3 technical replicates.

```
S/R/R  - potential contact points      GXXXG motif
                                       Proline kink
                                       Matrix gate (Matrix network + Gln brace)
                                       Cardiolipin binding
                                       Cytoplasmic gate (πxxxπ motif + Tyr brace + cytoplasmic network)

sp|Q8N413|S2545_HUMAN     MPVEEFVAGWISGALGLVLGHPFDTVKVRLQTQTTYRGIVDCMVKIYRHESLLGFFKGMS     60
sp|Q8CFJ7|S2545_MOUSE     MPVEEFVAGWISGAVGLVLGHPFDTVKVRLQTQSTYQGIVDCVVKTYRHESVLGFFKGMS     60
tr|D3ZL04|D3ZL04_RAT      MPVEEFVAGWISGAVGLVLGHPFDTVKVRLQTQNTYQGIVDCVVKTYRHESVLGFFKGMS     60
                          **************:******************.**:*****:** *****:********

sp|Q8N413|S2545_HUMAN     FPIASIAVVNSVLFGVYSNTLLVLTATSHQERRAQPPSYMHIFLAGCTGGFLQAYCLAPF     120
sp|Q8CFJ7|S2545_MOUSE     FPIASVALVNSVLFGVYSNTLLALTATSHQERRAQPPSYTNIFIAGCTGGLLQAYCLAPF     120
tr|D3ZL04|D3ZL04_RAT      FPIASVALVNSVLFGVYSNTLLALTATSHQERRAQPPSYTNIFIAGCTGGLLQAYCLAPF     120
                          *****:*:**************.**************** :**:******:*********

sp|Q8N413|S2545_HUMAN     DLIKVRLQNQTEPRAQPGSPPPRYQGPVHCAASIFREEGPRGLFRGAWALTLRDTPTVGI     180
sp|Q8CFJ7|S2545_MOUSE     DLIKVRLQNQTEPRMQISSSMPRYRGPVHCAASILREEGPQGLFRGSWALVLRDTPTLGM     180
tr|D3ZL04|D3ZL04_RAT      DLIKVRLQNQTEPRMQIGSSTPRYRGPVHCAASILKEEGPQGLFRGSWALVLRDTPTLGM     180
                          ************** * .*  ***:*********::****:*****:***.******:*:

sp|Q8N413|S2545_HUMAN     YFITYEGLCRQYTPEGQNPSSATVLVAGGFAGIASWVAATPLDMIKSRMQMDGLRRRVYQ     240
sp|Q8CFJ7|S2545_MOUSE     YFVTYEGLCRQYTPEGQNPSSATVLVAGGFAGIASWITATPFDVIKSRMQMDGLKGRKYG     240
tr|D3ZL04|D3ZL04_RAT      YFVTYEGLCRQYTPEGQNPSSATVLVAGGFAGIASWITATPFDVIKSRMQMDGLKGRKYG     240
                          **:*********************************::***:*:**********: * *

sp|Q8N413|S2545_HUMAN     GMLDCMVSSIRQEGLGVFFRGVTINSARAFPVNAVTFLSYEYLLRWWG   288
sp|Q8CFJ7|S2545_MOUSE     GMLDCMASSFRQEGIGVFFKGMTLNSARAFPVNAATFLSYEYLLRLWR   288
tr|D3ZL04|D3ZL04_RAT      GMLDCMASSFRQEGIGVFFKGMTLNSARAFPVNAATFLSYEYLLRLWR   288
                          ******.**:****:****:*:*:**********.********** *
                                                                     ↑
                                                               Arginine 285
```

Figure S5. SLC25A45 amino acid sequence. Related to Figure 2. Aligned amino acid sequences of human, mouse (*Mus musculus*) and rat (*Rattus norvegicus*) SLC25A45 generated with Clustal Omega Multiple Sequence Alignment (https://www.ebi.ac.uk/jdispatcher/msa/clustalo). Conserved SLC25 functional motifs are highlighted.

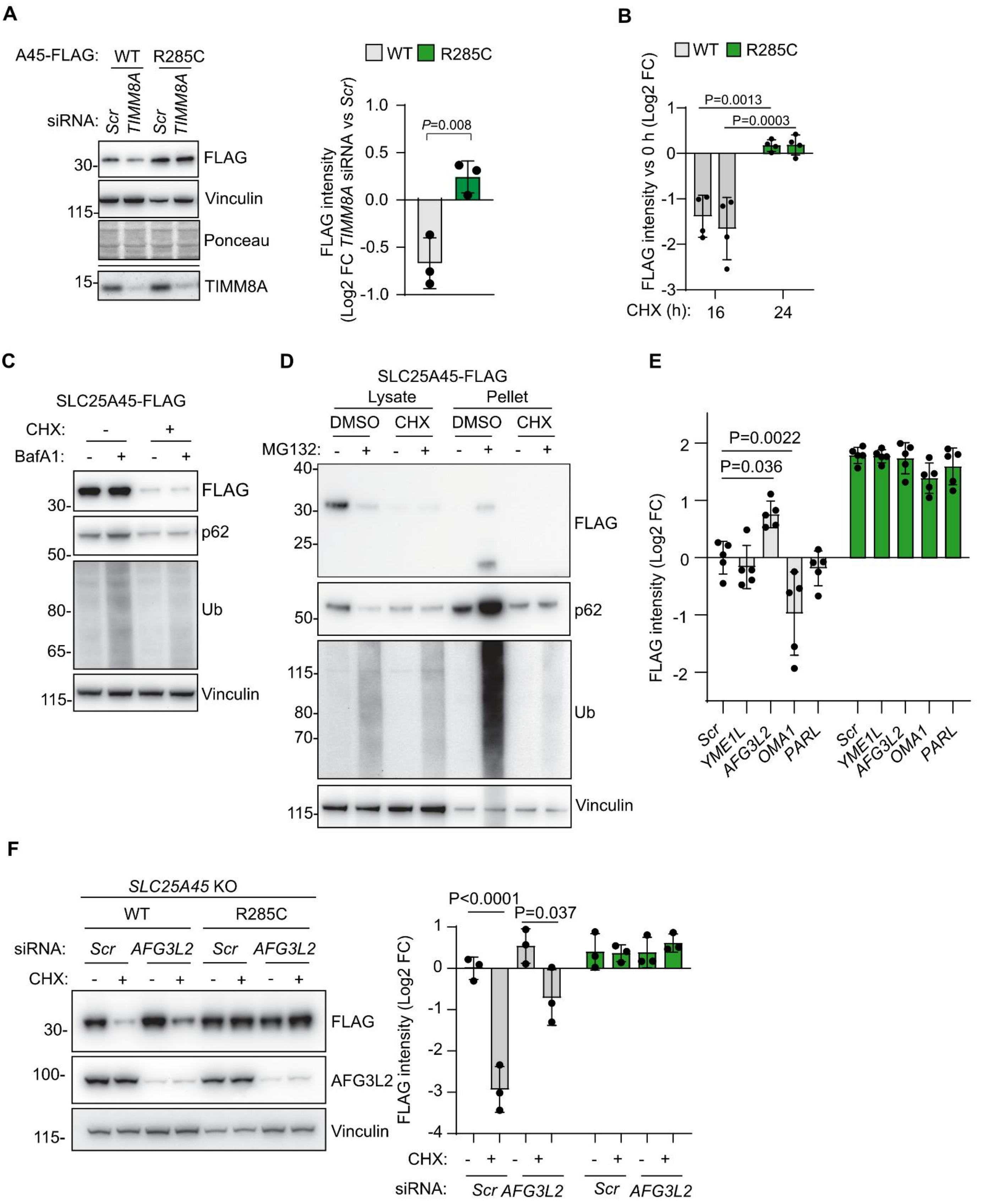

A
A45-FLAG: WT R285C
siRNA: Scr TIMM8A Scr TIMM8A
30-
FLAG
Vinculin
115-
Ponceau
15-
TIMM8A
WT R285C
FLAG intensity (Log2 FC TIMM8A siRNA vs Scr)
P=0.008
1.0
0.5
0.0
-0.5
-1.0
B
WT R285C
FLAG intensity vs 0 h (Log2 FC)
P=0.0013
P=0.0003
CHX (h): 16 24
C
SLC25A45-FLAG
CHX: - +
BafA1: - + - +
FLAG
30-
p62
50-
80-
Ub
65-
Vinculin
115-
D
SLC25A45-FLAG
Lysate Pellet
DMSO CHX DMSO CHX
MG132: - + - + - + - +
40
30
25
FLAG
p62
50
115
80
70
Ub
Vinculin
115
E
P=0.0022
P=0.036
FLAG intensity (Log2 FC)
Scr YME1L AFG3L2 OMA1 PARL Scr YME1L AFG3L2 OMA1 PARL
F
SLC25A45 KO
WT R285C
siRNA: Scr AFG3L2 Scr AFG3L2
CHX: - + - + - + - +
30-
FLAG
100-
AFG3L2
115-
Vinculin
P<0.0001
P=0.037
FLAG intensity (Log2 FC)
CHX: - + - + - + - +
siRNA: Scr AFG3L2 Scr AFG3L2

Figure S6. Stabilisation of SLC25A45 by arginine 268 to cysteine mutation. Related to Figure 2. (A) Immunoblot analysis of *SLC25A45* KO AsPC-1 cells expressing SLC25A45^{WT}-FLAG or SLC25A45^{R285C}-FLAG treated with scrambled (Scr) or *TIMM8A* siRNA for 72 h. Log2 fold change in intensity of FLAG bands are quantified (n=3). **(B)** Log2 fold change in intensity of FLAG bands after CHX treatment. Quantification of data presented in Fig. 2D (n=4). **(C)** Immunoblot analysis of *SLC25A45*-KO + SLC25A45-FLAG AsPC-1 cells treated with 150 µg/mL cycloheximide (CHX) and 200 µM bafilomycin A1 (BafA1) for 16 h (n=1) **(D)** Immunoblot analysis of lysate and pellet fractions from *SLC25A45*-KO + SLC25A45-FLAG AsPC-1 cells treated with 150 µg/mL CHX and 10 µM MG132 for 16 h (n=1) **(E)** Log2 fold change in intensity of FLAG bands compared to scrambled (Scr) siRNA-treated SLC25A45^{WT}-FLAG expressing cells. Quantification of data presented in Fig. 2E. (n=5). **(F)** Immunoblot analysis of *SLC25A45* KO AsPC-1 cells expressing SLC25A45^{WT}-FLAG or SLC25A45^{R285C}-FLAG treated with Scr or *AFG3L2* siRNA for 72 h followed by 24 h CHX. Log2 fold change in intensity of FLAG bands compared to DMSO treated Scr siRNA-treated SLC25A45^{WT}-FLAG expressing cells are quantified (n=3). Data represent means ± SD, n= independent cultures. *P* values calculated using two-tailed unpaired t-test (A) or two-way ANOVA with Tukey's multiple comparison test (B, E, F).

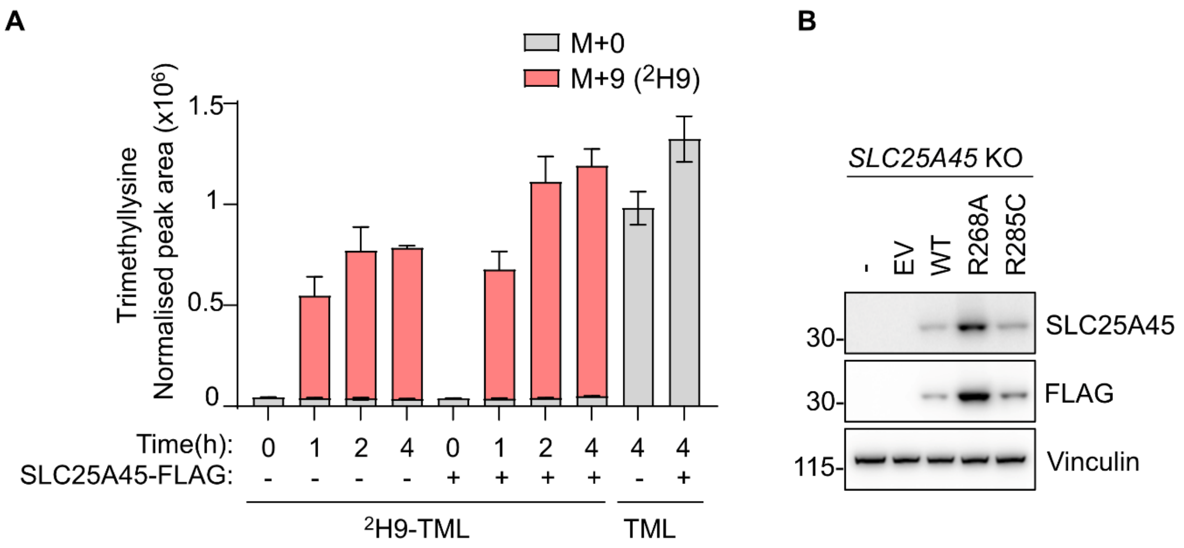

Figure S7. Trimethyllysine labelling of AsPC-1 cells and analysis of SLC25A45 mutants. Related to Figure 3. (A) Normalised abundance and mass isotopologue distribution of trimethyllysine (TML) in *SLC25A45* KO AsPC-1 cells expressing EV or SLC25A45-FLAG treated with 100 µM unlabelled TML or 100 µM [^{2}H9]-TML for the indicated times (n=3 independent cultures). **(B)** Immunoblot analysis of *SLC25A45*-KO AsPC-1 cells transduced with the indicated variants of SLC25A45-FLAG. EV, empty vector. Data represent means ± SD.

A

ICE Analysis of OVCAR-3 and Kuramochi *SLC25A45* KO clones

Clone	Guide Target	PAM sequence	Indel %	Model Fit (R^2)	Knockout-Score
OV-#1	GATGAAGTAGATCCCCACCG	TGG	96	0.96	96
OV-#2	GATGAAGTAGATCCCCACCG	TGG	97	0.97	97
KUR-#1	GATGAAGTAGATCCCCACCG	TGG	90	0.91	90
KUR-#2	GATGAAGTAGATCCCCACCG	TGG	91	0.91	91

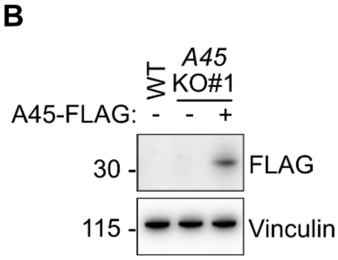

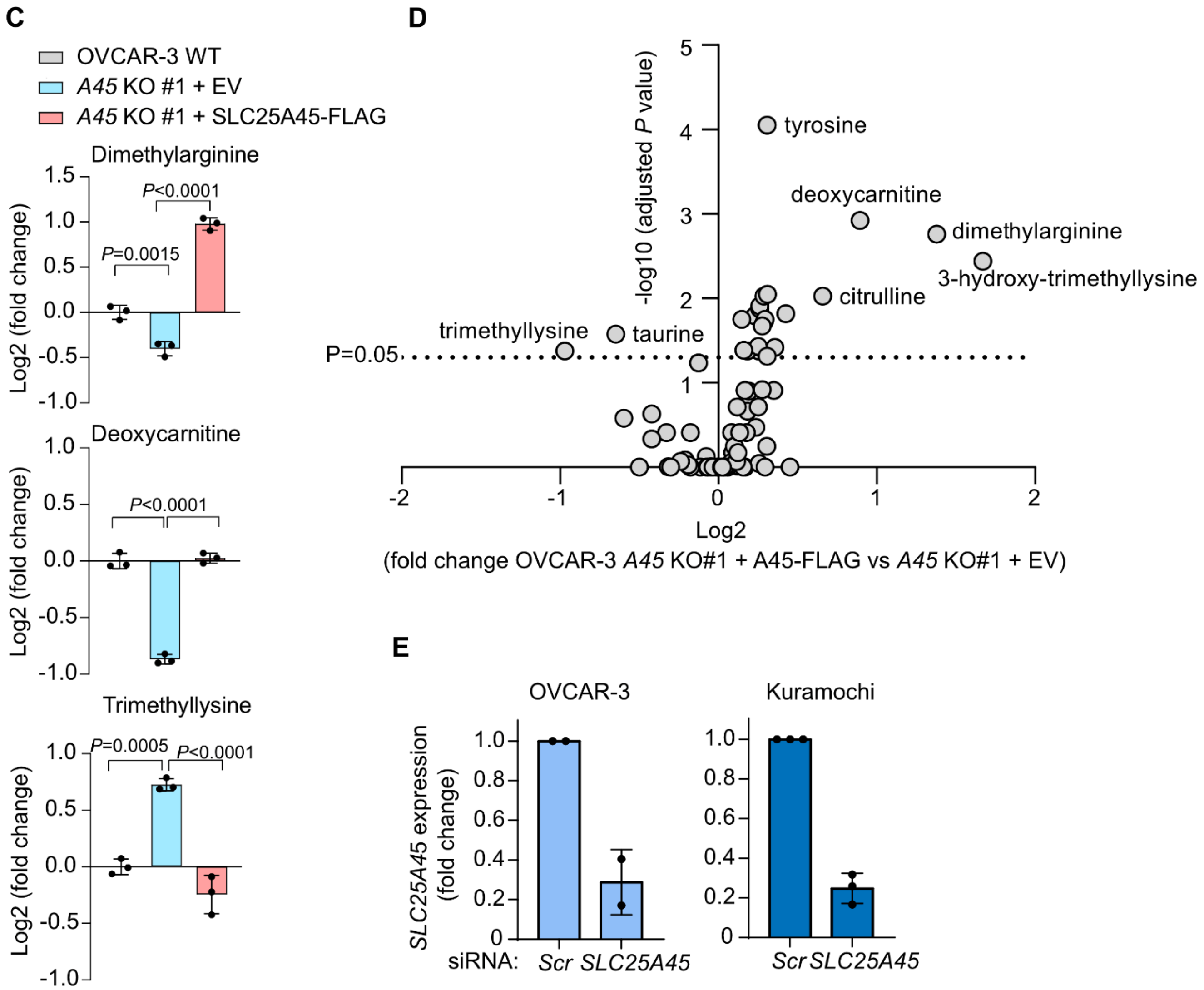

Figure S8. Metabolic analysis of SLC25A45-depleted ovarian cancer cell lines. Related to Figure 4. **(A)** Inference of CRISPR Edits (ICE) analysis of *SLC25A45* KO OVCAR-3 (OV) and Kuramochi (KUR) cells monoclones generated by CRISPR-Cas9 (https://ice.synthego.com). **(B)** Representative immunoblot of indicated OVCAR-3 cells used in C and D. **(C)** Log2 fold change of indicated metabolites in *SLC25A45* knockout + empty vector (*A45*-KO #1 + EV) OVCAR-3 cells and those complemented with SLC25A45-FLAG (*A45*-KO #1 + SLC25A45-FLAG) compared with WT OVCAR-3 (n=3). **(D)** Volcano plot of log2 fold change in whole-cell polar metabolites against -log P values from *SLC25A45*-KO #1 + SLC25A45-FLAG OVCAR-3 cells compared with *SLC25A45*-KO #1 + EV cells. (n=3). **(E)** *SLC25A45* expression determined by RT-qPCR in the indicated cell lines treated with Scr or *SLC25A45* siRNA for 72 h (n=2 for OVCAR-3 and n=3 for Kuramochi). Data represent means ± SD, n=independent cultures. *P* values were calculated using one-way ANOVA with Tukey's multiple comparison test (C) or multiple two-tailed unpaired t-tests with Holm-Šídák multiple comparison correction (D).

Cloning primers			
Gene	Forward primer		Reverse primer
SLC25A45-Flag	TCTAGAATTCATGCCGGTGGAGGAATTT		CTAGGATCCTCACTTATCGTCGTCATCCTT
BBOX1-Flag	GAGGATCTATTTCCGGTGGCCACCATGGCTTGTACCAT		GAGGGAGAGGGGCGGTCAGTTTCCATTCTCCACCCTCTGC
Site-directed mutagenesis			
Gene	Forward primer		Reverse primer
SLC25A45 R268A	CACCATCAACAGTGCCGCCGCCTTTCCCGTCAATGCTG		CAGCATTGACGGGAAAGGCGGCGGCACTGTTGATGGTG
SLC25A45 R285C	TATCTCCTCTGCTGGTGGGGAG		CTCCCCACCAGCAGAGGAGATA
qPCR primers			
Gene	Forward primer		Reverse primer
ACTB	GACGACATGGAGAAAATCTG		ATGATCTGGGTCATCTTCTC
SLC25A45	GTTTGACACTGTAAAGGTGAG		CTATAGACCCCAAACAGGAC
siRNA pools			
Target	Source		Identifier
Scramble	Dharmacon		D-001206-13-05
AFG3L2	Dharmacon		M-005781-00-0005
OMA1	Dharmacon		M-006103-01-0005
PARL	Dharmacon		M-005781-00-0005
SLC25A45	Dharmacon		M-007351-01-0005
TIMM8A	Dharmacon		M-010342-01-0005
YME1L	Dharmacon		M-006103-01-0005
CRISPR gRNA			
Plasmid	Source	Identifier	Sequence
pLentiCRISPR v2 SLC25A45 gRNA#1	GenSript	U1421HF150	GATGAAGTAGATCCCCACCG

Table S4. Oligonucleotide sequences. Related to STAR Methods.

	Amino acid	Final concentration (μM)
**1X MEM Non-Essential Amino Acids**	Glycine	100
	L-Alanine	100
	L-Asparagine	100
	L-Aspartic acid	100
	L-Glutamic acid	100
	L-Proline	100
	L-Serine	100
**1X MEM Amino Acids**	L-Arginine hydrochloride	600
	L-Cystine	100
	L-Histidine hydrochloride	200
	L-Isoleucine	400
	L-Leucine	400
	L-lysine hydrochloride	397
	L-Methionine	101
	L-Phenylalanine	200
	L-Threonine	400
	L-Tryptophan	50
	L-Tyrosine	199
	L-Valine	400

Table S5. Amino acid mix. Related to Figure S3H and STAR Methods.
